# *De novo* variants in *KDM2A* cause a syndromic neurodevelopmental disorder

**DOI:** 10.1016/j.ajhg.2025.12.004

**Published:** 2025-12-29

**Authors:** Eric N. Anderson, Stephan Drukewitz, Sukhleen Kour, Anuradha V. Chimata, Deepa S. Rajan, Senta Schönnagel, Karen L. Stals, Deirdre Donnelly, Siobhan O'Sullivan, John F. Mantovani, Tiong Y. Tan, Zornitza Stark, Pia Zacher, Nicolas Chatron, Pauline Monin, Severine Drunat, Yoann Vial, Xenia Latypova, Jonathan Levy, Alain Verloes, Jennefer N. Carter, Devon E. Bonner, Suma P. Shankar, Jonathan A. Bernstein, Julie S. Cohen, Anne Comi, Deanna Alexis Carere, Lisa M. Dyer, Sureni V. Mullegama, Pedro A. Sanchez-Lara, Katheryn Grand, Hyung-Goo Kim, Afif Ben-Mahmoud, Sidney M. Gospe, Rebecca S. Belles, Gary Bellus, Klaske D. Lichtenbelt, Renske Oegema, Anita Rauch, Ivan Ivanovski, Frederic Tran Mau-Them, Aurore Garde, Rachel Rabin, John Pappas, Annette E. Bley, Janna Bredow, Timo Wagner, Eva Decker, Carsten Bergmann, Louis Domenach, Henri Margot, Undiagnosed Diseases Network, Johannes R. Lemke, Rami Abou Jamra, Julia Hentschel, Heather Mefford, Amit Singh, Udai Bhan Pandey, Konrad Platzer

**Affiliations:** 1 Department of Pediatrics, Children’s Hospital of Pittsburgh, University of Pittsburgh Medical Center, Pittsburgh, PA 15224, USA; 2 Institute of Human Genetics, University of Leipzig Medical Center, 04103 Leipzig, Germany; 3 Department of Biology, University of Dayton, Dayton, OH, USA; 4 Royal Devon & Exeter NHS Foundation Trust, Exeter Genomics Laboratory, Exeter EX2 5DW, UK; 5 Northern Ireland Regional Genetics Centre, Belfast Health and Social Care Trust/City Hospital, Belfast, Northern Ireland BT9 7AB, UK; 6 Division of Child Neurology, Washington University School of Medicine, Mercy Kids Center for Neurodevelopment & Autism, St. Louis, MO 63110, USA; 7 Victorian Clinical Genetics Services, Murdoch Children’s Research Institute, Melbourne, VIC, Australia; 8 Department of Paediatrics, University of Melbourne, Melbourne, VIC, Australia; 9 Epilepsy Center Kleinwachau, 01454 Radeberg, Germany; 10 Department of Medical Genetics, University Hospital of Lyon, 69007 Lyon, France; 11 Department of Genetics, APHP-Robert DEBRE University Hospital, Sorbonne Paris-Cité University, and INSERM UMR, 1141 Paris, France; 12 Stanford Center for Undiagnosed Diseases, Stanford University, Stanford, CA 94305, USA; 13 Department of Pediatrics, Division of Medical Genetics, Stanford University School of Medicine, Stanford, CA 94305, USA; 14 Departments of Pediatrics & Ophthalmology, Genomic Medicine, University of California, Davis Health, Sacramento, CA 95817, USA; 15 Department of Neurology and Developmental Medicine, Kennedy Krieger Institute, Baltimore, MD 21205, USA; 16 Department of Neurology, Johns Hopkins University School of Medicine, Baltimore, MD 21287, USA; 17 GeneDx, LLC, Gaithersburg, MD 20877, USA; 18 Department of Pediatrics, Cedars-Sinai Medical Center, Los Angeles, CA 90048, USA; 19 Department of Neurosurgery, Robert Wood Johnson Medical School, Rutgers University, Piscataway, NJ 08854, USA; 20 Neurological Disorder Research Center, Qatar Biomedical Research Institute, Qatar Foundation, Hamad Bin Khalifa University, Doha, Qatar; 21 Departments of Neurology and Pediatrics, University of Washington School of Medicine, Seattle, WA, USA; 22 Department of Pediatrics, Duke University, Durham, NC, USA; 23 Geisinger Health System, Danville, PA 17821, USA; 24 Department of Genetics, Utrecht University Medical Center, 3584 EA Utrecht, the Netherlands; 25 Institute of Medical Genetics, University of Zurich, 8952 Schlieren, Zurich, Switzerland; 26 Laboratoire de Génomique Médicale – Centre NEOMICS, CHU Dijon Bourgogne, 21000 Dijon, France; 27 INSERM – Université de Bourgogne - UMR1231 GAD, 21000 Dijon, France; 28 Clinical Genetic Services, Department of Pediatrics, NYU School of Medicine, New York, NY 10016, USA; 29 Leukodystrophy Clinic, University Children’s Hospital, University Medical Center, 20246 Hamburg, Germany; 30 Medizinische Genetik Mainz, Limbach Genetics GmbH, Mainz, Germany; 31 Department of Medical Genetics, MRGM INSERM U1211, Bordeaux University Hospital, University of Bordeaux, Bordeaux, France; 32 Center for Rare Diseases, University of Leipzig Medical Center, 04103 Leipzig, Germany; 33 Center for Pediatric Neurological Disease Research, St. Jude Children’s Research Hospital, Memphis, TN 38105, USA; 34 Children’s Neuroscience Institute, Children’s Hospital of Pittsburgh, Pittsburgh, PA 15224, USA

**Keywords:** KDM2A, neurodevelopmental disorder, epigenetic machinery

## Abstract

Germline variants that disrupt components of the epigenetic machinery cause syndromic neurodevelopmental disorders. Using exome and genome sequencing, we identified *de novo* variants in *KDM2A*, a lysine demethylase crucial for embryonic development, in 18 individuals with developmental delays and/or intellectual disabilities. The severity ranged from learning disabilities to severe intellectual disability. Other core symptoms included feeding difficulties; growth issues, such as intrauterine growth restriction, short stature, and microcephaly; and recurrent facial features, such as epicanthic folds, upslanted palpebral fissures, thin vermillion of the lips, and low-set ears. Expression of human disease-causing *KDM2A* variants in a *Drosophila melanogaster* model led to neural degeneration, motor defects, and reduced lifespan. Interestingly, pathogenic variants in *KDM2A* affected physiological attributes, including subcellular distribution, expression, and stability in human cells. Genetic epistasis experiments indicated that *KDM2A* variants act via a dual mechanism—loss of nuclear function for some variants tested and additional cytoplasmic gain-of-function toxicity for c.704C>T (p.Pro235Leu), as eliminating endogenous *Drosophila Kdm2* did not produce noticeable neurodevelopmental phenotypes. Data from enzymatic-methylation sequencing support the suggested gene-disease association by showing aberrant methylome profiles in affected individuals’ peripheral blood. Combining our genetic, phenotypic, and functional findings, we establish *de novo* variants in *KDM2A* as causative for a syndromic neurodevelopmental disorder.

## Teaser

*De novo* variants in *KDM2A* cause a syndromic neurodevelopmental disorder with a phenotypic spectrum of mild to severe developmental delay, feeding difficulties, short stature, microcephaly, and recurrent facial features. Functional studies in human cells and *Drosophila*, as well as aberrant methylation profiles, support the gene-disease association.

## Introduction

The epigenetic machinery encompasses proteins that function as writers, erasers, readers, and remodelers of epigenetic marks on DNA and histones. The eraser KDM2A removes mono- and di-methylation from histone 3 at lysine 36 (H3K36). The post-translational demethylation of lysine residues on histone tails mediated by histone lysine demethylases (KDMs), and specifically by KDM2A, is an important player in gene regulation and has shown to be crucial for embryonic development and processes such as proliferation, apoptosis, and differentiation.^1^

Pathogenic germline variants in genes of the epigenetic machinery cause a now-established group of rare Mendelian disorders.^2^^,^^3^ Within this broad group, variants disrupting KDMs are a frequent cause of neurodevelopmental disorders (NDDs),^4^^,^^5^^,^^6^ which encompass a heterogeneous group of conditions characterized by aberrant brain development and function, leading to cognitive, motor, and behavioral impairments. Previously, two *de novo* missense and one *de novo* frameshift variant in *KDM2A* (MIM: 605657) were described in three individuals with autism and an NDD but only as members of much broader cohorts looking into the genetic architecture of these phenotypes.^7^^,^^8^^,^^9^

Here, we describe the overlapping phenotype of 18 individuals with *de novo* variants in *KDM2A*. We combined the power of genetically modified *Drosophila melanogaster*, which our lab has successfully used to in the delineation of other novel NDDs^10^^,^^11^^,^^12^ with human cell culture, as well as methylome data based on blood-derived DNA from affected individuals, to establish the gene-disease association of *de novo* variants in *KDM2A* and a syndromic NDD.

## Subjects and methods

### Recruitment of affected individuals and consent

This study was approved by the ethics committee of the University of Leipzig (402/16-ek). Written informed consent for molecular genetic testing and data publication was obtained from all individuals and/or their legal representatives by the referring physicians according to the guidelines of the ethics committees and institutional review boards of the respective institutes. After the initial identification of individual 4 with a *de novo* missense variant in *KDM2A*, the compilation of the cohort with *de novo* variants in *KDM2A* and overlapping phenotypes was supported by international collaboration, personal communication, and online matchmaking via GeneMatcher.^13^ Phenotypic and genotypic information was obtained from the referring collaborators using a standardized questionnaire.

### Variant identification

Trio exome or genome sequencing was performed for all affected individuals and their parents except for individuals 12 (duo exome with targeted variant testing of the other parent) and 16 (singleton exome) using standard methodologies and commercial sequencing kits for short-read massively parallel sequencing. Analysis of the sequencing data was performed according to the local diagnostic protocols of the respective centers, primarily focusing on genes where variants are known to cause a rare disease. Since no causative variants were identified in this gene set, research evaluation of the sequencing data was done afterward to potentially identify causative variants in candidate genes not previously associated with a rare disease, such as *KDM2A*. After identification of a rare variant in *KDM2A* in one of the contributing centers, the matchmaking process followed the steps described above to compare genotype and phenotype and potentially contribute to the project. Overall, we were able to recruit 18 individuals with an overlapping neurodevelopmental phenotype, and the identified rare variants were determined to fit the project: proven *de novo* origin for the variant absent in gnomAD v.4 and/or aberrant methylation data supporting a diagnosis of a *KDM2A*-related condition. There were no significant findings, apart from the described variants in *KDM2A*, which likely explain the neurodevelopmental phenotypes of the respective individuals. The gnomAD v.4 dataset served as the control population to determine allele frequency.^14^ All variants described were aligned to hg38, mapped to the *KDM2A* MANE Select transcript GenBank: NM_012308.3, and classified according to ACMG criteria retrospectively in light of the knowledge of the entire cohort (Table S1).^15^^,^^16^ One additional individual is described in the supplemental information and not included in the main cohort (individual S19; for a detailed phenotypic description, see the supplemental notes and Table S4), as the origin of the identified variant could not be tested in the parents, and the analysis of the methylation data did not support a diagnosis of a *KDM2A*-related disorder.

### *In silico* prediction

Missense variants were assessed using CADD-v.1.6,^17^ REVEL,^18^ MutPred2,^19^ VEST4,^20^ and BayesDel^21^ using deleterious prediction cutoffs defined by Pejaver et al.^22^ (Table S2).

### *Drosophila* stock

To model the human variants c.704C>T (GenBank: NM_012308.3) (p.Pro235Leu), c.422A>G (GenBank: NM_012308.3) (p.Tyr141Cys), and c.2431C>A (GenBank: NM_012308.3) (p.His811Asn), the wild-type *KDM2A* (*KDM2A*-WT), *KDM2A*-Pro235Leu, *KDM2A*-Tyr141Cys, and *KDM2A*-His811Asn lines were generated by site-specific insertion of the transgene at BestGene (Chino Hills, CA, USA) using the attP2 insertion vector as previously done.^23^ All *Drosophila* stocks were maintained on standard cornmeal medium at 29°C in light/dark-controlled incubators. ELAV-gal4 (#8760), glass multiple reporter (GMR)-gal4 (#1104), *Kdm2*^TG4^ (#94591), *Kdm2* RNAi (#31360), and luciferase (#35788) were obtained from the Bloomington Drosophila Stock Center (Bloomington, IN, USA).

### Climbing assay

The rapid iterative negative geotaxis (RING) assay was done as previously described.^11^^,^^24^^,^^25^ Briefly, *Drosophila* expressing *KDM2A* variants or luciferase pan-neuronally were aged for 20 days before being transferred to fresh vials. Flies were knocked three times on the base of a bench, and a video camera was used to record the flies climbing up the wall of the vials. The velocity (cm/s) was calculated and analyzed from three independent experiments using GraphPad Prism 6 (Boston, MA, USA).

### Lifespan assay

The lifespan assay was performed as previously described.^11^^,^^24^ Briefly, *Drosophila* expressing *KDM2A* variants or luciferase pan-neuronally were raised on standard cornmeal food. 1- to 3-day-old adult progeny flies were transferred to fresh food twice a week, the number of dead flies was counted every day, and survival functions were calculated and plotted as Kaplan-Meier survival curves. Log rank with Grehan-Breslow-Wilcoxon tests were performed to determine the significance of differences in survival data between the groups using GraphPad Prism 6 software.

### Eye severity experiments in *Drosophila*

A GMR promoter element (GMR-gal4) was used to cross *KDM2A* or luciferase in the eyes. Images of the right eyes from F1-generation adult female *Drosophila* were taken at day 1 using a Leica M205C (Deer Park, IL, USA) dissection microscope equipped with a Leica DFC450 camera. External eye severity was quantified using a previously published scoring system.^26^ Statistical analyses were performed using GraphPad Prism 6, with group comparisons performed using one-way ANOVA.

### *Drosophila Kdm2* knockdown and knockout experiments

To knock down *Kdm2* in the eye, we used the GAL4/UAS system. *Drosophila* carrying the eye-specific driver GMR-Gal4 were combined with UAS-*Kdm2*-RNAi and crossed together with one of the following UAS transgenes in the same background: human *KDM2A*-WT, *KDM2A*-Pro235Leu, *KDM2A*-Tyr141Cys, *KDM2A*-His811Asn, or UAS-luciferase (control). Progeny were reared at 25°C, and day 1 adult eyes were imaged as described above.

*Kdm2* was genetically inactivated by φC31-mediated recombination-mediated cassette exchange (RMCE) of MiMIC insertion lines as previously described.^27^ Briefly, *Drosophila* bearing a MiMIC element within the *Kdm2* locus were crossed to UAS-2×EGFP; hs-Cre; vas-φC31 integrase; Trojan T2A-GAL4 flies following the scheme of Diao et al.^28^ Resulting progeny underwent heat shock to induce Cre-mediated excision and φC31-driven RMCE, replacing the MiMIC cassette with the Trojan T2A-GAL4 reporter to generate *Kdm2*-TG4 alleles. *Kdm2*-TG4 *Drosophila* were then crossed to the same panel of UAS-human *KDM2A* or UAS-luciferase lines. Negative geotaxis (climbing) assays and lifespan analyses were carried out on adult progeny as described above.

### Western blotting

#### *Drosophila*

On day 1, heads from the adult female F1 generation were collected from each cross and snap frozen on dry ice. Five heads were used per lane of the western blots (WBs). Heads were crushed on dry ice and incubated in RIPA buffer containing 150 mM NaCl, 1% NP40, 0.1% SDS, 1% sodium deoxycholate, 50 mM NaF, 2 mM EDTA, 1 mM DTT, 0.2 mM sodium orthovanadate, and 1× protease inhibitor cocktail (Roche, San Francisco, CA, USA; 11836170001). Lysates were sonicated and centrifuged to remove exoskeletal debris. Supernatants were boiled in Laemmli buffer (Boston Bioproducts, Milford, MA, USA; BP-111R) for 5 min, and proteins were separated using 3%–8%, NuPAGE tris-acetate gels (Thermo Fisher Scientific, Waltham, MA, USA: EA03785BOX). Proteins were transferred onto nitrocellulose membranes (iBlot 2 transfer stacks; Invitrogen, Carlsbad, CA, USA; IB23001) using the iBlot2 system (Life Technologies, Waltham, MA, USA; 13120134). Membranes were blocked in milk (BLOT-QuickBlocker reagent; EMD Millipore, Burlington, MA, USA; WB57-175GM) and incubated overnight in primary antibody (rabbit anti-KDM2A, Abcam; ab191387; 1:1000; Waltham, MA, USA). Blots were washed and incubated in secondary antibody for 1 h (anti-rabbit, DYLight 800, Pierce, 1:10,000; Waltham, MA, USA). Imaging was performed using the Odyssey CLx (LI-COR Biosciences, Lincoln, NE, USA). Protein levels were quantified using Image Studio (LI-COR Biosciences), and statistical analyses were performed with GraphPad Prism 6. All WBs were performed in triplicate using biological replicates.

#### Mammalian cells

Human embryonic kidney 293T (HEK293T) cells from ATCC were cultured in advanced Dulbecco’s modified Eagle’s medium (DMEM) (Gibco, Waltham, MA, USA; 12491023) containing 10% FBS (Biowest, Riverside, MO, USA; S01520) and 1× GlutaMAX (Gibco; 35050079). Cells were lysed by boiling for 5 min in 1× LDS sample buffer (Invitrogen; NP-0007) and RIPA buffer. All NuPAGE and western blotting steps were performed as described above. Experiments were performed in triplicate using three independent lysate preparations from cultured cells.

#### Plasmids

*KDM2A*-WT-HA (VB: 220629-1403rdb), *KDM2A*-Pro235Leu-HA (VB220629-1413ecm), *KDM2A*-Tyr141Cys-HA (VB: 220629-1409axj), and *KDM2A*-811N-HA (VB: 220629-1405kmm) were constructed by VectorBuilder (Chicago, IL, USA).

#### Immunofluorescence

HEK293T cells transfected with the *KDM2A* plasmid grown on coverslips were rinsed in PBS (Lonza 17-512F) and fixed in 4% paraformaldehyde (Millipore Sigma P6148, Burlington, MA, USA) for 20 min at room temperature. Following fixation, the samples were washed four times (×10 min) in PBS and blocked with blocking buffer: 5% normal goat serum (NGS; Abcam AB7681) in PBS with 0.1% Triton X-100 (PBST). The samples were incubated overnight at 4°C with primary antibody rabbit anti-*KDM2A* (1:1,000) and mouse anti-HA (H3663; 1:1,000; Millipore Sigma), washed four times (×10 min) with 0.1% PBST, and incubated with secondary antibody (goat anti-mouse Alexa Fluor 568, A22287: 1:500, and goat anti-rabbit Alexa Fluor 488, A-11008: 1:500, Invitrogen) for 2 h at room temperature, followed by 0.1% PBST washes. Samples were mounted onto slides using Fluoroshield (Sigma-Aldrich F6057, St. Louis, MO, USA).

#### Nuclear-cytoplasmic fractionation

HEK293T cells transfected with *KDM2A*-WT or *KDM2A* variants (p.Pro235Leu, p.Tyr141Cys, and p.His811Asn) were harvested, and nuclear-cytoplasmic fractionation was done using the NE-PER nuclear-cytoplasmic extraction kit per the manufacturer’s protocol (Thermo Fisher Scientific).

#### Cycloheximide chase assay

To evaluate the stability of the *KDM2A*-WT and the p.Pro235Leu variant, HEK293T cells were transfected with the *KDM2A* plasmids and incubated for 24 h. Protein synthesis was then inhibited by the addition of 0.5 mg/mL cycloheximide (CHX). At 5 different time points (0, 6, 12, 24, and 48 h after CHX addition), the cells were harvested, and the samples’ supernatants were subjected to immunoblot analysis with HA antibody to recognize KDM2A and loading control Tubulin.

### Methylome analysis and episignature

DNA was extracted from peripheral blood using standard protocols at the respective centers. DNA quantity was measured using Qubit 4 and the Qubit double-stranded DNA (dsDNA) BR Assay-Kit (Thermo Fisher Scientific, Darmstadt, Germany). A total of 200 ng of DNA was used for ultrasonic fragmentation of the DNA with a focused ultrasonicator (ME220, Covaris, MA, USA). Library preparation was done using the NEB Enzymatic Methyl-seq Library Preparation Kit. A 187.5 ng library was used for capture with the Twist Human Methylome Panel according to the manufacturer’s protocol (TWIST Bioscience, South San Francisco, CA, USA). This panel targets 5.549 million CpG sites (in comparison: on the EPIC v.2.0 array, 0.930 million CpG sites are covered; 0.163 million CpGs are unique in the array, while 4.782 million are unique in the methylome panel). 2×150 bp sequencing was done on one lane of an S4 cartridge (s4 reagent kit [300 cycles], run on a NovaSeq6000 [Illumina, San Diego, CA, USA]). The average coverage of target regions was 120×. As a control group, DNA from the peripheral blood of healthy age- and sex-matched individuals was selected. Raw reads were quality checked using FastQC and trimmed using cutadapt.^29^ Trimmed data were aligned to GRCh38 using BWA-meth, and duplicates were marked using Picard – MarkDuplicates. Methylation calls were extracted from the deduplicated alignment files using MethylDackel. Bedgraphs were filtered for regions covered >30×. Calling of differentially methylated regions (DMRs) between case and control groups was done using metilene with the following settings: a minimum (min.) length of CpG of ≥10, a min. length in nt of >0, a min. absolute methylation difference of ≥0.1, and an adjusted *p* [*p*-adj] < 0.05.^30^ To generate an episignature of the *KDM2A* cases, called DMRs, were filtered using an *p*-adj < 0.01 and a minimal absolute methylation difference of 10%.

To visualize the episignature, methylation rates of the DMRs were extracted from the MethylDackel output. Hierarchical clustering and heatmap visualization were performed using the clustermap function implemented from the Seaborn Python package.^31^

## Results

### Clinical description

**Figure 1:**
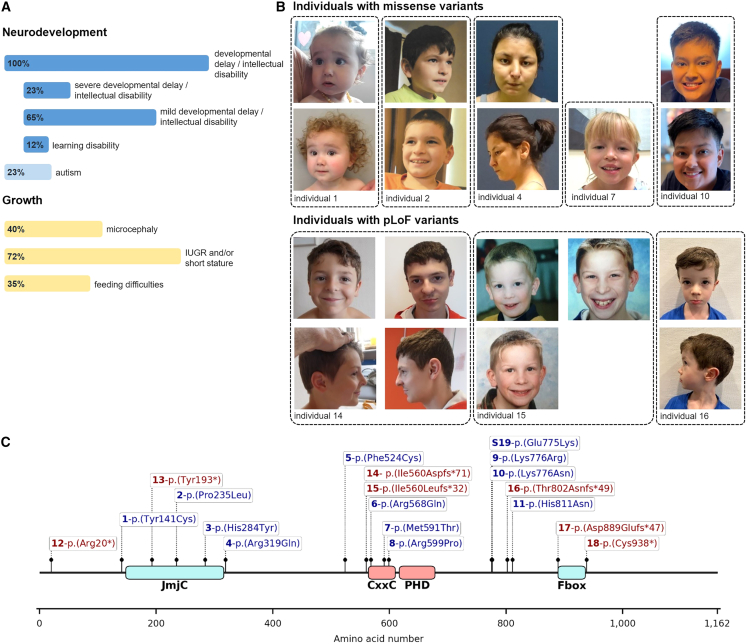
Prevalence of clinical findings and variant location on the protein level (A) Core symptoms of the *KDM2A*-related neurodevelopmental disorder. IUGR, intrauterine growth restriction. (B) Facial appearance of individuals at different ages that harbor missense variants or predicted loss-of-function variants in *KDM2A*. Epicanthus, upslanted palpebral fissures, thin upper and/or lower lips, and low-set ears were noted as recurrent dysmorphic facial features. (C) Linear schematic representation of the KDM2A and location of the variants (GenBank: NM_012308.3). Bold numbers indicate individuals within the cohort. Blue variants represent missense variants, and red variants indicate predicted loss-of-function variants.

Here, we describe a cohort of 18 individuals, including one prenatal subject with *de novo* variants in *KDM2A*. An overview of the clinical data on all individuals is presented in Figure 1A and Table 1 (for a detailed phenotypic description, see the supplemental notes; Figure S1; Table S3).

**Table 1:** Clinical and genetic details of all affected individuals with causative variants in *KDM2A*

**Ind.**	**Age**^a^**(sex)**	**Variant (GenBank: NM_012308.3)**	**Developmental delay/intellectual disability**	**Seizure type (age of onset) outcome**	**Microcephaly**	**Growth, feeding**	**Neurological findings, autism, behavior**	**Dysmorphic features**	**Further findings**
1	5 y (F)	c.422A>G (p.Tyr141Cys), *de novo*	severe	–	+ (primary)	feeding difficulties (tube fed), poor weight gain, short stature	–	midface hypoplasia, bilateral epicanthus, widely spaced and deeply set eyes, low-set and posteriorly rotated ears, saggy cheeks	hypotonia, hyporeflexia
2	8 y (M)	c.704C>T (p.Pro235Leu), *de novo*	severe, no speech	–	–	–	autism	large pinnae, helical deformities, depressed nasal bridge, small jaw, short forehead	–
3	6 y (M)	c.850C>T (p.His284Tyr), *de novo*	mild	–	+ (secondary)	feeding difficulties, poor weight gain, short stature	ADHD	triangular face	–
4	25 y (F)	c.956G>A (p.Arg319Gln), *de novo*	severe, no speech	focal (10 y), not seizure free	–	short stature	dysarthria	upslanted palpebral fissures, narrow mouth with thin upper and lower vermillion of the lip, conical tapered fingers	MRI: polymicrogyria
5	13 y (F)	c.1571T>G (p.Phe524Cys), *de novo*	mild	generalized (1 y), seizure free	+ (primary)	IUGR, feeding difficulties	–	triangular face, pointed chin	–
6	10 y (M)	c.1703G>A (p.Arg568Gln), *de novo*	mild	–	+ (secondary)	IUGR, feeding difficulties (tube fed), short stature	behavioral abnormalities, ADHD	bilateral epicanthus	cryptorchidism
7	6 y (F)	c.1772T>C (p.Met591Thr), *de novo*	mild	–	–	feeding difficulties, short stature	autism, ADHD, aggressive behavior	frontal bossing, broad nasal bridge, bilateral epicanthus, high anterior hairline, temporal narrowing, low-set ears	hypotonia, decreased facial muscle tone
8	3 y (F)	c.1796G>C (p.Arg599Pro), *de novo*	mild	–	+ (secondary)	IUGR, feeding difficulties, low weight, short stature	–	protruding metopic suture, upslanted palpebral fissures, bilateral epicanthus, thin vermillion of the lower lip	delayed temporary teeth eruption
9	3 y (M)	c.2327A>G (p.Lys776Arg), *de novo*	learning disability	–	–	–	autism	–	–
10	13 y (M)	c.2328G>T (p.Lys776Asn), *de novo*	mild	generalized (9 y), not seizure free	–	–	ADHD, sensory/auditory processing disorder	upslanted palpebral fissures	–
11	14 y (M)	c.2431C>A (p.His811Asn), *de novo*	mild	epileptic spasms (5 m), seizure free	–	–	autism, Asperger-like	N/A	hypotonia
12	5 y (M)	c.58C>T (p.Arg20^∗^), *de novo*	mild	–	–	IUGR, short stature, delayed bone age	behavioral abnormalities	bilateral epicanthus, upslanted palpebral fissures, protruding ears	hypotonia
13	prenatal (F)	c.579C>G (p.Tyr193^∗^), *de novo*	N/A	N/A	+ (primary)	IUGR	N/A	N/A	pregnancy terminated
14	13 y (M)	c.1676dup (p.Ile560Aspfs^∗^71), *de novo*	learning disability	–	–	–	dyspraxia, ADHD, impulsivity, anxiety	high palate, thin vermillion of the upper lip	supernumerary nipple
15	25 y (M)	c.1677del (p.Ile560Leufs^∗^32), *de novo*	mild, IQ 64	–	N/A	IUGR	–	micrognathia, mildly upslanted palpebral fissures	umbilical hernia; galbladder, pancreatic, and cardiac malformations; diabetes type 1 (all likely due to *GATA6* variant)^b^
16	6 y (M)	c.2404dup (p.Thr802Asnfs^∗^49), heterozygous	mild	–	–	IUGR, short stature	–	micrognathia, thin upper vermillion of the lip, small midface	–
17	14 y (M)	c.2667del (p.Asp889Glufs^∗^47), *de novo*	severe	focal dyscognitive (4 y), seizure free	N/A	short stature	ADHD, anxiety	–	inverted nipples, bilateral club foot
18	11 y (M)	c.2809_2812dup (p.Cys938^∗^), *de novo*	mild	–	–	IUGR	anxiety	low-set ears, thin upper vermillion of the lip with long philtrum, retrognathia, long and narrow nose	–

ADHD, attention-deficit hyperactivity disorder; DD, developmental delay; F, female; Ind., individual; IUGR, intrauterine growth restriction; M, male; m, months; N/A, not available; y, years; –, not reported. Further clinical details are provided in Table S3.

a Age at last assessment.

b See Table S3 and supplemental notes.

All individuals, excluding the prenatal subject, exhibited developmental delay and/or intellectual disability, with the severity of developmental delay or intellectual disability ranging from learning disabilities to severe intellectual disability. The majority of individuals were affected by mild developmental delay/intellectual disability (11/17) or learning disabilities (2/17), while four individuals presented with severe developmental delay/intellectual disability. Microcephaly was observed in six individuals, including the prenatal subject. Three of the six individuals presented with primary microcephaly and three with secondary microcephaly. Autism was diagnosed in four individuals, while other behavioral abnormalities, such as attention-deficit hyperactivity disorder or aggressive behavior, were noted in seven individuals. Seizures occurred in five individuals, with onsets ranging from 4 months to 10 years. Seizure types included focal and generalized seizures as well as epileptic spasms. At the last assessment, two individuals continued to experience seizures, while three achieved seizure freedom. In the latter three, the anti-seizure medication was subsequently weaned off without relapse. Hypotonia was reported in four individuals. Cranial MRI was performed in 13 individuals, revealing normal results in seven. Minor and nonspecific abnormalities, such as cerebellar tonsillar ectopia and multiple white matter hyperintensities, were reported in four individuals. Of note, individual 4 did present with a malformation of cortical development (MCD) of the polymicrogyria spectrum. None of the individuals was reported to show signs of developmental regression. Another notable recurring phenotype in the cohort was growth abnormalities, including intrauterine growth restriction (IUGR) and/or short stature in thirteen individuals, including the prenatal subject. Eight individuals showed IUGR, and nine individuals developed short stature. Of note, four of these thirteen individuals presented with both IUGR and short stature later in life. Feeding difficulties were observed in six individuals, four of whom experienced these issues during the neonatal period, with two requiring tube feeding. Additionally, two individuals had persistent difficulties gaining weight later in life. The referring clinicians reported dysmorphic facial features in twelve individuals: epicanthus, upslanted palpebral fissures, thin vermillion of the upper and/or lower lips, and low-set ears emerged as recurrent descriptive features that each occurred in at least three individuals (Figure 1B; Table 1).

### Genetic results

Exome and/or genome sequencing revealed *de novo* variants in *KDM2A* in 17 of the 18 affected individuals (Figure 1C). Individual 16 was identified using a singleton exome, but segregation analysis of the *KDM2A* variant in the parents was not available. We identified eleven distinct *de novo* missense variants and seven predicted loss-of-function (pLoF) variants. None of the variants are recurrent, and only two missense variants affect the same amino acid residue, Lys776. All of the identified variants are absent from gnomAD (v.4 dataset).^14^ The eleven missense variants all affect moderate to highly conserved amino acid residues of *KDM2A*, but *in silico* prediction of potential deleteriousness varies within the cohort. Seven of the missense variants are predicted to be damaging by multiple algorithms, while results of the other four variants encompass mixed and benign predictions (Tables S2 and S5).

According to gnomAD, *KDM2A* is a gene with a significantly reduced number of pLoF as well as missense variants, indicating that there is a selective constraint on both types of variants in the general population that lacks severe, early-onset phenotypes such as developmental delay, intellectual disability, microcephaly, or short stature (LOEUF = 0.06; pLI = 1; o/e for missense variants = 0.49; *Z* score = 6.9).^14^

### p.Pro235Leu variant alters nuclear localization and decreases KDM2A levels

**Figure 2:**
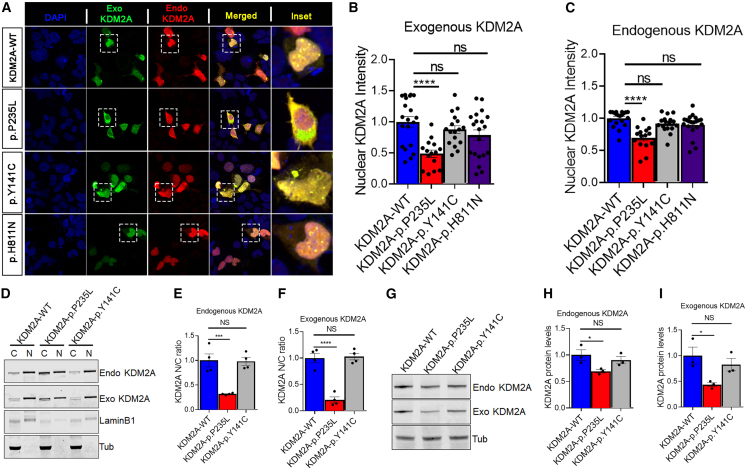
KDM2A variants alter the subcellular distribution of KDM2A in mammalian cells (A) Representative immunofluorescence images of human embryonic kidney 293T cells (HEK293T cells) transfected with HA-tagged wild-type KDM2A (KDM2A-WT) or variants (P235L, Y141C, or H811N) stained for exogenous (green) and endogenous (red) KDM2A. DAPI was used to label nuclei. (B) Exogenous KDM2A nuclear intensity quantification showed that P235L (^∗∗∗∗^*p* < 0.0001), but not Y141C or H811N, showed significantly decreased nuclear intensity compared to exogenous KDM2A-WT (*n* = 14–20 cells). (C) Endogenous KDM2A nuclear intensity quantification showed that P235L (^∗∗∗∗^*p* < 0.0001), but not Y141C or H811N, produced a significant reduction in nuclear intensity of endogenous KDM2A as compared to KDM2A-WT (*n* = 14–20 cells). (D) Western blots of cytoplasmic (C) and nuclear (N) fractions from HEK cells transfected with KDM2A-WT, P235L, and Y141C variants probed for exogenous KDM2A (anti-HA), endogenous KDM2A (anti-KDM2A), nuclear membrane marker (laminB1), and tubulin. (E) Nuclear-cytoplasmic (N/C) ratio quantification of endogenous KDM2A (*n* = 3 blots, ^∗∗∗^*p* < 0.001). (F) N/C ratio quantification of exogenous KDM2A (*n* = 3 blots, ^∗∗∗^*p* < 0.001). (G) Western blots of HEK cells transfected with KDM2A-WT, P235L, and Y141C stained for endogenous KDM2A (anti-KDM2A) and exogenous KDM2A (anti-HA). Tubulin was used as the loading control. (H and I) Western blot quantification of endogenous KDM2A (H) and exogenous KDM2A (I) in HEK cells (^∗^*p* < 0.05, *n* = 3). One-way ANOVA was performed to determine the significance in (B), (C), (E), (F), (H), and (I). All quantifications represent the mean ± SEM.

KDM2A is primarily localized in the nucleus, and its interaction with unmethylated CpG DNA is crucial for maintaining heterochromosomal homeostasis.^32^^,^^33^ To investigate the impact of missense variants on the intracellular localization of KDM2A, we introduced HA-tagged WT and mutant forms (p.Pro235Leu, p.Tyr141Cys, and p.His811Asn) of KDM2A into HEK293T cells. While the expression of WT, p.Tyr141Cys, or p.His811Asn did not visibly affect the distribution and localization of KDM2A, the expression of the p.Pro235Leu variant led to the relocation of predominantly nuclear KDM2A to the cytoplasm (Figures 2A and 2B). Remarkably, the expression of the exogenous p.Pro235Leu variant also caused the cytoplasmic mislocalization of endogenous KDM2A (Figures 2A and 2C). To further illustrate the disruption in the localization of KDM2A variants, nucleocytoplasmic fractionation was performed in HEK293T cells expressing exogenous WT or mutant KDM2A, and both exogenous and endogenous KDM2A were probed using western blotting (Figure 2D). Interestingly, we observed a significant decrease in the nuclear/cytoplasmic ratio in both exogenous and endogenous KDM2A in the p.Pro235Leu variant. In contrast, no such alterations were detected in cells expressing p.Tyr141Cys or WT (Figures 2E and 2F), indicating a LoF in nuclear import for p.Pro235Leu.

Given the altered distribution of both exogenously expressed and endogenous KDM2A caused by p.Pro235Leu, we next sought to assess the impact of these variants on KDM2A levels. Specifically, we introduced WT, p.Pro235Leu, and p.Tyr141Cys plasmids into HEK cells and examined both exogenous and endogenous KDM2A through western blotting (Figure 2G). In the WT and p.Tyr141Cys conditions, there was no notable change in the expression of endogenous *KDM2A*. However, the p.Pro235Leu variant significantly decreased the levels of both exogenously expressed and endogenous KDM2A (Figures 2G–2I). This suggests that the p.Pro235Leu variants likely disrupt the stability of KDM2A.

**Figure 3:**
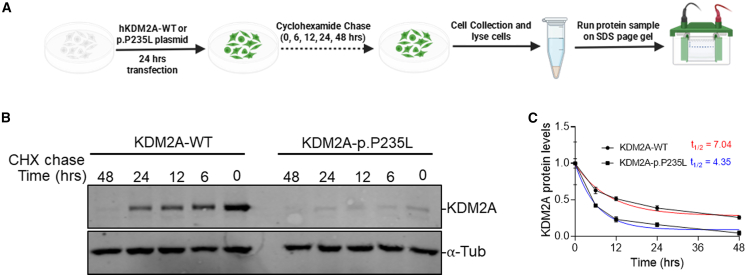
The p.P235L variant alters the stability of the KDM2A (A) Schematic of the cycloheximide (CHX) experiment conducted in HEK293T cells. (B) Representative western blot showing KDM2A levels (anti-HA) in HEK293T cells transfected with either WT or P235L KDM2A plasmid at 0, 6, 12, 24, and 48 h following CHX addition. Tubulin was used as a normalization control. (C) Quantification of the degradation rate and half-life (t_1/2_) of KDM2A after CHX treatment revealed an accelerated depletion of KDM2A in P235L-expressing cells compared to exogenous WT expression (nonlinear regression: one-phase decay, *n* = 3).

To investigate the mechanisms underlying the reduced KDM2A levels, we compared the stability of KDM2A between the WT and p.Pro235Leu mutant protein. We performed WB analysis on HEK cells transfected with either the WT or p.Pro235Leu plasmid. Protein lysates were harvested at 0, 6, 12, 24, and 48 h after CHX treatment (Figures 3A and 3B). The WB analysis revealed that KDM2A is less stable in the p.Pro235Leu variant-expressing cells, with a half-life (t_1/2_) of 4.35 h compared to a t_1/2_ of 7.04 h in WT-expressing cells. This suggests that the differential reduction of KDM2A in p.Pro235Leu variants is due to differences in the stability of KDM2A.

### *KDM2A* variants cause eye degeneration, motor defects, and reduce lifespan in a *Drosophila* model

**Figure 4:**
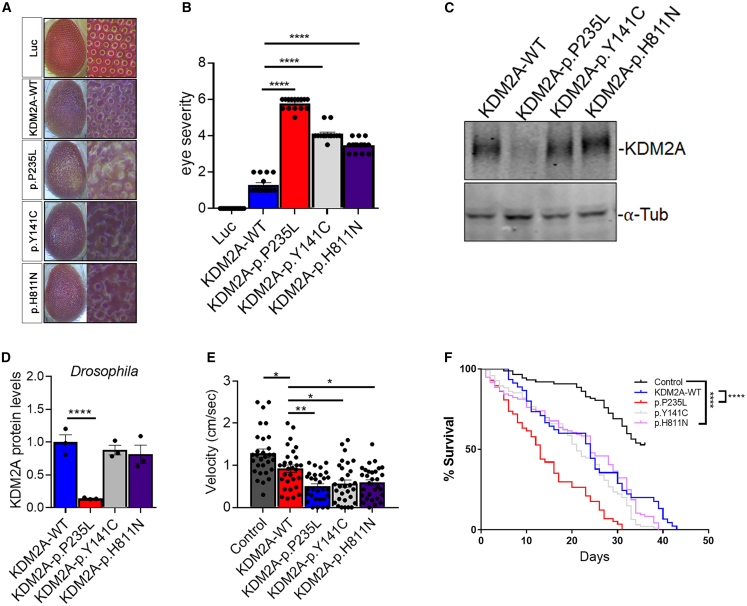
A *Drosophila* model expressing *KDM2A* variants displays differential toxicity *in vivo* (A) Representative panel of adult Drosophila eyes expressing KDM2A-WT, P235L, Y141C, H811N, or luciferase (luc) control. (B) Quantification of eye degeneration severity demonstrated that KDM2A variants significantly enhance toxicity as compared to wild-type KDM2A (^∗∗∗∗^*p* < 0.0001, *n* = 15–20). (C) Western blots of *Drosophila* expressing KDM2A (WT, P235L, Y141C, and H811N) in the eye (GMR-gal4) stained with anti-KDM2A and anti-tubulin. (D) Western blot quantification of KDM2A in *Drosophila* showed that the P235L, but not the Y141C or H811N, variants had a significantly reduced protein level compared to KDM2A-WT (^∗∗∗∗^*p* < 0.0001, *n* = 3). (E) Quantification of climbing velocity (cm/s) in *Drosophila* expressing KDM2A-WT, P235L, Y141C, and H811N pan-neuronally (Elav-gal4) compared to luc control or wild-type KDM2A (*n* = 3 trials, 10 animals per trial, ^∗∗^*p* < 0.01 and ^∗^*p* < 0.05). (F) Kaplan-Meier survival curve of *Drosophila* expressing KDM2A-WT, P235L, Y141C, and H811N in neurons when each is individually compared with luc control (*n* = 50–80, ^∗∗∗∗^*p* < 0.0001). One-way ANOVA was performed in (B)–(D), while log rank with Grehan-Breslow-Wilcoxon tests were performed to determine the significance in (F). All quantifications represent the mean ± SEM.

We next investigated the *in vivo* effects of missense variants in the *Drosophila* model system. To achieve this, we generated transgenic *Drosophila* expressing human *KDM2A*-WT and missense variants (p.Pro235Leu, p.Tyr141Cys, and p.His811Asn) through PhiC31 integrase-mediated site-specific insertion of a single copy of human *KDM2A*. The expression of these transgenes in *Drosophila* using the eye tissue-targeting GMR-gl4 driver revealed a variant-dependent rough eye phenotype, with the p.Pro235Leu variant exhibiting more pronounced effects (Figures 4A and 4B). The expression of WT showed a very mild phenotype. Likewise, in line with observations from HEK293T cells, the expression of the p.Pro235Leu variant in *Drosophila* eyes resulted in a notable decrease in KDM2A levels (Figures 4C and 4D).

To assess whether ectopic expression of *KDM2A* variants could cause motor deficits, we evaluated locomotion in 20-day-old *Drosophila*. We expressed these variants pan-neuronally with the Elav-gal4 driver. While expression of *KDM2A*-WT mildly but significantly reduced climbing velocity, a more profound decrease in climbing ability was observed in *Drosophila* expressing the *KDM2A* mutants, particularly in the p.Pro235Leu variant (Figure 4E). To further determine the toxic impact of KDM2A, we assessed the effects of *KDM2A* variants on survival. Pan-neuronal expression of WT or mutant *KDM2A* significantly reduced survival compared to the luciferase control. However, the p.Pro235Leu variant led to a significantly shorter lifespan compared to both *KDM2A*-WT and the other variants (Figure 4F), suggesting that the cytoplasmic p.Pro235Leu variant confers a toxic gain of function in addition to loss of nuclear activity.

**Figure 5:**
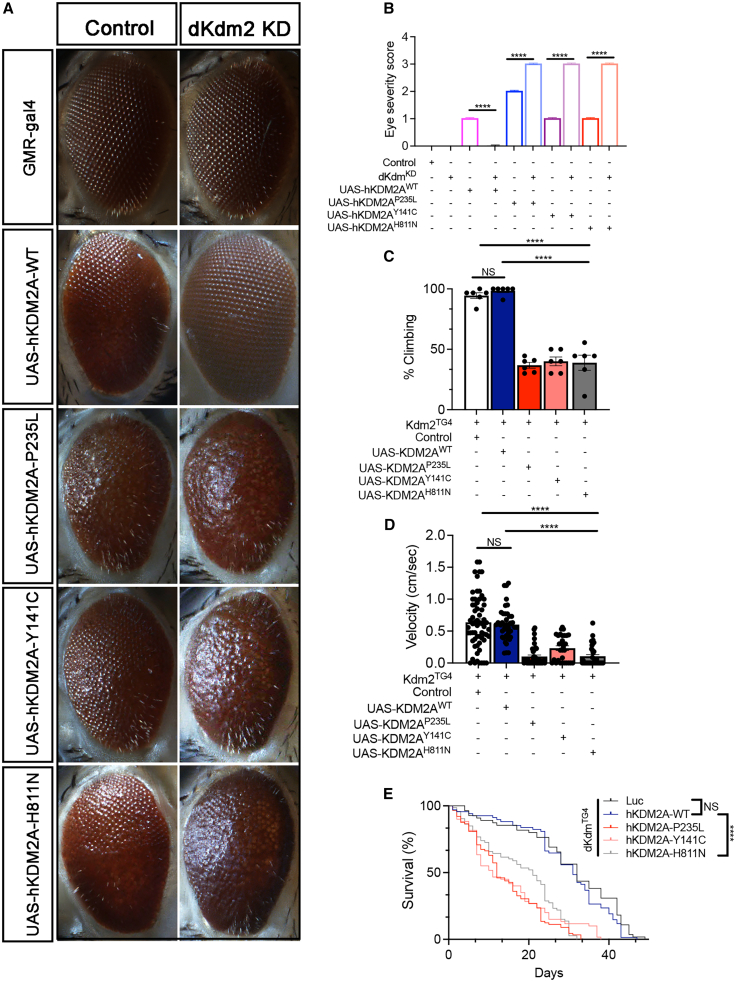
Expression of human *KDM2A* variants in a *Drosophila Kdm2* knockdown or knockout model increases toxicity (A) Images of *Drosophila* eyes expressing human WT or mutant KDM2A in the context of either endogenous Kdm2 or RNAi-mediated Kdm2 knockdown (KD). (B) Quantification shows that human KDM2A (hKDM2A) variants significantly increase eye degeneration severity in the Kdm2 KD background compared to controls with endogenous Kdm2 (^∗∗∗∗^*p* < 0.0001, *n* = 20). Notably, neither hKDM2A-WT with endogenous Kdm2 KD nor Kdm2 KD alone caused overt eye degeneration. (C–E) Quantification analyses revealed a significant reduction in the percentage of *Drosophila* capable of climbing (^∗∗∗∗^*p* < 0.0001, *n* = 6 trials/10 flies per trial) (C), climbing velocity (^∗∗∗∗^*p* < 0.0001, *n* = 3 trials/15–20 flies per trial) (D), and lifespan (Kaplan-Meier survival curve, ^∗∗∗∗^*p* < 0.0001, *n* = 80–90 flies) (E) in Kdm2 KO *Drosophila* expressing hKDM2A variants (P235L, Y141C, and H811N) using the Trojan-Gal4 system, compared to hKDM2A-WT with endogenous Kdm2 KD or KO flies alone. Statistical significance was determined by one-way ANOVA for (B)–(D) and by log rank with Grehan-Breslow-Wilcoxon tests for (E). All data are presented as the mean ± SEM.

Given our data showing that the p.Pro235Leu variant disrupts normal subcellular distribution and potentially impairs the function of endogenous KDM2A, we further investigated the toxicity of *KDM2A* variants in a *Drosophila* model where endogenous *Kdm2* (the single *Drosophila* ortholog of human *KDM2A* and *KDM2B* [MIM: 609078]) is either knocked down or knocked out. First, we knocked down *Kdm2* in *Drosophila* eyes while expressing human WT and mutant (p.Pro235Leu, p.Tyr141Cys, and p.His811Asn) KDM2A. Knockdown (KD) of endogenous *Kdm2* in *Drosophila* eyes did not result in any overt degeneration, and expression of human KDM2A-WT in the Kdm2 KD background produced only a mild phenotype. In contrast, expression of the human *KDM2A* variants (p.Pro235Leu, p.Tyr141Cys, and p.His811Asn)—most prominently p.Pro235Leu—in the *Kdm2* KD background significantly exacerbated the degenerative eye phenotype compared to their respective controls (Figures 5A and 5B). These results confirm that p.Pro235Leu’s effects cannot be explained by the loss of *Kdm2* alone and instead reflect a toxic gain-of-function mechanism.

We further utilized the Trojan-MiMIC Gal4 driver system, which employs RMCE of a Mi{MIC} insertion, resulting in the expression of GAL4 under the control of *Kdm2* regulatory sequences.^27^^,^^34^ Consequently, *Drosophila Kdm2* is knocked out in the Trojan-Gal4Kdm2 lines, allowing for a robust analysis of human *KDM2A* variants in a clean genetic background. We assessed motor function and lifespan in adult *Drosophila* expressing human *KDM2A*-WT and its variants in the specific cell types where endogenous *Drosophila Kdm2* is normally expressed. The lack of phenotype in *Kdm2* knockout (KO) animals suggests that the loss of *Kdm2* in adults is not critical under the conditions tested (Figures 5C and 5D). Similarly, we found that expression of human *KDM2A*-WT did not affect motor function or lifespan compared to Kdm2 KO flies. However, the human *KDM2A* variants (p.Pro235Leu, p.Tyr141Cys, and p.His811Asn) resulted in significantly worse motor function and survival compared to both the KO and the human WT scenarios (Figures 5C–5E). Altogether, these findings suggest that these variants in *KDM2A* are toxic, possibly due to a gain-of-function and/or a LoF mechanism.

### Methylome analysis

**Figure 6:**
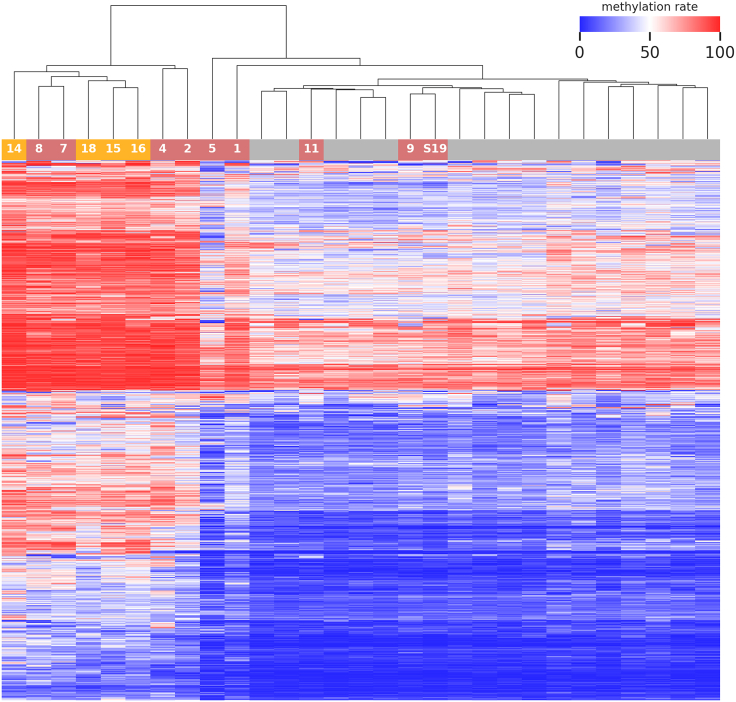
Episignature of pLoF and missense variants in *KDM2A* Heatmap displays hierarchical clustering of selected CpG sites of the episignature. Columns represent probes (gray: control probes; yellow: *KDM2A* pLoF variants; light red: *KDM2A* missense variants; number represents individual in the cohort). Rows represent CpG sites. Color represents methylation, ranging from dark blue (no methylation) to dark red (full methylation). A distinct separation between control samples and those from individuals with variants in *KMD2A* is observed. For individual S19, there is insufficient evidence for the diagnosis of a *KDM2A*-related syndromic neurodevelopmental disorder (a *de novo* origin of the variant could not be proven, and methylation analysis does not support a diagnosis either; see also the supplemental notes and Table S4).

As KDM2A is a component of the epigenetic machinery, it was hypothesized that disruption of KDM2A function by *de novo* variants would cause an abnormal methylation pattern in peripheral blood as a *de facto* functional readout. Analysis of enzymatic-methylation sequencing data of thirteen individuals with pLoF variants and missense variants compared to a control group identified 817 DMRs. After applying stringent filtering criteria (*p*-adj < 0.01 and min. mean methylation difference > 10%), we defined a first episignature of the *KDM2A* cases. This signature includes 598 DMRs (585 hypermethylated and 13 hypomethylated regions) with a median methylation difference of 16.2% and a median length of ∼226 nt/20 CpGs (Table S8). Hierarchical clustering of affected individuals and controls revealed a distinct *KDM2A* cluster with individuals harboring frameshift variants (individuals 14, 15, 16, and 18) and missense variants (individuals 2, 4, 7, and 8) grouping together within this *KDM2A* cluster (Figure 6). Data of four individuals (individuals 1, 5, 9, and 11) with missense variants were shown to group outside the bigger *KDM2A* cluster and within the controls. In addition, the methylation analysis of individual S19, where the *de novo* origin of the missense variant could not be tested in the parents, also showed a methylation result comparable to the control group.

## Discussion

In this study, we provide a detailed description of 18 individuals with heterozygous variants in *KDM2A*, including 17 with confirmed *de novo* status, all presenting with a syndromic NDD.

The affected individuals show an overlapping phenotype of developmental delay/intellectual disability and several recurrent symptoms of note: growth abnormalities (including IUGR and short stature), microcephaly, and feeding difficulties that are present in 35%–72% of individuals. Developmental delay/intellectual disability and growth abnormalities are recurrent symptoms among the Mendelian disorders of the epigenetic machinery,^3^ underscoring that the *KDM2A*-related rare disease delineated here shows a clinical overlap. Many of these disorders also present with a recognizable facial gestalt and include some of the classic dysmorphic syndromes, such as Kabuki syndrome (e.g., *KMT2D*; MIM: 147920 and MIM: 602113) or Rubinstein-Taybi syndrome (e.g., *CREBBP*; MIM: 180849 and MIM: 600140). The affected individuals in the *KDM2A* cohort also showed facial dysmorphism, highlighted by recurrent observations of epicanthus, upslanted palpebral fissures, thin upper and/or lower lips, and low-set ears as potentially defining features (Figure 1B). Deep phenotyping of facial images of affected individuals with the help of facial recognition tools could help to detect this potential typical facial gestalt of the *KDM2A*-related syndromic NDD in routine clinical genetic care.^35^^,^^36^ An MCD, such as the polymicrogyria observed in individual 4, is not a phenotype seen in other disorders of the epigenetic machinery. The trio exome analysis of this individual did not reveal any additional causative variant(s) for this phenotype in genes associated with an MCD nor additional variants in other candidate genes.^37^ It remains, therefore, unclear whether polymicrogyria is part of the phenotypic spectrum of the *KDM2A*-related disorder or if another, undetected genetic cause is involved. Of note, feeding difficulties were only present in individuals with *de novo* missense variants in *KDM2A* and were absent in individuals with pLoF variants, suggesting a potential genotype-phenotype correlation. Similarly, microcephaly was observed exclusively in individuals with missense variants, with the exception of the prenatal subject carrying a pLoF variant. Other recurrent phenotypes, however, did not show any potential genotype-phenotype correlations, as they were observed in individuals who harbor missense variants and individuals with pLoF variants.

As mentioned previously, *KDM2A* is a gene with a significantly reduced number of pLoF and missense variants. Among KDM genes, *KDM2A* is in fact the most highly constrained gene with respect to both missense and pLOF variants (see constraint score landscape of KDMs in Figure S2 as well as Table S6). This high constraint compared to genes within the group of KDMs, where variants are known to cause a rare disease, strengthens our case that rare variants in *KDM2A* are also causative for a Mendelian condition.

It is now also possible to evaluate prior assessments on what phenotypes could be caused by variants in candidate genes such as *KDM2A*. In a recent work by Dhindsa et al., using a machine learning approach based on gene constraint, expression, and many other gene-level annotations, *KDM2A* was predicted to cause an autosomal-dominant phenotype of developmental delay, developmental epileptic encephalopathy, and autism in the 99.5th percentile or higher for each phenotype individually.^38^ This prior assessment aligns with our phenotypic findings, including that both autism and epilepsy are important parts of the phenotypic spectrum of the *KDM2A*-related syndromic disorder, although only five affected individuals presented with seizures, and four had a diagnosis of autism.

The identified *de novo* missense variants in *KDM2A* are located in or around the JmjC domain, essential for demethylation activity, and the CxxC domain (a zinc finger [ZF]), essential for DNA binding.^1^^,^^39^ In *KDM2B*, *de novo* missense variants are also preferably located in or around these two domains specifically (Figure S3).

In this study, we investigated the consequences of missense variants in *KDM2A* on the subcellular distribution of KDM2A and toxicity. Previous studies demonstrated that KDM2A primarily localizes to the nucleus, where it binds to unmethylated CpG DNA via the ZF-CxxC domain.^32^ Consistent with these findings, *KDM2A*-WT expressed in HEK293T cells exhibited predominantly nuclear localization. In contrast, our observations revealed distinct localization patterns for the p.Tyr141Cys and p.His811Asn variants, which displayed primarily nuclear with occasional cytoplasmic aggregation, while the p.Pro235Leu variant showed significant nuclear exclusion and cytoplasmic accumulation (Figure 2). Moreover, the endogenously expressed KDM2A undergoes cytoplasmic redistribution in the presence of the exogenously expressed p.Pro235Leu variant. This observation was further supported by nuclear-cytoplasmic fractionation experiments, which consistently demonstrated predominant cytoplasmic localization of the p.Pro235Leu variant, suggesting a potential disruption in nuclear import. The loss of nuclear KDM2A may impair its ability to repress the transcription of centromeric satellite repeats and maintaining a heterochromatic state.^33^ Additionally, nuclear redistribution of KDM2A to the cytoplasm may also impair its ability to bind and regulate the stability of non-phosphorylated nuclear β-catenin, thereby potentially affecting the Wnt/β-catenin signaling pathway.^40^ Alternatively, cytoplasmic accumulation of KDM2A may confer a toxic gain of function. However, further investigations are required to elucidate the precise underlying mechanism. Moreover, our data revealed that the forced expression of the p.Pro235Leu variant is accompanied by a significant reduction in both endogenous and exogenously expressed KDM2A levels, indicating compromised protein stability. This was confirmed through CHX chase experiments, which showed that the p.Pro235Leu variant reduces the t_1/2_ of KDM2A, suggesting that this variant negatively impacts protein stability. Given its location within the JmjC catalytic domain, which is critical for KDM2A’s histone demethylase activity, the p.Pro235Leu substitution may alter enzymatic function, protein stability, or cofactor binding. This is consistent with our experimental observations of altered protein stability and mislocalization.

In addition to markedly reducing protein stability and its location within *KDM2A*’s JmjC catalytic domain, the p.Pro235Leu substitution likely perturbs the local folding of the catalytic core in a manner that secondarily disrupts chromatin engagement and nuclear retention. Structural and functional studies indicate that the JmjC module is upstream of KDM2A’s chromatin-targeting ZF-CxxC domain and that interdomain integrity is important for high-affinity CpG binding and stable nuclear association.^33^^,^^41^^,^^42^ We therefore infer that substitution of the rigid proline at 235 with a more flexible hydrophobic leucine destabilizes the JmjC fold, producing a conformational change that probably (1) weakens cooperative interactions with the ZF-CxxC chromatin anchor, (2) exposes sequences that favors cytoplasmic routing (for example, cryptic nuclear export signals or degron motifs), or (3) increases susceptibility to post-translational modifications that promote ubiquitin-dependent nuclear export and clearance. By contrast, p.Tyr141Cys resides in the JmjN subdomain of the catalytic module, and p.His811Asn maps to the C-terminal F box region; neither substitution directly perturbs the ZF-CxxC chromatin-binding fold or characterized nuclear localization elements, which plausibly explains why these variants largely retain nuclear localization and do not recapitulate the mislocalization observed for p.Pro235Leu. Thus, the striking cytoplasmic mislocalization of p.Pro235Leu most likely reflects a domain-specific structural perturbation of the catalytic core that compromises KDM2A’s chromatin tethering and nuclear retention rather than a global unfolding of the protein, highlighting a mechanistic distinction between disruptions of KDM2A’s enzymatic architecture versus its chromatin-targeting module.

The observed redistribution of KDM2A from the nucleus to the cytoplasm may exert toxic effects on cells. Similar mechanisms have been reported in amyotrophic lateral sclerosis and frontotemporal dementia (ALS/FTD), where pathogenic variants in proteins such as FUS and TDP-43 lead to nuclear clearance, contributing to cellular toxicity in various animal models, including *Drosophila*.^23^^,^^43^ To test whether *KDM2A* variants induce toxicity in an *in vivo* system, we generated *Drosophila* models expressing three missense variants (p.Tyr141Cys, p.Pro235Leu, and p.His811Asn). Ectopic expression of *KDM2A* in neuronal cells led to eye degeneration, motor deficits, and reduced survival (Figure 2), with the p.Pro235Leu variant exhibiting the highest toxicity, consistent with our *in vitro* findings. While *KDM2A*-WT caused mild toxicity, this parallels observations from FUS expression studies in *Drosophila*.^23^ Our data suggest that both loss of nuclear function and a potential gain-of-function effect in the cytoplasm contribute to the observed cellular toxicity, particularly evident in the p.Pro235Leu variant. Further research is needed to examine the exact mechanisms underlying *KDM2A* variant-mediated toxicity. Our *in vivo Drosophila* data using a *Kdm* KO or KD system suggested a possible gain of function of *KDM2A* variants. Our data showed that the expression of human WT *KDM2A* in the KD or KO background did not affect eye severity, motor function, or lifespan, which were similar to those observed in KD or KO *Kdm* flies. In contrast, expression of *KDM2A* variants resulted in significantly worse phenotypes compared to both KO flies and those expressing human *KDM2A*-WT. These findings suggest that variants in *KDM2A* may confer a new or enhanced toxic effect, characteristic of gain-of-function variants. However, a limitation of our study is the absence of direct functional analysis of other reported *KDM2A* variants, particularly the nonsense variants p.Arg20^∗^ (c.58C>T) and p.Tyr193^∗^ (c.579C>G). These premature stop codons likely result in truncated proteins lacking critical functional domains, such as the JmjC catalytic domain and the CxxC ZF, potentially leading to complete loss of function through nonsense-mediated mRNA decay or dominant-negative effects. While our mechanistic investigation focused on the p.Pro235Leu missense variant, which we show alters protein stability, localization, and potentially chromatin interactions, evaluating the functional consequences of the truncating variants will be essential to determine whether they act through similar or distinct pathogenic pathways. Future studies incorporating these variants will provide a more comprehensive understanding of *KDM2A*-related disease mechanisms.

We analyzed blood-derived DNA and detected a *KDM2A*-related episignature, further underscoring a shared pathomechanism among affected individuals with missense and pLoF variants. In contrast, methylation analysis of several individuals with missense variants showed results comparable to controls (individuals 1, 5, 9, and 11). All four variants are of *de novo* origin, the clinical phenotype of all individuals fits the described spectrum, and functional analysis of, e.g., the missense variant of individual 11 (p.His811Asn) showed a toxic effect in the *Drosophila* model. Thus, the diagnosis of a *KDM2A*-related syndromic NDD is warranted in all four individuals. This reinforces the point that the lack of an aberrant methylation profile does not rule out a specific diagnosis, as laid out in a recent clinical utility recommendation for episignature testing.^44^ However, in the case of individual S19, where (1) the *de novo* origin of the underlying missense variant could not be proven due to the individuals’ adoption at age 2 months and (2) a lack of a *KDM2A*-associated methylation pattern, a diagnosis of a *KDM2A*-related NDD is not warranted, and thus, we did not include this individual in the main cohort (see supplemental notes and Table S4). In the future, with further refinement, this episignature could serve as a valuable tool for interpreting variants of unknown significance and aid in the diagnosis of affected individuals.

In summary, we use different lines of clinical, genetic, functional, and epigenetic evidence to firmly establish *de novo* variants in *KDM2A* as the cause of a syndromic NDD.

## Data and code availability

All identified variants in *KDM2A* have been uploaded to ClinVar with the following accession numbers: VCV003778797, VCV003778794, VCV003778798, VCV003778805, VCV003377192, VCV003778811, VCV003778812, VCV003778799, VCV003778800, VCV003778813, VCV003359230, VCV003778814, VCV003778815, VCV003778795, VCV003778801, VCV003778796, VCV003778802, and VCV003778803 (https://www.ncbi.nlm.nih.gov/clinvar/submitters/506086/).

Pipeline for enzymatic-methylation sequencing data processing is available at https://github.com/StephanHolgerD/methylmappdedupmethyldackelsnakemake.

## Consortia

The members of the Undiagnosed Diseases Network are Maria T. Acosta, David R. Adams, Ben Afzali, Ali Al-Beshri, Eric Allenspach, Aimee Allworth, Raquel L. Alvarez, Justin Alvey, Ashley Andrews, Euan A. Ashley, Carlos A. Bacino, Guney Bademci, Ashok Balasubramanyam, Dustin Baldridge, Jim Bale, Michael Bamshad, Deborah Barbouth, Pinar Bayrak-Toydemir, Anita Beck, Alan H. Beggs, Edward Behrens, Gill Bejerano, Hugo J. Bellen, Jimmy Bennett, Jonathan A. Bernstein, Gerard T. Berry, Anna Bican, Stephanie Bivona, Elizabeth Blue, John Bohnsack, Devon Bonner, Nicholas Borja, Lorenzo Botto, Lauren C. Briere, Elizabeth A. Burke, Lindsay C. Burrage, Manish J. Butte, Peter Byers, William E. Byrd, Kaitlin Callaway, John Carey, George Carvalho, Thomas Cassini, Sirisak Chanprasert, Hsiao-Tuan Chao, Ivan Chinn, Gary D. Clark, Terra R. Coakley, Laurel A. Cobban, Joy D. Cogan, Matthew Coggins, F. Sessions Cole, Brian Corner, Rosario I. Corona, William J. Craigen, Andrew B. Crouse, Vishnu Cuddapah, Precilla D’Souza, Hongzheng Dai, Kahlen Darr, Surendra Dasari, Joie Davis, Margaret Delgado, Esteban C. Dell'Angelica, Katrina Dipple, Daniel Doherty, Naghmeh Dorrani, Jessica Douglas, Emilie D. Douine, Dawn Earl, Lisa T. Emrick, Christine M. Eng, Cecilia Esteves, Kimberly Ezell, Elizabeth L. Fieg, Paul G. Fisher, Brent L. Fogel, Jiayu Fu, William A. Gahl, Rebecca Ganetzky, Emily Glanton, Ian Glass, Page C. Goddard, Joanna M. Gonzalez, Andrea Gropman, Meghan C. Halley, Rizwan Hamid, Neal Hanchard, Kelly Hassey, Nichole Hayes, Frances High, Anne Hing, Fuki M. Hisama, Ingrid A. Holm, Jason Hom, Martha Horike-Pyne, Alden Huang, Yan Huang, Anna Hurst, Wendy Introne, Gail P. Jarvik, Suman Jayadev, Orpa Jean-Marie, Vaidehi Jobanputra, Oguz Kanca, Yigit Karasozen, Shamika Ketkar, Dana Kiley, Gonench Kilich, Eric Klee, Shilpa N. Kobren, Isaac S. Kohane, Jennefer N. Kohler, Bruce Korf, Susan Korrick, Deborah Krakow, Elijah Kravets, Seema R. Lalani, Christina Lam, Brendan C. Lanpher, Ian R. Lanza, Kumarie Latchman, Kimberly LeBlanc, Brendan H. Lee, Kathleen A. Leppig, Richard A. Lewis, Pengfei Liu, Nicola Longo, Joseph Loscalzo, Richard L. Maas, Ellen F. Macnamara, Calum A. MacRae, Valerie V. Maduro, AudreyStephannie Maghiro, Rachel Mahoney, May Christine V. Malicdan, Rong Mao, Ronit Marom, Gabor Marth, Beth A. Martin, Martin G. Martin, Julian A. Martínez-Agosto, Shruti Marwaha, Allyn McConkie-Rosell, Ashley McMinn, Matthew Might, Mohamad Mikati, Danny Miller, Ghayda Mirzaa, Breanna Mitchell, Paolo Moretti, Marie Morimoto, John J. Mulvihill, Lindsay Mulvihill, Mariko Nakano-Okuno, Stanley F. Nelson, Serena Neumann, Dargie Nitsuh, Donna Novacic, Devin Oglesbee, James P. Orengo, Laura Pace, Stephen Pak, J. Carl Pallais, Neil H. Parker, LéShon Peart, Leoyklang Petcharet, John A. Phillips III, Filippo Pinto e Vairo, Jennifer E. Posey, Lorraine Potocki, Barbara N. Pusey Swerdzewski, Aaron Quinlan, Daniel J. Rader, Ramakrishnan Rajagopalan, Deepak A. Rao, Anna Raper, Wendy Raskind, Adriana Rebelo, Chloe M. Reuter, Lynette Rives, Lance H. Rodan, Martin Rodriguez, Jill A. Rosenfeld, Elizabeth Rosenthal, Francis Rossignol, Maura Ruzhnikov, Marla Sabaii, Jacinda B. Sampson, Timothy Schedl, Lisa Schimmenti, Kelly Schoch, Daryl A. Scott, Elaine Seto, Vandana Shashi, Emily Shelkowitz, Sam Sheppeard, Jimann Shin, Edwin K. Silverman, Giorgio Sirugo, Kathy Sisco, Tammi Skelton, Cara Skraban, Carson A. Smith, Kevin S. Smith, Lilianna Solnica-Krezel, Ben Solomon, Rebecca C. Spillmann, Andrew Stergachis, Joan M. Stoler, Kathleen Sullivan, Shamil R. Sunyaev, Shirley Sutton, David A. Sweetser, Virginia Sybert, Holly K. Tabor, Queenie Tan, Arjun Tarakad, Herman Taylor, Mustafa Tekin, Willa Thorson, Cynthia J. Tifft, Camilo Toro, Alyssa A. Tran, Rachel A. Ungar, Adeline Vanderver, Matt Velinder, Dave Viskochil, Tiphanie P. Vogel, Colleen E. Wahl, Melissa Walker, Nicole M. Walley, Jennifer Wambach, Michael F. Wangler, Patricia A. Ward, Daniel Wegner, Monika Weisz Hubshman, Mark Wener, Tara Wenger, Monte Westerfield, Matthew T. Wheeler, Jordan Whitlock, Lynne A. Wolfe, Heidi Wood, Kim Worley, Shinya Yamamoto, Zhe Zhang, and Stephan Zuchner.

## Acknowledgments

We thank all families who participated in this study and generously contributed their time and data. S.S. is funded through the Albert Rowe II Endowed Chair in Genetics. The research reported in this manuscript was in part supported by the 10.13039/100000002NIH Common Fund, through the Office of Strategic Coordination/Office of the NIH Director and the National Institute of Neurological Disorders and Stroke of the NIH under award numbers U01HG010218 and U01HG007708. The content is solely the responsibility of the authors and does not necessarily represent the official views of the NIH.

## Declaration of interests

D.A.C., L.M.D., and S.V.M. are employees of and may own stock in GeneDx, LLC.

## Web resources

GenBank, https://www.ncbi.nlm.nih.gov/genbank/
GeneMatcher, https://genematcher.org/
gnomAD, https://gnomad.broadinstitute.org/
OMIM, https://www.omim.org/

## Supplemental information

Document S1. Figures S1–S3, Tables S1 and S2, and supplemental notes

Data S1. Tables S3–S8

Document S2. Article plus supplemental information
