# Supplementary material for: De novo variants in KDM2A cause a syndromic neurodevelopmental disorder
Source: Am J Hum Genet. 2025 Dec 29;113(1):100–16. doi: 10.1016/j.ajhg.2025.12.004 (PMC12824617; doi:10.1016/j.ajhg.2025.12.004)
Supplement: Document S1. Figures S1–S3, Tables S1 and S2, and supplemental notes [file mmc1.pdf]

The American Journal of Human Genetics, Volume *113*

Supplemental information

De novo variants in *KDM2A* cause a syndromic neurodevelopmental disorder

Eric N. Anderson, Stephan Drukewitz, Sukhleen Kour, Anuradha V. Chimata, Deepa S. Rajan, Senta Schönnagel, Karen L. Stals, Deirdre Donnelly, Siobhan O'Sullivan, John F. Mantovani, Tiong Y. Tan, Zornitza Stark, Pia Zacher, Nicolas Chatron, Pauline Monin, Severine Drunat, Yoann Vial, Xenia Latypova, Jonathan Levy, Alain Verloes, Jennefer N. Carter, Devon E. Bonner, Suma P. Shankar, Jonathan A. Bernstein, Julie S. Cohen, Anne Comi, Deanna Alexis Carere, Lisa M. Dyer, Sureni V. Mullegama, Pedro A. Sanchez-Lara, Katheryn Grand, Hyung-Goo Kim, Afif Ben-Mahmoud, Sidney M. Gospe Jr., Rebecca S. Belles, Gary Bellus, Klaske D. Lichtenbelt, Renske Oegema, Anita Rauch, Ivan Ivanovski, Frederic Tran Mau-Them, Aurore Garde, Rachel Rabin, John Pappas, Annette E. Bley, Janna Bredow, Timo Wagner, Eva Decker, Carsten Bergmann, Louis Domenach, Henri Margot, Undiagnosed Diseases Network, Johannes R. Lemke, Rami Abou Jamra, Julia Hentschel, Heather Mefford, Amit Singh, Udai Bhan Pandey, and Konrad Platzer

Table of contents

Supplemental Note: Case reports of individuals with causative variants in *KDM2A*

Individual 1, c.422A>G, p.(Tyr141Cys), *de novo*

Individual 2, c.704C>T, p.(Pro235Leu), *de novo*

Individual 3, c.850C>T, p.(His284Tyr), *de novo*

Individual 4, c.956G>A, p.(Arg319Gln), *de novo*

Individual 5, c.1571T>G, p.(Phe524Cys), *de novo*

Individual 6, c.1703G>A, (p.Arg568Gln), *de novo*

Individual 7, c.1772T>C, p.(Met591Thr), *de novo*

Individual 8, c.1796G>C, p.(Arg599Pro), *de novo*

Individual 9, c.2327A>G, p.(Lys776Arg), *de novo*

Individual 10, c.2328G>T, p.(Lys776Asn), *de novo*

Individual 11, c.2431C>A, p.(His811Asn), *de novo*

Individual 12, c.58C>T, p.(Arg20*), *de novo*

Individual 13, c.579C>G, p.(Tyr193*), *de novo*

Individual 14, c.1676dup, p.(Ile560Aspfs*71), *de novo*

Individual 15, c.1677delG, p.(Ile560Leufs*32), *de novo*

Individual 16, c.2404dup, p.(Thr802Asnfs*49), heterozygous

Individual 17, c.2667delC, p.(Asp889Glufs*47), *de novo*

Individual 18, c.2809_2812dup, p.(Cys938*), *de novo*

Supplemental Note: Case report of an individual with a variant in *KDM2A* but insufficient evidence for causality

Individual S19, c.2323G>A, p.(Glu775Lys), heterozygous

Figure S1. Pedigrees of all families

Figure S2. Constraint score landscape of KDM genes.

Figure S3. Overview of domain structure and reported variants in the literature in KDM genes.

Table S1. Variant information and classification according to ACMG criteria.

Table S2. *In silico* prediction of missense variants in *KDM2A*.

Table S3. Detailed clinical data of individuals with causative variants in *KDM2A*

Table S4. Detailed clinical data of an individual with variant in *KDM2A* but insufficient evidence for causality

Table S5. Annotation and *in silico* scores of all missense variants in *KDM2A*

Table S6. List of KDM genes

Table S7. Reported variants in the literature in KDM genes

Table S8. Methylation data of the *KDM2A*-related Episignature

References

Supplemental Note: Case reports of individuals with causative variants in *KDM2A*

Individual 1, c.422A>G, p.(Tyr141Cys), *de novo*

This child is the third child of non-consanguineous parents with her older brother and sister being completely fit and well. There is no family history of note. She presented at 8 months of age with poor feeding and weight gain, with her weight falling through the centiles. She also had developmental delay. At 3 years and 8 months, she was speaking in short sentences with fairly good comprehension. She has feeding difficulties and eats mainly pureed foods. She is walking independently but prefers to furniture walk. She has poor balance and coordination. Due to low tone, she has splints on both feet. She is showing some signs of autistic behavior, such as lining up toys and displaying repetitive play. She wears glasses (+1.50DS / -0.25DC @95 in the right eye and a +2.00DS / -0.25DC @80 in the left). She has central hypothyroidism, diagnosed at around 1 year of age. She has dysmorphic features including midface hypoplasia, epicanthic folds, widely spaced and deeply set eyes, low-set and posteriorly rotated ears, saggy cheeks and a thin vermilion of the upper lip.

Individual 2, c.704C>T, p.(Pro235Leu), *de novo*

This 8-year-old boy was the first child of a non-consanguineous couple from the USA. He was born at term weighing 3.4 kg (43rd centile) to a prima gravida 28-year-old mother following a pregnancy complicated by recurrent vaginal bleeding requiring treatment with progesterone and bedrest. He had an unremarkable newborn course except for limited lip and tongue mobility which required a change from breast to bottle-feeding. Parents had concerns for his social indifference, lack of eye contact and absence of reciprocal smiling by 6 weeks of age. He began exhibiting repetitive mannerisms in the first year of life including hand-flapping with excitement, covering his ears, staring at lights, and preferring to hold red objects which he carried with him compulsively. His emotional regulation was poor including periods of whining and crying for no apparent reason for at least half of every day alternating with periods of appearing to be self-absorbed and contented. He was diagnosed with global developmental delay and autism spectrum disorder at 17 months of age. He has not developed speech or gestural communication and communicates his needs by sporadic use of an augmentative communication device and whining or crying. He has had normal brain MRI and biochemical testing including carbohydrate deficient transferrin electrophoresis, plasma amino acids, urine organic acids and mucopolysaccharides, and lactic and pyruvic acid levels.

He was diagnosed with gluten sensitivity on borderline positive autoantibody screening at age 6 years and immune deficiency with reduced levels of IgA and pediatric autoimmune neuropsychiatric disorder associated with streptococcal infection (PANDAS) with elevated ASO titers at age 7 years when he developed symptoms of reduced oral intake, tics, “rage attacks” and self-injurious behaviors without provocation. He has been treated with Intravenous Immunoglobulin (IVIG) with only minimal improvement in tantrums and compulsive behaviors. He has not had seizures or developmental regression.

His examination is positive for height at the 14th centile, 57th centile for weight and 70th centile for OFC, a small chin, prominent vermilion of the upper lip, and slightly enlarged ears.

Individual 3, c.850C>T, p.(His284Tyr), *de novo*

This boy is the only child to his healthy unrelated parents of Anglo-Celtic heritage of average height. He was born by vaginal delivery at term after an uncomplicated pregnancy. He needed brief stimulation and CPAP at birth, but this was only required for less than 24 hours. He was admitted to Special Care Nursery for treatment with IV antibiotics for suspected sepsis because of maternal Group B strep positivity. He had feeding difficulties and slow weight gain. Although his feeding difficulties resolved, he remains a fussy eater and growth restricted.

He started walking at 14 months but concerns were raised about his speech development when his first words were at 2 years. His fine motor skills were also delayed, but social skills were normal. He has always been very active and was assessed with ADHD.

He was first referred to genetics at age 6 years of age. He was noted to be growth restricted with all parameters below the 3rd centile and was of lean build without evidence of lipodystrophy. He had triangular facial features but was otherwise eumorphic. There was a small pigmented macule on his back but no congenital malformations noted on examination.

Individual 4, c.956G>A, p.(Arg319Gln), *de novo*

The 25-year-old female is the eldest daughter of non-consanguineous parents from Afghanistan. Additionally to having three healthy siblings, her mother suffered two miscarriages of unknown cause. The pregnancy is said to have been "normal". When being a newborn, she was treated for newborn jaundice by phototherapy. The developmental delay was noticed shortly after birth, but the family was unable to provide detailed information regarding developmental milestones. She is said to have learned to walk at two years of age and has never been able to speak. She does understand Persian and communicates non-verbally very skillfully. Her family believes that the problems of speaking are caused by her tongue and possibly describe the clinical presentation of dysarthria.

The epilepsy syndrome with focal onset seizures (aura and focal to bilateral tonic clonic) are said to have started around the age of ten, years before the family left for Germany. It is believed that she feels an epileptic aura, since she always tries to seek contact. After she stares absently and salivates profusely before generalization into a tonic-clonic seizure. On interictal EEG, biparietal focal epileptiform discharges were observed. The brain MRI showed signs of disturbed neuronal migration and organization of cortex in form of changes out of the lissencephaly spectrum (cobblestone malformation/polymicrogyria with anterior maximum, diffuse gliosis, heterotopia, simple ventricular structure). The speech impairment may potentially be caused by a perisylvian syndrome, when taking these brain MRI findings into account. The pharmacoresistant epilepsy was previously treated with valproate and carbamazepine. At present she was treated with levetiracetam and oxcarbamazepine. Upon clinical examination she exhibits upslanted palpebral fissures, a narrow mouth with a thin upper and lower vermilion of the lip, facial asymmetry (after a nasal fracture), a small stature (149 cm, - 3 SD) and conical tapered fingers.

Individual 5, c.1571T>G, p.(Phe524Cys), *de novo*

She is the first child of a non-consanguineous family and has a younger sister. Her mother underwent surgical removal of a cavernoma, and her cousin had a child with an undiagnosed motor handicap. Her niece has been diagnosed with an autism spectrum disorder. She was born at 38 weeks of gestation following a pregnancy marked by intrauterine growth restriction (IUGR). At birth, her weight was 2480 g (8th centile), length 44 cm (1st centile), and OFC 31 cm (2nd centile). During the neonatal period, she experienced feeding difficulties and jaundice. In her first year, she had several seizure episodes associated with fever and required treatment with valproate. She was subsequently tapered off but a recurrence of seizures at age nine required treatment of valproate again. Treatment was then stopped at age 12 years and she remained seizure free since then. Regarding her motor development, she achieved sitting between 12 and 18 months and began walking at 21 months. She started using her first words at 12 months. Initially, she attended a regular school with assistance until the second year of preschool, after which she transitioned to a specialized class. Her OFC growth trajectory ranged between -3 and -4 SD.

At her latest clinical examination at the age of 13, she attended a specialized class for children with difficulties. She struggled to acquire reading skills but could recognize letters. Her healthcare plan included weekly speech therapy sessions and ongoing psychological follow-up. Her weight was 32.5 kg (-3.0 SD), height 153 cm (-0.8 SD), and OFC at 50 cm (-3.5 SD). Clinically, she presented with a triangular face, and a pointed chin. Divergent strabismus was observed. She had arachnodactyly. In the neuropsychological evaluation, her intellectual functioning was heterogeneous, with mild impairment in cognitive functioning and attention deficit disorder. Her verbal comprehension index was 73, visuospatial index 78, fluid reasoning 64, working memory 59, and processing speed 45. Both her EEG and brain MRI showed no abnormal findings. Overall, she exhibited pre- and postnatal microcephaly, developmental delay, and dysmorphic features.

This individual passed away at the age of 14 years due to acute respiratory distress syndrome in the context of a complicated flu.

Individual 6, c.1703G>A, (p.Arg568Gln), *de novo*

This boy is the 2nd child of an unrelated couple. The family history is unremarkable. Pregnancy was characterized by intrauterine growth retardation beginning at 34 weeks of gestation associated with pathological Doppler measurements. He was born at 37 weeks + 2 days of gestation by cesarean section. His birth weight was 1900 g (6th centile), with a height of 45.5 cm (40th centile) and an OFC of 32 cm (25th centile). During the neonatal period, he required enteral feeding for 2 weeks and was hospitalized for 1 month. Since then, he has had persistent feeding difficulties, with severe gastroesophageal reflux. Morphologically, he has a rounded forehead and bilateral epicanthus. Regarding his motor development, he achieved sitting at 1 year and started walking at 18 months. He said his first words at 20 months. He has undergone surgery for cryptorchidism. He has severe learning disabilities, with a significant language impairment associated with an attention deficit disorder and behavioral problems. He attends a regular school with assistance. He has weekly speech therapy and orthoptic sessions and receives psychological support. Growth hormone treatment has been initiated to address the stagnation in his growth. Under treatment, his weight and stature trajectories are ranging between -3 and -5.5 SD. A brain MRI showed no abnormal findings.

Individual 7, c.1772T>C, p.(Met591Thr), *de novo*

The individual was born at 41 weeks via vaginal delivery to a 30-year-old G2P1-2 mother and a 33-year-old father. The pregnancy was notable for maternal GERD and transient polyhydramnios. There were no prenatal exposures. She was born small for gestational age. Birth weight was 2920 g (17th centile, -0.96 SD), birth length was 47 cm (16th centile, -0.98 SD), and birth head circumference was 35 cm (57th centile, 0.18 SD). Apgar scores were 8 and 8 at 1 minute and 5 minutes respectively. Suctioning and brief oxygen was required after delivery. The neonatal period was complicated by jaundice treated with phototherapy. Frenectomy was performed due to tongue tie at 2 days old. She was discharged home on the third day of life.

In infancy she experienced frequent thrush and infections including viral gastroenteritis and bronchiolitis requiring multiple visits to the emergency department. She also has a history of failure to thrive in infancy in the setting of swallowing difficulty, frequent emesis and constipation. She required nasogastric tube feeds from 10 to 12 months old. Her emesis resolved by 2 years old. At 3 months old she was diagnosed with right occipital plagiocephaly in the setting of torticollis. She received physical therapy and wore a helmet between 7-12 months old.

At 11 months old, brain MRI and MRS showed mild nonspecific flattening of the optic discs bilaterally but was otherwise normal. She was seen by ophthalmology, and there were no concerns for papilledema, with normal examination. She has since been diagnosed with alternating exotropia and minimal refractive error, without concerns for amblyopia. Repeat brain MRI at 3 years old showed findings suggestive of Chiari 1 malformation. At 2 years old she was diagnosed with mild bilateral hip dysplasia and underwent bilateral Pemberton osteotomies at 5 years old. Echocardiogram, skeletal survey, abdominal ultrasound, and bone age studies have otherwise been normal.

At 2 years old she developed a facial rash for 9 months, which was treated with topical steroids. At 4 years old she was incidentally found to have Wolff-Parkinson-White syndrome and underwent cardiac ablation at 6 years old.

There were concerns for developmental delay from infancy. She rolled at 5 months old, sat at 9 months old, and crawled at 12 months old and walked unassisted at 20 months old. She spoke her first word at 2 years old. At 3 years old she was diagnosed with autism and ADHD. There have been multiple behavioral symptoms including aggressive behaviors, repetitive behaviors, and sensory sensitivities. She is treated with guanfacine 1 mg. She has been followed by neurology and is noted to have diffuse hypotonia on exam. At 8 years old an EEG was abnormal due to frequent occipital sharp waves over the right occipital region with rare bilateral synchronous occipital discharges. She has never had a seizure nor developmental regression.

She was initially evaluated by genetics at 9 months old. At that time, she was noted to have relative macrocephaly, frontal bossing, short and upturned nose, broad nasal bridge, epicanthus, low set left ear, high anterior hairline, and temporal narrowing. Clinical genetics evaluation has included fragile X testing, chromosomal microarray and trio exome and genome sequencing. Genome sequencing identified a maternally inherited heterozygous variant c.133G>A (p.Gly45Ser) in *HEPACAM* and a maternally inherited heterozygous variant c.4950A>C (p.Gln1650His) in *MED13L*, both of which were classified as uncertain significance. Biochemical testing has been uninformative including plasma amino acids, creatine kinase, urine organic acids, acylcarnitine profile, very long chain fatty acids, and total and free carnitine. Thyroid and growth hormone studies have been normal.

She is of North European and Ashkenazi Jewish ancestry. Consanguinity is denied. She has one older and one younger sister who are reportedly in good health. She has a maternal uncle with syndactyly, pre-axial polydactyly, and learning disabilities. Her maternal grandfather has dementia. Family history is otherwise negative for near relatives with developmental disorders.

She was evaluated through the Undiagnosed Diseases Network (UDN), at 6.5 years old. At that time, she was speaking in phrases and could count to ten. She was in a special education classroom and received adaptive physical education, occupational therapy, physical therapy, and speech therapy at her school. She also receives applied behavioral analysis. Her growth parameters at age 6.5 years old were OFC of 51.0 cm (-0.3 SD), weight of 15.0 kg (-2.5 SD), height of 102 cm (-3.0 SD). Trio genome sequencing was performed through the UDN sequencing core, Baylor Genetics, and was initially resulted negative. Analysis of the variant call sets at the UDN clinical site led to the identification of the apparently *de novo* heterozygous variant c.1772T>C (p.Met591Thr) in *KDM2A* in the proband. Sanger sequencing at Baylor Genetics was performed to confirm the *KDM2A* variant in the proband and segregation in the parents.

Individual 8, c.1796G>C, p.(Arg599Pro), *de novo*

This individual was referred to the genetic center at the age of 10 months old, because of a severe and syndromic IUGR. She is the first and only child of two unrelated and healthy parents. The parents' heights are 164 cm and 179 cm, respectively for the mother and the father. Pregnancy was spontaneous, with no reported teratogenic exposure. NIPT was performed because of an intermediate risk at the first trimester combined screening for trisomy 21. However, because of a family history of trisomy 21 in one of the mother's cousin, and because of a severe IUGR (below 1st centile) diagnosed at 24 weeks of amenorrhea, constitutional and molecular karyotypes were performed on fetal DNA extracted from amniotic fluid: both analyses were normal. Doppler-US were normal. CMV serology was negative in the mother blood. There was no anti-phospholipid syndrome, nor any other autoimmune disease. She was born prematurely by induced vaginal delivery at 36 weeks and 3 days because of the significant IUGR. Adaptation to extra-uterine life was excellent (Apgar 10/10/10/10). Birth weight was 1590 g (0.1st centile), birth length 41.5 cm (1st centile) and a birth OFC of 29.5 cm (2nd centile), confirming the harmonious IUGR. Placenta analyses showed a few calcifications, which may partly, but not entirely, explain the IUGR. CMV was controlled negative in a neonatal urine sample. Transfontanellar US-scan was normal, as well as neonatal hearing tests. However, the baby girl stayed 1 month in the neonatology unit because of this prematurity, and because of oral disorders initially linked to cow's milk proteins allergy complicated with rectorrhagia. Besides, she presented with a few morphological features, including a protruding metopic suture, up-slanted palpebral fissures, bilateral epicanthus, strabismus of the right eye, and a large mouth with a thin vermilion of the lower lip. Bilateral shoulders dimples were also noted. These features were not suggestive of a Silver-Russell syndrome. Moreover, she did not present with hypoglycemia. Temporary teeth eruption was delayed. We performed cardiac US-scan which was normal. Renal US-scan showed a small kidney stone.

Neuropsychological assessment was performed at 37 months of age, confirming a global development delay: developmental ages were estimated about 18 months old for fine motor skills, 24 months old for global motor skills, autonomy and socialization, and 30 months old for language. Neurological examination was normal as well as brain MRI which was performed at 2 years and 10 months old. She still has significant feeding problems. Weight grows around -3.5 SD, height grows around -2.5 DS and OFC grow around -2.5 DS. Growth hormone therapy has recently been introduced, at 4 years of age, even though GH deficiency has not been identified.

Individual 9, c.2327A>G, p.(Lys776Arg), *de novo*

The subject is a 13 year old male born from 29-year-old healthy father and 38-year-old healthy mother, who is high school educated. The parents are non-consanguineous. There is a family history of language delay. His two older brothers are not affected.

He was born at 32 weeks gestation via vaginal delivery after an uneventful pregnancy. There was no delivery or early neonatal complications. He sat and walked independently on time with no loss of acquired motor skills. His babbling was on time; however, he was delayed in speaking baba/mama, single words, or two-word sentence. He has learning disabilities but no intellectual disability, seizures, hyperactivity, inattention, or any behavioral problem. He was diagnosed to have autism at the age 3 years by ADI-R. Previously, he showed the loss of acquired social skills at 24 months but has shown considerable progress at the last assessment at age 13. He demonstrates average abilities in expressing and understanding other people's perspectives, and his communication skills are developing at a steady pace. He shares a good bond with his brothers, although he finds it challenging to connect with unfamiliar children. His ability to build and maintain relationships is also progressing at an average pace, and he shows a similar level of development in sharing interests. He attends a special school designed to cater to the needs of autistic children.

His orientation of time, place and person was normal, and recent memory was unaffected. He had no allergy, and his diet is normal. Special attention is being given to manage his weight. He is not taking any medication. His general appearance was normal as were his head and skin. No CT, brain MRI, or EEG was done.

At the age 3, the Fragile X molecular genetic testing showed one copy of chromosome X with 36 CGG repeats in the 5’-UTR in FMR-1 gene and it was interpreted as normal. Chromosome analysis, tuberous sclerosis, Rett syndrome, Angelman syndrome, Prader-Willi syndrome tests, or metabolic screening were not done.

Individual 10, c.2328G>T, p.(Lys776Asn), *de novo*

This individual is currently a 15-year-old male who was initially referred to Medical Genetics when he was 12-years-old due to a history of auditory processing disorder, motor delays, and epilepsy. His pregnancy was unremarkable. He was born at 34.5 weeks gestation and spent 12 days in the NICU due to prematurity. During his NICU stay, he required a feeding tube, however, he did not require one upon discharge. He did have delays early on, however, exact milestones are unknown. At 12-years-old, he was in a mixed special education and mainstream school program. He was in mainstream classes for history and science. He received speech and occupational therapy. In addition to Medical Genetics, he was followed by Neurology due to epilepsy, ADHD, and auditory processing disorder. He had a history of grand mal seizures for which a vEEG was done and revealed nocturnal episodes of eye opening 3-7x/night with vEEG correlate, suggesting possible nocturnal epilepsy. Brain MRI showed possible hippocampal asymmetry. His medical history is otherwise remarkable for an abnormal sleep schedule with excessive sleep, frequent naps, and snoring for which prior sleep study revealed borderline OSA. He was nondysmorphic. Exome sequencing was sent and revealed the following results:

- *KDM2A* c.2328G>T, p.Lys776Asn *de novo* heterozygous variant of uncertain significance
- *IRF2BP2* c.64C>A, p.Pro22Thr, *de novo* heterozygous variant of uncertain significance
- *SCN2A* c.5672 c.5672T>A, p.Val1891Asp paternally inherited variant of uncertain significance

More recently, he had a vEEG that captured two clinical seizures with right gaze deviation. He had generalized onset on EEG. There were frequent generalized epileptiform discharges.

Individual 11, c.2431C>A, p.(His811Asn), *de novo*

This boy presented with epileptic spasms at 5 months of age, unresponsive to multiple anti seizure medications (ASMs). Spasms came under control when pyridoxine was added one month later and all ASMs were discontinued by 1 year of age. Hypotonia was also noted, and he was slightly delayed in sitting and crawling until appropriate pyridoxine dose was reached. Language development was somewhat accelerated. He had physical therapy for about 2 years as a young child; walking was slightly delayed. He is described as uncoordinated and has difficulty participating in sports due to coordination issues and fatigability. He was eventually tapered off of pyridoxine when he was 6 years of age and remains seizure-free and off medications at age 14 years.

He participated in regular classes in school, though did have an education plan for social and sensory issues in elementary school. He was diagnosed for autism spectrum at 6 years old; he is quite social but has difficulty with social cues in many situations. In addition, he did perseverate until about 13 years. He had therapy for about 2 years as a young child for sensory integration and executive functioning. It is expected that he will be able to live independently as an adult.

Individual 12, c.58C>T, p.(Arg20*), *de novo*

Pregnancy was complicated by low fetal movements. This individual was born at 38 weeks 5 days gestation to a prima gravida mother. He weighed 2438 g with a length of 48 cm long at birth. He had hypoglycemia after birth, which resolved with formula. He always had height and weight in the lower centiles. His weight and height plateaued around 9-10 months of age. Bone age was delayed (bone age of 1 year with SD of 2.7 months with chronological age of 1 year 5 months). Thyroid function studies and IGF-1 and IGFBP-3 have been within normal limits. He was first noted to have hypotonia at 15 months. He had pes planus and increased flexibility of the ankles bilaterally. He had history of constipation, chronic cold, and frequent drooling. At 4 years old, he sustained a right non-displaced supracondylar fracture after a fall.

He was delayed with his developmental milestones. He walked independently around 18/19 months and first words came around 18 months. Speech has progressed well. He has been in special education classes and received developmental therapies. At 6 years old, he was having difficulties with writing and had fine motor delays. He always had behavioral issues and delayed processing speed.

The family history was significant for a younger brother with developmental delays, primarily in speech. Parents were non-consanguineous of Chinese and Dominican descent. Most recent clinical evaluation was at 5 years 11 months old and was significant for short stature, epicanthus, upslanted palpebral fissures, and protruding ears. Weight was 14.7 kg (-2.93 SD), height was 102.5 cm (-2.49 SD), and head circumference was 49.5 cm (-1.47 SD).

Duo exome testing identified the individual to be heterozygous for the variant c.58C>T, p.(Arg20*) in *KDM2A* that proved to of *de novo* origin after targeted testing of the other parent. Mt DNA testing identified the homoplasmic variant of uncertain significance m.7854T>C, p.Val90Ala in the *MT-CO2*. His mother also harbored the m.7854T>C variant in *MT-CO2* at an apparently homoplasmic level. He also had normal male karyotype, microarray, and methylation studies of chromosome 11 for Russell Silver syndrome. SMA copy number analysis showed 2 copies of SMN1.

Individual 13, c.579C>G, p.(Tyr193*), *de novo*

This individual is the product of conception of a cognitively normal non-consanguineous couple with unremarkable family history. The 36 years old father had inflammatory bowel disease and bipolar signs, the 39 years old mother was healthy. Paternal height was 185 cm, maternal height was 152 cm. The two older siblings were reportedly healthy with normal development. First trimester screening at 14 weeks of gestation (wog) was normal including an unremarkable nuchal translucency of 1.8 mm. Follow-up fetal ultrasound at 18 wog revealed intrauterine growth retardation (borderline low weight, abdominal circumference and head measurements just below the 5th centile) and prompted a search for TORCH infections and subsequent amniocentesis. Trisomy-Screening by quantitative-fluorescence-PCR, chromosomal microarray-analysis at a resolution of 100 kb using the Cytoscan HD array (Affymetrix®), methylation sensitive MLPA for the imprinted regions in 11p15 (Silver-Russell syndrome), 7p12.1, 7q32.2 and 14q32.2 revealed normal results for amniotic fluid DNA. Mutational screening by trio exome sequencing on DNA from native amniotic fluid using the Illumina® xGen® Exome Research Panel v2.0 (IDT) and the xGen Human mtDNA Research Panel v1.0 for target capturing and subsequent short-read paired-end sequencing on a NovaSeq 6000 device (Illumina®) revealed no obvious pathogenic variant in an established developmental disease gene. However, the *de novo* nonsense variant in *KDM2A* was considered a strong candidate variant given that the gene codes for a histone lysine demethylase and the known role of similar genes in developmental disorders. Sanger sequencing of DNA from the cultured material and both parents confirmed the *de novo* variant in the fetus. The gene was entered into the GeneMatcher database and due to the prompt and helpful feedback from colleagues who had observed other *de novo* truncating variants in individuals with disabling neurodevelopmental disease, the couple could be counselled accordingly. At 25+2 weeks of gestation, a fetal MRI of the head and whole body was performed, which confirmed microcephaly and did not reveal any other obvious anomaly. After careful consideration and interdisciplinary evaluation and counselling of the parents, the mother’s wish to terminate the pregnancy was carried out at 27 weeks of gestation.

Individual 14, c.1676dup, p.(Ile560Aspfs*71), *de novo*

This individual is a 13-year-old boy, first seen in a genetic consultation at the age of 4 years in the context of an acquisition delay. This is the second child of an unrelated, healthy couple. In the family history, there is a deafness in many people in the maternal family. The pregnancy was unremarkable with a vaginal delivery at 41 gestational weeks and 6 days with a good adaptation to extra uterine life. The birth measurements were within the norm. Regarding his development, he was sitting up at 9 months and walking at 18 months. He presented a delay in language, with the beginning of word association at 4 years. He also had difficulties with gross and fine motor skills. Re-educational assessments concluded that he had dyspraxia and ADHD with a cerebellar syndrome. He presented a delay in the acquisition of reading and writing. He benefits from a schooling in an adapted class and has reeducation by speech therapy, psychomotricity and occupational therapy. He has been treated with Methylphenidate for ADHD and Hydroxyzine for anxiety. Complementary ENT examinations were normal, brain MRI was normal and the ophthalmological examination showed mild hyperopia. On clinical examination, at the age of 13 years, he weighed 41.3 kg, was 160 cm tall, and had a head circumference of 53.8 cm (measurements within the norm for age). Cardiac and abdominal examinations were normal. On the skin, there were two café au lait spots on the supraorbital and abdominal areas. A left supernumerary nipple was noted. Neurologically, reflexes were present and sharp. A cutaneous-plantar reflex was in flexion. Walking was correct, as was tightrope walking. Orthopedically, there was an asymmetric pectus excavatum. Genetically, the search for fragile X syndrome was negative, FISH for 22q11 deletion syndrome was also negative and array CGH was normal. The trio exome performed revealed a *de novo* heterozygous frameshift variant in *KDM2A*.

Individual 15, c.1677delG, p.(Ile560Leufs*32), *de novo*

This individual is a 27-year male. He was born at the gestational age of 40 weeks and 1 day, with a low birthweight of 2510 g (2nd centile), length at birth 47 cm (<1st centile), OFC of 33 cm (3rd centile) after an uncomplicated pregnancy. He was operated on multiple cardiac anomalies: pulmonary valve stenosis, small VSD, persistent ductus arteriosus (PDA) and a secundum atrial septal defect, a 4-valved aortic valve with aortic insufficiency and double aortic arch. Moreover he had a duplicated left ureter, and reflux of the right kidney necessitating dilatation and bilateral ureteral reimplantation. Furthermore had an umbilical hernia and developed idiopathic chronic lymphedema of the lower abdomen and scrotal area. As a child he had recurrent otitis media.

His motor development was mildly delayed, with unassisted walking around the age of 2 years. His speech-development was delayed. First words at 18 months. He was in special education and at the ages of 6, 13 and 16 years a disharmonic profile was tested, with a verbal IQ of respectively 95, 80 and 72 and a performal IQ of 60, 61 and 56. A CT-scan of the brain at the age of 16 years was normal. At his current age, his verbal abilities are good and communication is normal.

He has mild dysmorphic features on physical examination, with a micrognathia, frontal upsweep of the hair and mild upslanted palpebral fissures.

His parents are non-consanguineous. He has a healthy older sister. His father has a spontaneously closed ventricle septal defect and premature atrial contractions. A paternal cousin anamnestically is on medication for a heart conduction disorder since the age of 18 years. Both a maternal uncle and maternal aunt have diabetes type 1, which were diagnosed in adulthood. A maternal cousin has diabetes type 2.

At the age of 19 years this individual developed diabetes type 1. Imaging showed a partial pancreatic agenesis and complete gallbladder agenesis. A mild, non-progressive pancytopenia was found repeatedly. Additional genetic testing with a gene-panel diabetes mellitus showed a heterozygous paternally inherited likely pathogenic nonsense variant in *GATA6* (NM_005257.4:c.779C>A, p.(Ser260*)). Although his father has no diabetes, the variant most likely explains, at least partially, this individual's partial pancreatic and complete gallbladder agenesis and hence the development of diabetes. It cannot be excluded that a maternally inherited, not identified, genetic factor is also present, considering the diabetes on the maternal side of the family. Cardiac anomalies can also be seen in individuals with *GATA6* pathogenic variants.

Individual 16, c.2404dup, p.(Thr802Asnfs*49), heterozygous

This male individual was result of a 41 week pregnancy without complications. The family history is unremarkable. Immediately before birth, the mother had a urinary tract and gastrointestinal infection. The birth weight was low (2nd centile) and later a reduced length growth was noted. Apart from this, perinatal and early infantile development were without abnormalities. He learned to sit without support at the age of 6 months, walking without support and other gross-motor skills were acquired age-appropriate as well. Since the age of 3 years, the boy is wearing glasses due to hyperopia and astigmatism. Hearing was good after tympanostomy tubes were inserted due to recurrent tympanic effusions.

Repeated endocrinological examinations due to the short stature revealed growth hormone levels in the lower normal and slightly reduced range, leading to somatotropin substitution since the age of 4 years. Initial language acquisition was reported to be normal. However, abnormalities in speech development emerged in kindergarten, alongside a delay in graphomotor skills was noted. Hence the boy receives occupational and speech therapy. A brain MRI at the age of 4 years revealed unspecific subcortical medullary lesions and a perivenular splenium-associated lesion on the right side. An EEG examination at the age of 5 years showed right frontal, temporo-occipital and left frontal prominent multifocal irregular sharp waves and spikes during sleep, without generalization. No clinical epileptic seizures have occurred to date.

Currently, he visits an integrative kindergarten. It is planned to send him to a regular school soon.

The radiological abnormalities, the speech development disorder and short stature prompted genetic testing with exome sequencing, which identified variants in *ABCD1* and *KDM2A* that were both determined to be of uncertain significance at the time of the initial analysis. The variant in *KDM2A* was later upgraded to likely pathogenic due to the fitting overlap to the rest of the cohort and the knowledge that truncating variants in *KDM2A* cause a neurodevelopmental disorder. Clinical, biochemically and brain MRI findings do not support the diagnosis of Adrenoleukodystrophy, typically associated with pathogenic *ABCD1* variants.

Individual 17, c.2667delC, p.(Asp889Glufs*47), *de novo*

This individual is a 14 year old male first seen due to seizures, intellectual impairments and behavioral concerns. He was born to a 22 year old G1P1 mother. His father was 26 at delivery. Consanguinity was denied. Prenatal exposures included maternal Ondansetron and Paracetamol use and exposure to cats. Pregnancy complications included maternal shingles. Prenatal testing (unclear of what type) showed an increased risk for Down syndrome, and bilateral club foot identified on prenatal ultrasound. He had casting and surgery until about age 4 years for his club feet. He was delivered at 39 weeks via spontaneous vaginal delivery. Birth parameters were length 45.7 cm (-3.0 SD) and weight 2608 g (-2.1 SD). His neonatal course was unremarkable.

His seizures began at the age of 4. This was also when his severe learning impairments were noted. He has been diagnosed with intellectual disability and is in a life skills classroom. He is reported to easily forget information, including spelling words and how to tie his shows. There is a history of regression in school (writing his numbers and letters backwards) and at home (he put on his clothes backwards, reverted back to playing with his little sister's dolls and can't tie his shoes.). He appears to have fairly normal social interactions with his peers and school and has friends. He also has short stature and has been following along the 2nd to 5th centile on the growth chart. However, his most recent height puts him in the 8th centile (152 cm). He wears glasses for myopia but does not wear them all the time. He also has been diagnosed with an astigmatism.

During the last physical exam inverted nipples, delayed coordination for age, instability on heel to toe walk, and mildly impaired balance were noted. His seizures are well controlled. His last seizure occurred at age 12.5 years. Overall his health has been good but his mother reports that he sleeps excessively and has been falling asleep in school. He sleeps through the night and does not snore or exhibit irregular breathing.

Individual 18, c.2809_2812dup, p.(Cys938*), *de novo*

The individual is an 11-year-old boy who was referred for the first time to a clinical geneticist at 16 months due to psychomotor delay and failure to thrive. He is the firstborn of an unrelated couple, conceived via *in vitr*o fertilization. During pregnancy, intrauterine growth retardation was noted around 33 weeks of gestation. Delivery took place at 35+3 weeks of gestation by vaginal delivery due to hypotrophy. He weighed 1.69 kg (-2.7 SD) with a length of 41 cm (-3.0 SD) and an OFC of 31.5 cm (-1.3 SD). He was hospitalized in neonatal care for 3 days due to neonatal jaundice.

Concerning developmental milestones, sitting up was acquired at 14 months age, he walked at 23 months and still only spoke a few words around 4 years. At 9 years he began to form complete sentences, with quite adequate comprehension skills. He demonstrated the ability to read and write simple sentences; however, he experiences difficulties concentrating during class and has approximately a two-year delay in his schooling. He requires a full-time school life assistant.

The physical examination revealed discrete facial characteristics, including low set ears, a thin upper vermilion of the lip with a long philtrum, retrognathia, and a long and narrow nose, along with strabismus. Examination of the extremities showed no remarkable findings. At 11 years old, his height is 139 cm (-0.5 SD), weight is 31.5 kg (-1.0 SD), and OFC is 55 cm (+1.26 SD). He has experienced growth retardation since birth, attributed to growth hormone (GH) deficiency diagnosed at 4 years old, for which he has been successfully supplemented. Premature puberty at 9 years old necessitated treatment with a gonadotropin-releasing hormone (GnRH) analogue to delay puberty.

Surgical correction was performed for cryptorchidism in 2020. In 2021, he required intensive care due to his second episode of pneumonia. Additionally, he experienced recurrent otitis, necessitating three trans-tympanic ventilation tube fittings. Subsequently, long-term antibiotic treatment (trimethoprim/sulfamethoxazole) was prescribed after evidence of CD4+ lymphocytopenia and low IgM levels were observed. Despite a well-conducted vaccination scheme, tetanus, pneumococcal, and diphtheria serology did not demonstrate protective levels.

The cardiac and abdominal ultrasound results were within normal limits; however, a left extra-sinus renal pelvis was detected. Subsequent brain MRI scans revealed normal findings, with the hypophysis noted to be small but within normal parameters. Additionally, bone age assessment showed normal results.

WISC test showed intelligence in the lower range of normal values, Vineland test showed results under first centile. Chromosome 7 methylation analysis ruled out Silver-Russel syndrome. CGH-Array was normal. Whole Exome Sequencing found a *POU1F1* VUS inherited from his mother, also found in his brother (with normal height at 6 years of age), this may have played a role in this GH deficiency.

Supplemental Note: Case report of an individual with a variant in *KDM2A* but insufficient evidence for causality

Individual S19, c.2323G>A, p.(Glu775Lys), heterozygous

This is a female currently 9 years-old with diagnoses of mild intellectual disability, autism spectrum disorder, ADHD, and history of global developmental delay (language, cognitive delays). She was adopted at age 2 months so limited information about her prenatal, birth, and family history is available. She was born at term following a pregnancy without known complications. She did well in the newborn period. She has history of delays in language and cognitive skills, now with diagnoses of mild ID (full scale IQ = 55) and autism spectrum disorder, as well as ADHD. Her growth parameters have been within the normal range, however her head circumference has always been on the larger side. No seizures, medical problems, or organ malformations and no dysmorphic features other than bilateral epicanthus, broad nasal bridge and broad nose were noted.

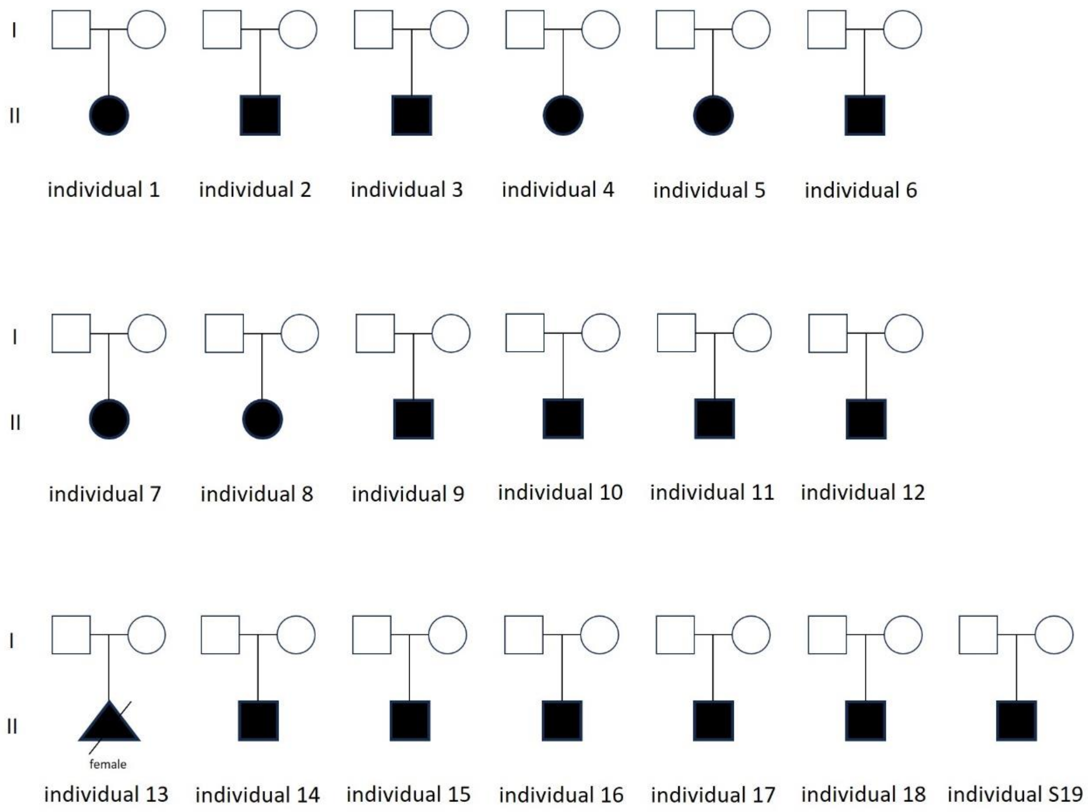

Figure S1. Pedigrees of all families

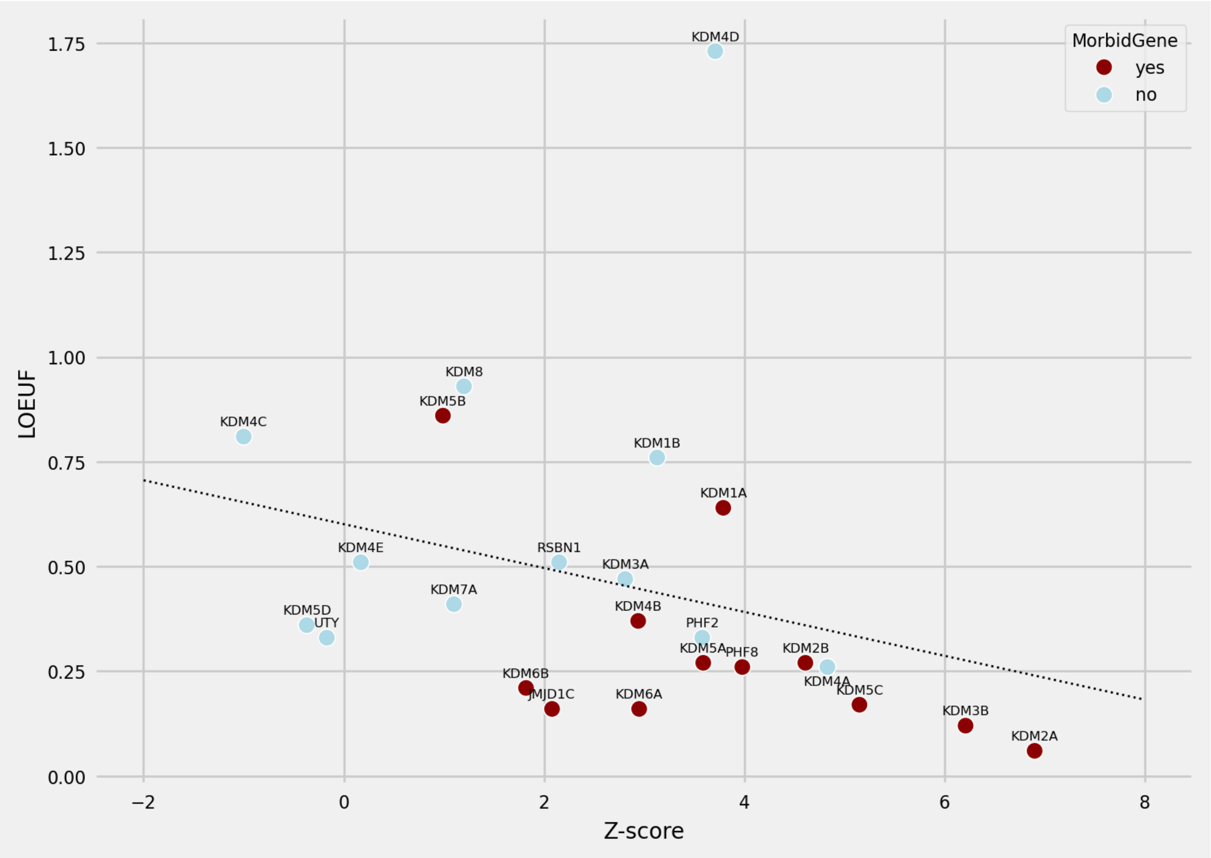

Figure S2. Constraint score landscape of KDM genes. Relationship between the LOEUF (predicted loss-of-function variants) and z-score (missense variants) among all KDM genes. *KDM2A* is the most constrained gene among this group with respect to both z-score and LOEUF, especially among MorbidGenes.[10] Constraint scores where preferably derived from gnomAD v4, but, if not available, also from gnomAD v2.1.1 (Table S6).[7] The dotted line indicates linear regression.

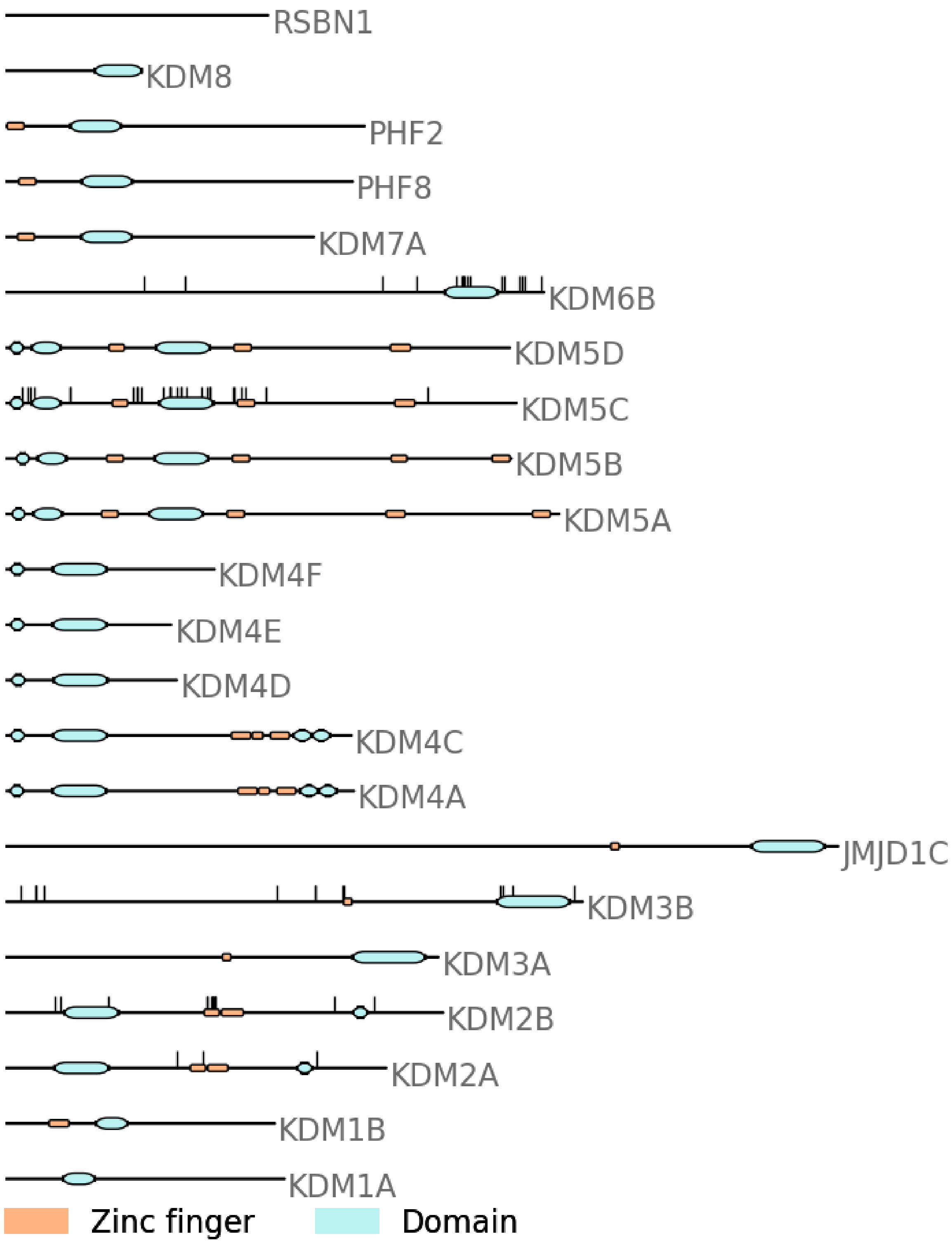

Figure S3. Overview of domain structure and reported variants in the literature in KDM genes. See Table S6 for a list of variants in KDM genes.

individual	Chr11: g.(hg38)	c.	p.	allelic state	origin	predicted effect	ACMG criteria[1]	classification
1	67207624	c.422A>G	p.(Tyr141Cys)	heterozygous	*de novo*	missense	PS2_MOD, PS3_MOD, PM2, PP2, PP3	likely pathogenic
2	67217747	c.704C>T	p.(Pro235Leu)	heterozygous	*de novo*	missense	PS2_MOD, PS3_MOD , PM2, PP2, PP3	likely pathogenic
3	67219296	c.850C>T	p.(His284Tyr)	heterozygous	*de novo*	missense	PS2_MOD, PM2, PP2, PP3	likely pathogenic
4	67219402	c.956G>A	p.(Arg319Gln)	heterozygous	*de novo*	missense	PS2_MOD, PM, PP2, PP3	likely pathogenic
5	67245196	c.1571T>G	p.(Phe524Cys)	heterozygous	*de novo*	missense	PS2_MOD, PM2, PP2	uncertain significance*
6	67245328	c.1703G>A	p.(Arg568Gln)	heterozygous	*de novo*	missense	PS2_MOD, PM2, PP2, PP3	likely pathogenic
7	67245397	c.1772T>C	p.(Met591Thr)	heterozygous	*de novo*	missense	PS2_MOD, PM2, PP2, PP3	likely pathogenic
8	67245421	c.1796G>C	p.(Arg599Pro)	heterozygous	*de novo*	missense	PS2_MOD, PM2, PP2, PP3	likely pathogenic
9	67250357	c.2327A>G	p.(Lys776Arg)	heterozygous	*de novo*	missense	PS2_MOD, PM2, PP2	uncertain significance*
10	67250358	c.2328G>T	p.(Lys776Asn)	heterozygous	*de novo*	missense	PS2_MOD, PM2, PP2	uncertain significance*
11	67250461	c.2431C>A	p.(His811Asn)	heterozygous	*de novo*	missense	PS2_MOD, PS3_MOD, PM2, PP2	likely pathogenic
12	67180094	c.58C>T	p.(Arg20*)	heterozygous	*de novo*	nonsense mediated mRNA decay	PVS1, PS2_MOD, PM2P	pathogenic
13	66982903	c.579C>G	p.(Tyr193*)	heterozygous	*de novo*	nonsense mediated mRNA decay	PVS1, PS2_MOD, PM2	pathogenic
14	67245301dup	c.1676dup	p.(Ile560Aspfs*71)	heterozygous	*de novo*	nonsense mediated mRNA decay	PVS1, PS2_MOD, PM2	pathogenic
15	67245302del	c.1677del	p.(Ile560Leufs*32)	heterozygous	*de novo*	nonsense mediated mRNA decay	PVS1, PS2_MOD, PM2	pathogenic
16	67250434dup	c.2404dup	p.(Thr802Asnfs*49)	heterozygous	unknown	nonsense mediated mRNA decay	PVS1, PM2	likely pathogenic
17	67250697del	c.2667del	p.(Asp889Glufs*47)	heterozygous	*de novo*	nonsense mediated mRNA decay	PVS1, PS2_MOD, PM2	pathogenic
18	67252734_ 67252737dup	c.2809_ 2812dup	p.(Cys938*)	heterozygous	*de novo*	nonsense mediated mRNA decay	PVS1, PS2_MOD, PM2	pathogenic

Table S1. Variant information and classification according to ACMG criteria.[1] MANE Select transcript NM_012308.3 was used. *Due to the *de novo* status of the variant in addition to the fitting clinical overlap to the rest of the cohort, this variant is deemed causative despite being classified as uncertain.

Indi-visual	Chr11: g.(hg38)	c.	p.	CADD-v1.6[2]	REVEL[3]	MutPred2[4]	VEST4[5]	BayesDel[6]	AA conservation (conserved up to)	gnomAD v4[7]
1	67207624	c.422A>G	p.(Tyr141Cys)	32.0	0.836	0.522	0.858	0.389	high (c. elegans)	0
2	67217747	c.704C>T	p.(Pro235Leu)	34.0	0.880	0.594	0.821	0.426	high (zebrafish)	0
3	67219296	c.850C>T	p.(His284Tyr)	27.6	0.954	0.860	0.894	0.567	high (c. elegans)	0
4	67219402	c.956G>A	p.(Arg319Gln)	34.0	0.465	0.426	0.518	0.195	high (c. elegans)	0
5	67245196	c.1571T>G	p.(Phe524Cys)	21.0	0.040	0.334	0.286	-0.099	moderate (platypus)	0
6	67245328	c.1703G>A	p.(Arg568Gln)	32.0	0.473	0.655	0.660	0.271	high (c. elegans)	0
7	67245397	c.1772T>C	p.(Met591Thr)	27.3	0.653	0.615	0.820	0.347	high (zebrafish)	0
8	67245421	c.1796G>C	p.(Arg599Pro)	33.0	0.531	0.611	0.800	0.314	high (frog)	0
9	67250357	c.2327A>G	p.(Lys776Arg)	23.8	0.096	0.298	0.272	-0.081	high (frog)	0
10	67250358	c.2328G>T	p.(Lys776Asn)	23.5	0.109	0.313	0.381	-0.164	high (frog)	0
11	67250461	c.2431C>A	p.(His811Asn)	22.1	0.104	0.479	0.298	-0.234	moderate (chicken)	0

Table S2. *In silico* prediction of missense variants in *KDM2A*.

MANE Select transcript NM_012308.3 was used. *In silico* scores were retrieved using dbNSFP[8]. Cutoffs for *in silico* scores were derived from Pejaver *et al.*[9] Red color signifies damaging prediction (at least PP3_SUP score in Pejaver *et al.* was reached); yellow color signifies neither PP3_SUP nor BP4_SUP score was reached; green color signifies benign prediction (at least BP4_SUP score in Pejaver *et al.* was reached). For further annotations on the missense variants, see Table S5 in the separate excel file.

Table S3. Detailed clinical data of individuals with causative variants in *KDM2A*

See separate Excel file.

Table S4. Detailed clinical data of an individual with variant in *KDM2A* but insufficient evidence for causality

See separate Excel file.

Table S5. Annotation and *in silico* scores of all missense variants in *KDM2A*

See separate excel file. All annotations were retrieved using dbNSFP v4.5.[8]

Table S6. List of KDM genes

See separate Excel file.

Table S7. Reported variants in the literature in KDM genes

See separate Excel file.

Table S8. Methylation data of the *KDM2A*-related Episignature

See separate Excel file. Differential methylated regions between cases and control group. DMRs were filtered for an adjusted p-value of <0.01 and minimal methylation difference of >10%.

References

1. Richards, S., Aziz, N., Bale, S., Bick, D., Das, S., Gastier-Foster, J., Grody, W.W., Hegde, M., Lyon, E., Spector, E., et al. (2015). Standards and guidelines for the interpretation of sequence variants: a joint consensus recommendation of the American College of Medical Genetics and Genomics and the Association for Molecular Pathology. Genetics in Medicine *17*, 405–424. https://doi.org/10.1038/gim.2015.30.

2. Rentzsch, P., Schubach, M., Shendure, J., and Kircher, M. (2021). CADD-Splice—improving genome-wide variant effect prediction using deep learning-derived splice scores. Genome Med *13*, 31. https://doi.org/10.1186/s13073-021-00835-9.

3. Ioannidis, N.M., Rothstein, J.H., Pejaver, V., Middha, S., McDonnell, S.K., Baheti, S., Musolf, A., Li, Q., Holzinger, E., Karyadi, D., et al. (2016). REVEL: An Ensemble Method for Predicting the Pathogenicity of Rare Missense Variants. The American Journal of Human Genetics *99*, 877–885. https://doi.org/10.1016/j.ajhg.2016.08.016.

4. Pejaver, V., Urresti, J., Lugo-Martinez, J., Pagel, K.A., Lin, G.N., Nam, H.-J., Mort, M., Cooper, D.N., Sebat, J., Iakoucheva, L.M., et al. (2020). Inferring the molecular and phenotypic impact of amino acid variants with MutPred2. Nat Commun *11*, 5918. https://doi.org/10.1038/s41467-020-19669-x.

5. Carter, H., Douville, C., Stenson, P.D., Cooper, D.N., and Karchin, R. (2013). Identifying Mendelian disease genes with the Variant Effect Scoring Tool. BMC Genomics *14*, S3. https://doi.org/10.1186/1471-2164-14-S3-S3.

6. Feng, B.-J. (2017). PERCH: A Unified Framework for Disease Gene Prioritization. Human Mutation *38*, 243–251. https://doi.org/10.1002/humu.23158.

7. Chen, S., Francioli, L.C., Goodrich, J.K., Collins, R.L., Kanai, M., Wang, Q., Alföldi, J., Watts, N.A., Vittal, C., Gauthier, L.D., et al. (2024). A genomic mutational constraint map using variation in 76,156 human genomes. Nature *625*, 92–100. https://doi.org/10.1038/s41586-023-06045-0.

8. Liu, X., Li, C., Mou, C., Dong, Y., and Tu, Y. (2020). dbNSFP v4: a comprehensive database of transcript-specific functional predictions and annotations for human nonsynonymous and splice-site SNVs. Genome Medicine *12*, 103. https://doi.org/10.1186/s13073-020-00803-9.

9. Pejaver, V., Byrne, A.B., Feng, B.-J., Pagel, K.A., Mooney, S.D., Karchin, R., O'Donnell-Luria, A., Harrison, S.M., Tavtigian, S.V., Greenblatt, M.S., et al. (2022). Calibration of computational tools for missense variant pathogenicity classification and ClinGen recommendations for PP3/BP4 criteria. The American Journal of Human Genetics *109*, 2163–2177. https://doi.org/10.1016/j.ajhg.2022.10.013.

10. Jauss, R.-T., Popp, B., Bachmann, J., Abou Jamra, R., and Platzer, K. (2024). The MorbidGenes panel: a monthly updated list of diagnostically relevant rare disease genes derived from diverse sources. Hum. Genet. https://doi.org/10.1007/s00439-024-02711-z.
