# Supplementary material for: De novo variants in KDM2A cause a syndromic neurodevelopmental disorder
Source: Am J Hum Genet. 2025 Dec 29;113(1):100–16. doi: 10.1016/j.ajhg.2025.12.004 (PMC12824617; doi:10.1016/j.ajhg.2025.12.004)
Supplement: Document S2. Article plus supplemental information [file mmc3.pdf]

De novo variants in *KDM2A* cause a syndromic neurodevelopmental disorder

Authors

Eric N. Anderson, Stephan Drukewitz, Sukhleen Kour, ..., Amit Singh, Udai Bhan Pandey, Konrad Platzer

Correspondence

udai@pitt.edu (U.B.P.), konrad.platzer@medizin.uni-leipzig.de (K.P.)

***De novo* variants in *KDM2A* cause a syndromic neurodevelopmental disorder with a phenotypic spectrum of mild to severe developmental delay, feeding difficulties, short stature, microcephaly, and recurrent facial features. Functional studies in human cells and *Drosophila*, as well as aberrant methylation profiles, support the gene-disease association.**

Anderson et al., 2026, The American Journal of Human Genetics *113*, 100–116
January 8, 2026 © 2025 The Authors. Published by Elsevier Inc. on behalf of American Society of Human Genetics.
https://doi.org/10.1016/j.ajhg.2025.12.004

CellPress

ARTICLE

De novo variants in *KDM2A* cause a syndromic neurodevelopmental disorder

Eric N. Anderson,[1] Stephan Drukewitz,[2] Sukhleen Kour,[1] Anuradha V. Chimata,[3] Deepa S. Rajan,[1] Senta Schönnagel,[2] Karen L. Stals,[4] Deirdre Donnelly,[5] Siobhan O'Sullivan,[5] John F. Mantovani,[6] Tiong Y. Tan,[7,8] Zornitza Stark,[7,8] Pia Zacher,[9] Nicolas Chatron,[10] Pauline Monin,[10] Severine Drunat,[11] Yoann Vial,[11] Xenia Latypova,[11] Jonathan Levy,[11] Alain Verloes,[11] Jennefer N. Carter,[12,13] Devon E. Bonner,[12,13] Suma P. Shankar,[14] Jonathan A. Bernstein,[12,13] Julie S. Cohen,[15,16] Anne Comi,[15,16] Deanna Alexis Carere,[17] Lisa M. Dyer,[17] Sureni V. Mullegama,[17] Pedro A. Sanchez-Lara,[18] Katheryn Grand,[18] Hyung-Goo Kim,[19] Afif Ben-Mahmoud,[20] Sidney M. Gospe, Jr.,[21,22] Rebecca S. Belles,[23] Gary Bellus,[23] Klaske D. Lichtenbelt,[24] Renske Oegema,[24] Anita Rauch,[25] Ivan Ivanovski,[25] Frederic Tran Mau-Them,[26,27] Aurore Garde,[26] Rachel Rabin,[28] John Pappas,[28] Annette E. Bley,[29] Janna Bredow,[29] Timo Wagner,[30] Eva Decker,[30] Carsten Bergmann,[30] Louis Domenach,[31] Henri Margot,[31] Undiagnosed Diseases Network, Johannes R. Lemke,[2,32] Rami Abou Jamra,[2] Julia Hentschel,[2] Heather Mefford,[33] Amit Singh,[3] Udai Bhan Pandey,[1,34,35,*] and Konrad Platzer[2,35,*]

Summary

Germline variants that disrupt components of the epigenetic machinery cause syndromic neurodevelopmental disorders. Using exome and genome sequencing, we identified *de novo* variants in *KDM2A*, a lysine demethylase crucial for embryonic development, in 18 individuals with developmental delays and/or intellectual disabilities. The severity ranged from learning disabilities to severe intellectual disability. Other core symptoms included feeding difficulties; growth issues, such as intrauterine growth restriction, short stature, and microcephaly; and recurrent facial features, such as epicanthic folds, upslanted palpebral fissures, thin vermillion of the lips, and low-set ears. Expression of human disease-causing *KDM2A* variants in a *Drosophila melanogaster* model led to neural degeneration, motor defects, and reduced lifespan. Interestingly, pathogenic variants in *KDM2A* affected physiological attributes, including subcellular distribution, expression, and stability in human cells. Genetic epistasis experiments indicated that *KDM2A* variants act via a dual mechanism—loss of nuclear function for some variants tested and additional cytoplasmic gain-of-function toxicity for c.704C>T (p.Pro235Leu), as eliminating endogenous *Drosophila Kdm2* did not produce noticeable neurodevelopmental phenotypes. Data from enzymatic-methylation sequencing support the suggested gene-disease association by showing aberrant methylome profiles in affected individuals' peripheral blood. Combining our genetic, phenotypic, and functional findings, we establish *de novo* variants in *KDM2A* as causative for a syndromic neurodevelopmental disorder.

[1]Department of Pediatrics, Children's Hospital of Pittsburgh, University of Pittsburgh Medical Center, Pittsburgh, PA 15224, USA; [2]Institute of Human Genetics, University of Leipzig Medical Center, 04103 Leipzig, Germany; [3]Department of Biology, University of Dayton, Dayton, OH, USA; [4]Royal Devon & Exeter NHS Foundation Trust, Exeter Genomics Laboratory, Exeter EX2 5DW, UK; [5]Northern Ireland Regional Genetics Centre, Belfast Health and Social Care Trust/City Hospital, Belfast, Northern Ireland BT9 7AB, UK; [6]Division of Child Neurology, Washington University School of Medicine, Mercy Kids Center for Neurodevelopment & Autism, St. Louis, MO 63110, USA; [7]Victorian Clinical Genetics Services, Murdoch Children's Research Institute, Melbourne, VIC, Australia; [8]Department of Paediatrics, University of Melbourne, Melbourne, VIC, Australia; [9]Epilepsy Center Kleinwachau, 01454 Radeberg, Germany; [10]Department of Medical Genetics, University Hospital of Lyon, 69007 Lyon, France; [11]Department of Genetics, APHP-Robert DEBRE University Hospital, Sorbonne Paris-Cité University, and INSERM UMR, 1141 Paris, France; [12]Stanford Center for Undiagnosed Diseases, Stanford University, Stanford, CA 94305, USA; [13]Department of Pediatrics, Division of Medical Genetics, Stanford University School of Medicine, Stanford, CA 94305, USA; [14]Departments of Pediatrics & Ophthalmology, Genomic Medicine, University of California, Davis Health, Sacramento, CA 95817, USA; [15]Department of Neurology and Developmental Medicine, Kennedy Krieger Institute, Baltimore, MD 21205, USA; [16]Department of Neurology, Johns Hopkins University School of Medicine, Baltimore, MD 21287, USA; [17]GeneDx, LLC, Gaithersburg, MD 20877, USA; [18]Department of Pediatrics, Cedars-Sinai Medical Center, Los Angeles, CA 90048, USA; [19]Department of Neurosurgery, Robert Wood Johnson Medical School, Rutgers University, Piscataway, NJ 08854, USA; [20]Neurological Disorder Research Center, Qatar Biomedical Research Institute, Qatar Foundation, Hamad Bin Khalifa University, Doha, Qatar; [21]Departments of Neurology and Pediatrics, University of Washington School of Medicine, Seattle, WA, USA; [22]Department of Pediatrics, Duke University, Durham, NC, USA; [23]Geisinger Health System, Danville, PA 17821, USA; [24]Department of Genetics, Utrecht University Medical Center, 3584 EA Utrecht, the Netherlands; [25]Institute of Medical Genetics, University of Zurich, 8952 Schlieren, Zurich, Switzerland; [26]Laboratoire de Génomique Médicale – Centre NEOMICS, CHU Dijon Bourgogne, 21000 Dijon, France; [27]INSERM – Université de Bourgogne - UMR1231 GAD, 21000 Dijon, France; [28]Clinical Genetic Services, Department of Pediatrics, NYU School of Medicine, New York, NY 10016, USA; [29]Leukodystrophy Clinic, University Children's Hospital, University Medical Center, 20246 Hamburg, Germany; [30]Medizinische Genetik Mainz, Limbach Genetics GmbH, Mainz, Germany; [31]Department of Medical Genetics, MRGM INSERM U1211, Bordeaux University Hospital, University of Bordeaux, Bordeaux, France; [32]Center for Rare Diseases, University of Leipzig Medical Center, 04103 Leipzig, Germany; [33]Center for Pediatric Neurological Disease Research, St. Jude Children's Research Hospital, Memphis, TN 38105, USA; [34]Children's Neuroscience Institute, Children's Hospital of Pittsburgh, Pittsburgh, PA 15224, USA
[35]These authors contributed equally
*Correspondence: udai@pitt.edu (U.B.P.), konrad.platzer@medizin.uni-leipzig.de (K.P.)
https://doi.org/10.1016/j.ajhg.2025.12.004.
© 2025 The Authors. Published by Elsevier Inc. on behalf of American Society of Human Genetics.
This is an open access article under the CC BY license (http://creativecommons.org/licenses/by/4.0/).

Introduction

The epigenetic machinery encompasses proteins that function as writers, erasers, readers, and remodelers of epigenetic marks on DNA and histones. The eraser KDM2A removes mono- and di-methylation from histone 3 at lysine 36 (H3K36). The post-translational demethylation of lysine residues on histone tails mediated by histone lysine demethylases (KDMs), and specifically by KDM2A, is an important player in gene regulation and has shown to be crucial for embryonic development and processes such as proliferation, apoptosis, and differentiation.[1]

Pathogenic germline variants in genes of the epigenetic machinery cause a now-established group of rare Mendelian disorders.[2,3] Within this broad group, variants disrupting KDMs are a frequent cause of neurodevelopmental disorders (NDDs),[4–6] which encompass a heterogeneous group of conditions characterized by aberrant brain development and function, leading to cognitive, motor, and behavioral impairments. Previously, two *de novo* missense and one *de novo* frameshift variant in *KDM2A* (MIM: 605657) were described in three individuals with autism and an NDD but only as members of much broader cohorts looking into the genetic architecture of these phenotypes.[7–9]

Here, we describe the overlapping phenotype of 18 individuals with *de novo* variants in *KDM2A*. We combined the power of genetically modified *Drosophila melanogaster*, which our lab has successfully used to in the delineation of other novel NDDs[10–12] with human cell culture, as well as methylome data based on blood-derived DNA from affected individuals, to establish the gene-disease association of *de novo* variants in *KDM2A* and a syndromic NDD.

Subjects and methods

Recruitment of affected individuals and consent

This study was approved by the ethics committee of the University of Leipzig (402/16-ek). Written informed consent for molecular genetic testing and data publication was obtained from all individuals and/or their legal representatives by the referring physicians according to the guidelines of the ethics committees and institutional review boards of the respective institutes. After the initial identification of individual 4 with a *de novo* missense variant in *KDM2A*, the compilation of the cohort with *de novo* variants in *KDM2A* and overlapping phenotypes was supported by international collaboration, personal communication, and online matchmaking via GeneMatcher.[13] Phenotypic and genotypic information was obtained from the referring collaborators using a standardized questionnaire.

Variant identification

Trio exome or genome sequencing was performed for all affected individuals and their parents except for individuals 12 (duo exome with targeted variant testing of the other parent) and 16 (singleton exome) using standard methodologies and commercial sequencing kits for short-read massively parallel sequencing. Analysis of the sequencing data was performed according to the local diagnostic protocols of the respective centers, primarily focusing on genes where variants are known to cause a rare disease. Since no causative variants were identified in this gene set, research evaluation of the sequencing data was done afterward to potentially identify causative variants in candidate genes not previously associated with a rare disease, such as *KDM2A*. After identification of a rare variant in *KDM2A* in one of the contributing centers, the matchmaking process followed the steps described above to compare genotype and phenotype and potentially contribute to the project. Overall, we were able to recruit 18 individuals with an overlapping neurodevelopmental phenotype, and the identified rare variants were determined to fit the project: proven *de novo* origin for the variant absent in gnomAD v.4 and/or aberrant methylation data supporting a diagnosis of a *KDM2A*-related condition. There were no significant findings, apart from the described variants in *KDM2A*, which likely explain the neurodevelopmental phenotypes of the respective individuals. The gnomAD v.4 dataset served as the control population to determine allele frequency.[14] All variants described were aligned to hg38, mapped to the *KDM2A* MANE Select transcript GenBank: NM_012308.3, and classified according to ACMG criteria retrospectively in light of the knowledge of the entire cohort (Table S1).[15,16] One additional individual is described in the supplemental information and not included in the main cohort (individual S19; for a detailed phenotypic description, see the supplemental notes and Table S4), as the origin of the identified variant could not be tested in the parents, and the analysis of the methylation data did not support a diagnosis of a *KDM2A*-related disorder.

In silico prediction

Missense variants were assessed using CADD-v.1.6,[17] REVEL,[18] MutPred2,[19] VEST4,[20] and BayesDel[21] using deleterious prediction cutoffs defined by Pejaver et al.[22] (Table S2).

Drosophila stock

To model the human variants c.704C>T (GenBank: NM_012308.3) (p.Pro235Leu), c.422A>G (GenBank: NM_012308.3) (p.Tyr141Cys), and c.2431C>A (GenBank: NM_012308.3) (p.His811Asn), the wild-type *KDM2A* (*KDM2A*-WT), *KDM2A*-Pro235Leu, *KDM2A*-Tyr141Cys, and *KDM2A*-His811Asn lines were generated by site-specific insertion of the transgene at BestGene (Chino Hills, CA, USA) using the attP2 insertion vector as previously done.[23] All *Drosophila* stocks were maintained on standard cornmeal medium at 29°C in light/dark-controlled incubators. ELAV-gal4 (#8760), glass multiple reporter (GMR)-gal4 (#1104), *Kdm2*^{TG4} (#94591), *Kdm2* RNAi (#31360), and luciferase (#35788) were obtained from the Bloomington Drosophila Stock Center (Bloomington, IN, USA).

Climbing assay

The rapid iterative negative geotaxis (RING) assay was done as previously described.[11,24,25] Briefly, *Drosophila* expressing *KDM2A* variants or luciferase pan-neuronally were aged for 20 days before being transferred to fresh vials. Flies were knocked three times on the base of a bench, and a video camera was used to record the flies climbing up the wall of the vials. The velocity (cm/s) was calculated and analyzed from three independent experiments using GraphPad Prism 6 (Boston, MA, USA).

Lifespan assay

The lifespan assay was performed as previously described.[11,24] Briefly, *Drosophila* expressing *KDM2A* variants or luciferase

pan-neuronally were raised on standard cornmeal food. 1- to 3-day-old adult progeny flies were transferred to fresh food twice a week, the number of dead flies was counted every day, and survival functions were calculated and plotted as Kaplan-Meier survival curves. Log rank with Grehan-Breslow-Wilcoxon tests were performed to determine the significance of differences in survival data between the groups using GraphPad Prism 6 software.

Eye severity experiments in *Drosophila*

A GMR promoter element (GMR-gal4) was used to cross *KDM2A* or luciferase in the eyes. Images of the right eyes from F1-generation adult female *Drosophila* were taken at day 1 using a Leica M205C (Deer Park, IL, USA) dissection microscope equipped with a Leica DFC450 camera. External eye severity was quantified using a previously published scoring system.[26] Statistical analyses were performed using GraphPad Prism 6, with group comparisons performed using one-way ANOVA.

Drosophila Kdm2 knockdown and knockout experiments

To knock down *Kdm2* in the eye, we used the GAL4/UAS system. *Drosophila* carrying the eye-specific driver GMR-Gal4 were combined with UAS-*Kdm2*-RNAi and crossed together with one of the following UAS transgenes in the same background: human *KDM2A*-WT, *KDM2A*-Pro235Leu, *KDM2A*-Tyr141Cys, *KDM2A*-His811Asn, or UAS-luciferase (control). Progeny were reared at 25°C, and day 1 adult eyes were imaged as described above.

Kdm2 was genetically inactivated by φC31-mediated recombination-mediated cassette exchange (RMCE) of MiMIC insertion lines as previously described.[27] Briefly, *Drosophila* bearing a MiMIC element within the *Kdm2* locus were crossed to UAS-2×EGFP; hs-Cre; vas-φC31 integrase; Trojan T2A-GAL4 flies following the scheme of Diao et al.[28] Resulting progeny underwent heat shock to induce Cre-mediated excision and φC31-driven RMCE, replacing the MiMIC cassette with the Trojan T2A-GAL4 reporter to generate *Kdm2*-TG4 alleles. *Kdm2*-TG4 *Drosophila* were then crossed to the same panel of UAS-human *KDM2A* or UAS-luciferase lines. Negative geotaxis (climbing) assays and lifespan analyses were carried out on adult progeny as described above.

Western blotting

Drosophila

On day 1, heads from the adult female F1 generation were collected from each cross and snap frozen on dry ice. Five heads were used per lane of the western blots (WBs). Heads were crushed on dry ice and incubated in RIPA buffer containing 150 mM NaCl, 1% NP40, 0.1% SDS, 1% sodium deoxycholate, 50 mM NaF, 2 mM EDTA, 1 mM DTT, 0.2 mM sodium orthovanadate, and 1× protease inhibitor cocktail (Roche, San Francisco, CA, USA; 11836170001). Lysates were sonicated and centrifuged to remove exoskeletal debris. Supernatants were boiled in Laemmli buffer (Boston Bioproducts, Milford, MA, USA; BP-111R) for 5 min, and proteins were separated using 3%–8%, NuPAGE tris-acetate gels (Thermo Fisher Scientific, Waltham, MA, USA: EA03785BOX). Proteins were transferred onto nitrocellulose membranes (iBlot 2 transfer stacks; Invitrogen, Carlsbad, CA, USA; IB23001) using the iBlot2 system (Life Technologies, Waltham, MA, USA; 13120134). Membranes were blocked in milk (BLOT-QuickBlocker reagent; EMD Millipore, Burlington, MA, USA; WB57-175GM) and incubated overnight in primary antibody (rabbit anti-KDM2A, Abcam; ab191387; 1:1000; Waltham, MA, USA). Blots were washed and incubated in secondary antibody for 1 h (anti-rabbit, DYLight 800, Pierce, 1:10,000; Waltham, MA, USA). Imaging was performed using the Odyssey CLx (LI-COR Biosciences, Lincoln, NE, USA). Protein levels were quantified using Image Studio (LI-COR Biosciences), and statistical analyses were performed with GraphPad Prism 6. All WBs were performed in triplicate using biological replicates.

Mammalian cells

Human embryonic kidney 293T (HEK293T) cells from ATCC were cultured in advanced Dulbecco's modified Eagle's medium (DMEM) (Gibco, Waltham, MA, USA; 12491023) containing 10% FBS (Biowest, Riverside, MO, USA; S01520) and 1× GlutaMAX (Gibco; 35050079). Cells were lysed by boiling for 5 min in 1× LDS sample buffer (Invitrogen; NP-0007) and RIPA buffer. All NuPAGE and western blotting steps were performed as described above. Experiments were performed in triplicate using three independent lysate preparations from cultured cells.

Plasmids

KDM2A-WT-HA (VB: 220629-1403rdb), *KDM2A*-Pro235Leu-HA (VB220629-1413ecm), *KDM2A*-Tyr141Cys-HA (VB: 220629-1409 axj), and *KDM2A*-811N-HA (VB: 220629-1405kmm) were constructed by VectorBuilder (Chicago, IL, USA).

Immunofluorescence

HEK293T cells transfected with the *KDM2A* plasmid grown on coverslips were rinsed in PBS (Lonza 17-512F) and fixed in 4% paraformaldehyde (Millipore Sigma P6148, Burlington, MA, USA) for 20 min at room temperature. Following fixation, the samples were washed four times (×10 min) in PBS and blocked with blocking buffer: 5% normal goat serum (NGS; Abcam AB7681) in PBS with 0.1% Triton X-100 (PBST). The samples were incubated overnight at 4°C with primary antibody rabbit anti-*KDM2A* (1:1,000) and mouse anti-HA (H3663; 1:1,000; Millipore Sigma), washed four times (×10 min) with 0.1% PBST, and incubated with secondary antibody (goat anti-mouse Alexa Fluor 568, A22287: 1:500, and goat anti-rabbit Alexa Fluor 488, A-11008: 1:500, Invitrogen) for 2 h at room temperature, followed by 0.1% PBST washes. Samples were mounted onto slides using Fluoroshield (Sigma-Aldrich F6057, St. Louis, MO, USA).

Nuclear-cytoplasmic fractionation

HEK293T cells transfected with *KDM2A*-WT or *KDM2A* variants (p.Pro235Leu, p.Tyr141Cys, and p.His811Asn) were harvested, and nuclear-cytoplasmic fractionation was done using the NE-PER nuclear-cytoplasmic extraction kit per the manufacturer's protocol (Thermo Fisher Scientific).

Cycloheximide chase assay

To evaluate the stability of the *KDM2A*-WT and the p.Pro235Leu variant, HEK293T cells were transfected with the *KDM2A* plasmids and incubated for 24 h. Protein synthesis was then inhibited by the addition of 0.5 mg/mL cycloheximide (CHX). At 5 different time points (0, 6, 12, 24, and 48 h after CHX addition), the cells were harvested, and the samples' supernatants were subjected to immunoblot analysis with HA antibody to recognize KDM2A and loading control Tubulin.

Methylome analysis and episignature

DNA was extracted from peripheral blood using standard protocols at the respective centers. DNA quantity was measured using Qubit 4 and the Qubit double-stranded DNA (dsDNA) BR Assay-Kit (Thermo Fisher Scientific, Darmstadt, Germany). A total of 200 ng of DNA was used for ultrasonic fragmentation of the DNA with a focused ultrasonicator (ME220, Covaris, MA, USA).

Library preparation was done using the NEB Enzymatic Methyl-seq Library Preparation Kit. A 187.5 ng library was used for capture with the Twist Human Methylome Panel according to the manufacturer's protocol (TWIST Bioscience, South San Francisco, CA, USA). This panel targets 5.549 million CpG sites (in comparison: on the EPIC v.2.0 array, 0.930 million CpG sites are covered; 0.163 million CpGs are unique in the array, while 4.782 million are unique in the methylome panel). 2×150 bp sequencing was done on one lane of an S4 cartridge (s4 reagent kit [300 cycles], run on a NovaSeq6000 [Illumina, San Diego, CA, USA]). The average coverage of target regions was 120×. As a control group, DNA from the peripheral blood of healthy age- and sex-matched individuals was selected. Raw reads were quality checked using FastQC and trimmed using cutadapt.[29] Trimmed data were aligned to GRCh38 using BWA-meth, and duplicates were marked using Picard – MarkDuplicates. Methylation calls were extracted from the deduplicated alignment files using MethylDackel. Bedgraphs were filtered for regions covered >30×. Calling of differentially methylated regions (DMRs) between case and control groups was done using metilene with the following settings: a minimum (min.) length of CpG of ≥10, a min. length in nt of >0, a min. absolute methylation difference of ≥0.1, and an adjusted *p* [*p*-adj] < 0.05.[30] To generate an episignature of the *KDM2A* cases, called DMRs, were filtered using an *p*-adj < 0.01 and a minimal absolute methylation difference of 10%.

To visualize the episignature, methylation rates of the DMRs were extracted from the MethylDackel output. Hierarchical clustering and heatmap visualization were performed using the clustermap function implemented from the Seaborn Python package.[31]

Results

Clinical description

Here, we describe a cohort of 18 individuals, including one prenatal subject with *de novo* variants in *KDM2A*. An overview of the clinical data on all individuals is presented in Figure 1A and Table 1 (for a detailed phenotypic description, see the supplemental notes; Figure S1; Table S3).

All individuals, excluding the prenatal subject, exhibited developmental delay and/or intellectual disability, with the severity of developmental delay or intellectual disability ranging from learning disabilities to severe intellectual disability. The majority of individuals were affected by mild developmental delay/intellectual disability (11/17) or learning disabilities (2/17), while four individuals presented with severe developmental delay/intellectual disability. Microcephaly was observed in six individuals, including the prenatal subject. Three of the six individuals presented with primary microcephaly and three with secondary microcephaly. Autism was diagnosed in four individuals, while other behavioral abnormalities, such as attention-deficit hyperactivity disorder or aggressive behavior, were noted in seven individuals. Seizures occurred in five individuals, with onsets ranging from 4 months to 10 years. Seizure types included focal and generalized seizures as well as epileptic spasms. At the last assessment, two individuals continued to experience seizures, while three achieved seizure freedom. In the latter three, the anti-seizure medication was subsequently weaned off without relapse. Hypotonia was reported in four individuals. Cranial MRI was performed in 13 individuals, revealing normal results in seven. Minor and nonspecific abnormalities, such as cerebellar tonsillar ectopia and multiple white matter hyperintensities, were reported in four individuals. Of note, individual 4 did present with a malformation of cortical development (MCD) of the polymicrogyria spectrum. None of the individuals was reported to show signs of developmental regression. Another notable recurring phenotype in the cohort was growth abnormalities, including intrauterine growth restriction (IUGR) and/or short stature in thirteen individuals, including the prenatal subject. Eight individuals showed IUGR, and nine individuals developed short stature. Of note, four of these thirteen individuals presented with both IUGR and short stature later in life. Feeding difficulties were observed in six individuals, four of whom experienced these issues during the neonatal period, with two requiring tube feeding. Additionally, two individuals had persistent difficulties gaining weight later in life. The referring clinicians reported dysmorphic facial features in twelve individuals: epicanthus, upslanted palpebral fissures, thin vermillion of the upper and/or lower lips, and low-set ears emerged as recurrent descriptive features that each occurred in at least three individuals (Figure 1B; Table 1).

Genetic results

Exome and/or genome sequencing revealed *de novo* variants in *KDM2A* in 17 of the 18 affected individuals (Figure 1C). Individual 16 was identified using a singleton exome, but segregation analysis of the *KDM2A* variant in the parents was not available. We identified eleven distinct *de novo* missense variants and seven predicted loss-of-function (pLoF) variants. None of the variants are recurrent, and only two missense variants affect the same amino acid residue, Lys776. All of the identified variants are absent from gnomAD (v.4 dataset).[14] The eleven missense variants all affect moderate to highly conserved amino acid residues of *KDM2A*, but *in silico* prediction of potential deleteriousness varies within the cohort. Seven of the missense variants are predicted to be damaging by multiple algorithms, while results of the other four variants encompass mixed and benign predictions (Tables S2 and S5).

According to gnomAD, *KDM2A* is a gene with a significantly reduced number of pLoF as well as missense variants, indicating that there is a selective constraint on both types of variants in the general population that lacks severe, early-onset phenotypes such as developmental delay, intellectual disability, microcephaly, or short stature (LOEUF = 0.06; pLI = 1; o/e for missense variants = 0.49; Z score = 6.9).[14]

p.Pro235Leu variant alters nuclear localization and decreases KDM2A levels

KDM2A is primarily localized in the nucleus, and its interaction with unmethylated CpG DNA is crucial for

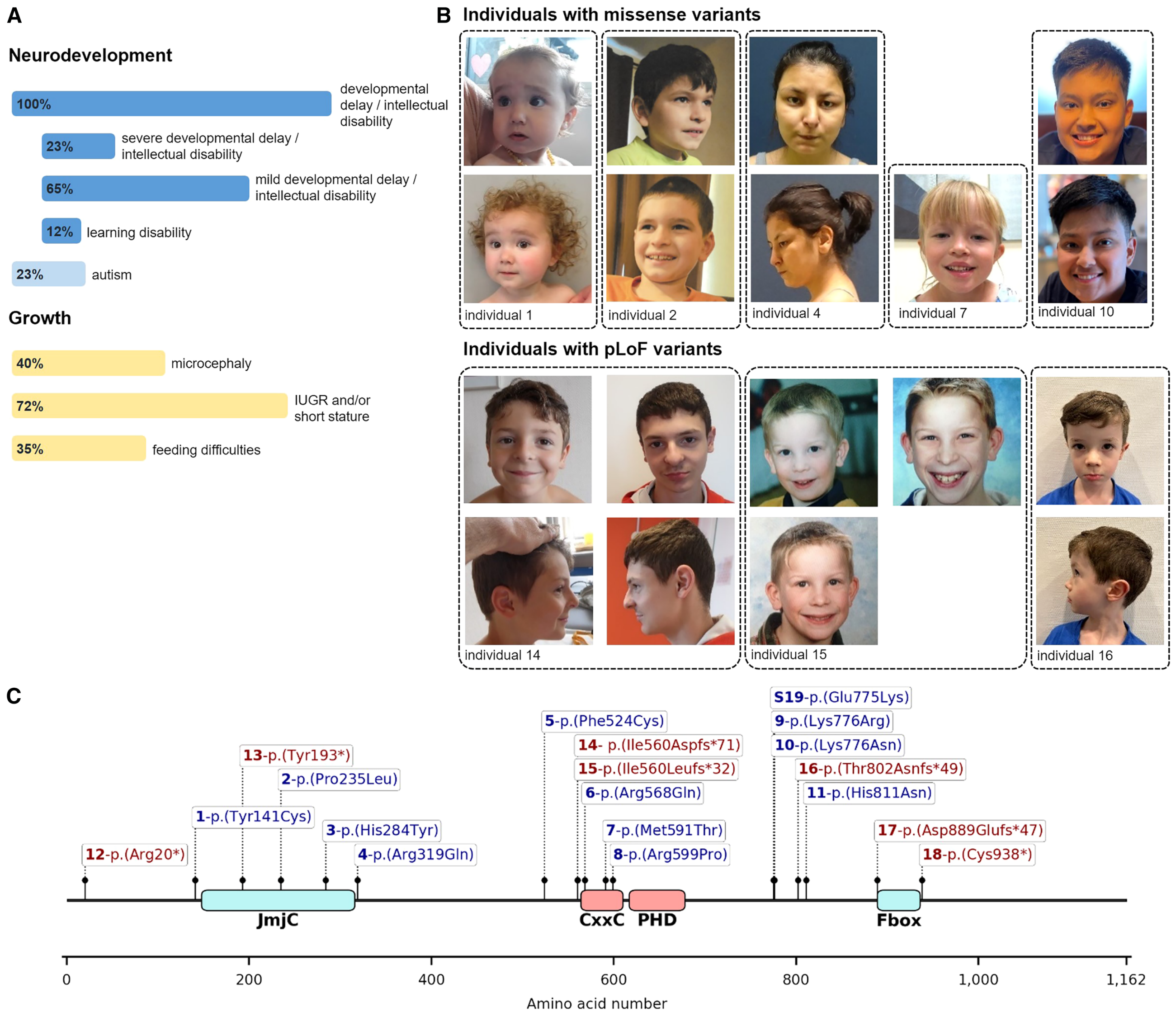

Figure 1. Prevalence of clinical findings and variant location on the protein level
(A) Core symptoms of the *KDM2A*-related neurodevelopmental disorder. IUGR, intrauterine growth restriction.
(B) Facial appearance of individuals at different ages that harbor missense variants or predicted loss-of-function variants in *KDM2A*. Epicanthus, upslanted palpebral fissures, thin upper and/or lower lips, and low-set ears were noted as recurrent dysmorphic facial features.
(C) Linear schematic representation of the KDM2A and location of the variants (GenBank: NM_012308.3). Bold numbers indicate individuals within the cohort. Blue variants represent missense variants, and red variants indicate predicted loss-of-function variants.

maintaining heterochromosomal homeostasis.[32,33] To investigate the impact of missense variants on the intracellular localization of KDM2A, we introduced HA-tagged WT and mutant forms (p.Pro235Leu, p.Tyr141Cys, and p.His811Asn) of KDM2A into HEK293T cells. While the expression of WT, p.Tyr141Cys, or p.His811Asn did not visibly affect the distribution and localization of KDM2A, the expression of the p.Pro235Leu variant led to the relocation of predominantly nuclear KDM2A to the cytoplasm (Figures 2A and 2B). Remarkably, the expression of the exogenous p.Pro235Leu variant also caused the cytoplasmic mislocalization of endogenous KDM2A (Figures 2A and 2C). To further illustrate the disruption in the localization of KDM2A variants, nucleo-cytoplasmic fractionation was performed in HEK293T cells expressing exogenous WT or mutant KDM2A, and both exogenous and endogenous KDM2A were probed using western blotting (Figure 2D). Interestingly, we observed a significant decrease in the nuclear/cytoplasmic ratio in both exogenous and endogenous KDM2A in the p.Pro235Leu variant. In contrast, no such alterations were detected in cells expressing p.Tyr141Cys or WT (Figures 2E and 2F), indicating a LoF in nuclear import for p.Pro235Leu.

Given the altered distribution of both exogenously expressed and endogenous KDM2A caused by p.Pro235Leu, we next sought to assess the impact of these variants on KDM2A levels. Specifically, we introduced WT,

Table 1. Clinical and genetic details of all affected individuals with causative variants in *KDM2A*

Ind.	Age[a] (sex)	Variant (GenBank: NM_012308.3)	Developmental delay/intellectual disability	Seizure type (age of onset) outcome	Microcephaly	Growth, feeding	Neurological findings, autism, behavior	Dysmorphic features	Further findings
1	5 y (F)	c.422A>G (p.Tyr141Cys), *de novo*	severe	–	+ (primary)	feeding difficulties (tube fed), poor weight gain, short stature	–	midface hypoplasia, bilateral epicanthus, widely spaced and deeply set eyes, low-set and posteriorly rotated ears, saggy cheeks	hypotonia, hyporeflexia
2	8 y (M)	c.704C>T (p.Pro235Leu), *de novo*	severe, no speech	–	–	–	autism	large pinnae, helical deformities, depressed nasal bridge, small jaw, short forehead	–
3	6 y (M)	c.850C>T (p.His284Tyr), *de novo*	mild	–	+ (secondary)	feeding difficulties, poor weight gain, short stature	ADHD	triangular face	–
4	25 y (F)	c.956G>A (p.Arg319Gln), *de novo*	severe, no speech	focal (10 y), not seizure free	–	short stature	dysarthria	upslanted palpebral fissures, narrow mouth with thin upper and lower vermillion of the lip, conical tapered fingers	MRI: polymicrogyria
5	13 y (F)	c.1571T>G (p.Phe524Cys), *de novo*	mild	generalized (1 y), seizure free	+ (primary)	IUGR, feeding difficulties	–	triangular face, pointed chin	–
6	10 y (M)	c.1703G>A (p.Arg568Gln), *de novo*	mild	–	+ (secondary)	IUGR, feeding difficulties (tube fed), short stature	behavioral abnormalities, ADHD	bilateral epicanthus	cryptorchidism
7	6 y (F)	c.1772T>C (p.Met591Thr), *de novo*	mild	–	–	feeding difficulties, short stature	autism, ADHD, aggressive behavior	frontal bossing, broad nasal bridge, bilateral epicanthus, high anterior hairline, temporal narrowing, low-set ears	hypotonia, decreased facial muscle tone
8	3 y (F)	c.1796G>C (p.Arg599Pro), *de novo*	mild	–	+ (secondary)	IUGR, feeding difficulties, low weight, short stature	–	protruding metopic suture, upslanted palpebral fissures, bilateral epicanthus, thin vermillion of the lower lip	delayed temporary teeth eruption
9	3 y (M)	c.2327A>G (p.Lys776Arg), *de novo*	learning disability	–	–	–	autism	–	–
10	13 y (M)	c.2328G>T (p.Lys776Asn), *de novo*	mild	generalized (9 y), not seizure free	–	–	ADHD, sensory/auditory processing disorder	upslanted palpebral fissures	–
11	14 y (M)	c.2431C>A (p.His811Asn), *de novo*	mild	epileptic spasms (5 m), seizure free	–	–	autism, Asperger-like	N/A	hypotonia

(*Continued on next page*)

Table 1. *Continued*

Ind.	Age[a] (sex)	Variant (GenBank: NM_012308.3)	Developmental delay/intellectual disability	Seizure type (age of onset) outcome	Microcephaly	Growth, feeding	Neurological findings, autism, behavior	Dysmorphic features	Further findings
12	5 y (M)	c.58C>T (p.Arg20*), *de novo*	mild	–	–	IUGR, short stature, delayed bone age	behavioral abnormalities	bilateral epicanthus, upslanted palpebral fissures, protruding ears	hypotonia
13	prenatal (F)	c.579C>G (p.Tyr193*), *de novo*	N/A	N/A	+ (primary)	IUGR	N/A	N/A	pregnancy terminated
14	13 y (M)	c.1676dup (p.Ile560Aspfs*71), *de novo*	learning disability	–	–	–	dyspraxia, ADHD, impulsivity, anxiety	high palate, thin vermillion of the upper lip	supernumerary nipple
15	25 y (M)	c.1677del (p.Ile560Leufs*32), *de novo*	mild, IQ 64	–	N/A	IUGR	–	micrognathia, mildly upslanted palpebral fissures	umbilical hernia; galbladder, pancreatic, and cardiac malformations; diabetes type 1 (all likely due to *GATA6* variant)[b]
16	6 y (M)	c.2404dup (p.Thr802Asnfs*49), heterozygous	mild	–	–	IUGR, short stature	–	micrognathia, thin upper vermillion of the lip, small midface	–
17	14 y (M)	c.2667del (p.Asp889Glufs*47), *de novo*	severe	focal dyscognitive (4 y), seizure free	N/A	short stature	ADHD, anxiety	–	inverted nipples, bilateral club foot
18	11 y (M)	c.2809_2812dup (p.Cys938*), *de novo*	mild	–	–	IUGR	anxiety	low-set ears, thin upper vermillion of the lip with long philtrum, retrognathia, long and narrow nose	–

ADHD, attention-deficit hyperactivity disorder; DD, developmental delay; F, female; Ind., individual; IUGR, intrauterine growth restriction; M, male; m, months; N/A, not available; y, years; –, not reported. Further clinical details are provided in Table S3.

[a]Age at last assessment.

[b]See Table S3 and supplemental notes.

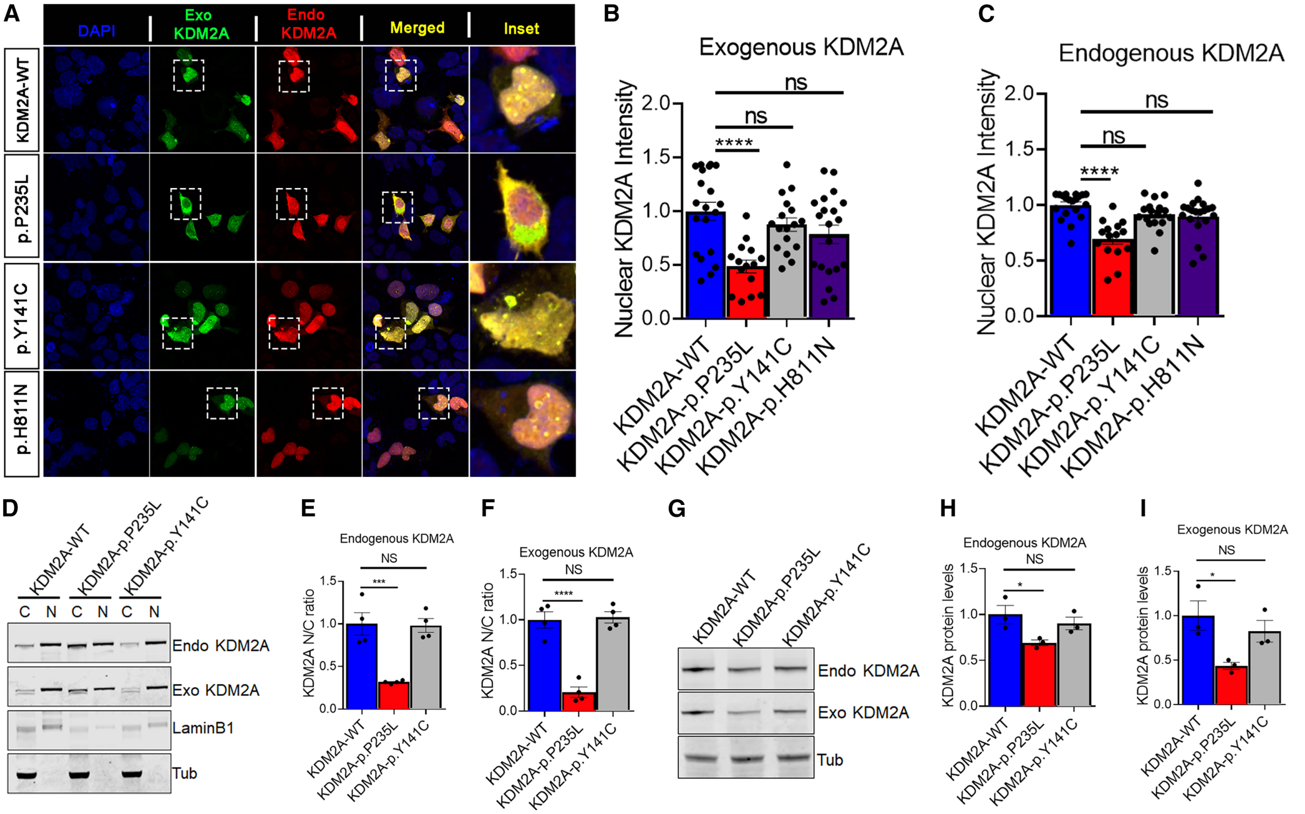

Figure 2. KDM2A variants alter the subcellular distribution of KDM2A in mammalian cells

(A) Representative immunofluorescence images of human embryonic kidney 293T cells (HEK293T cells) transfected with HA-tagged wild-type KDM2A (KDM2A-WT) or variants (P235L, Y141C, or H811N) stained for exogenous (green) and endogenous (red) KDM2A. DAPI was used to label nuclei.

(B) Exogenous KDM2A nuclear intensity quantification showed that P235L (****$p < 0.0001$), but not Y141C or H811N, showed significantly decreased nuclear intensity compared to exogenous KDM2A-WT ($n = 14$–20 cells).

(C) Endogenous KDM2A nuclear intensity quantification showed that P235L (****$p < 0.0001$), but not Y141C or H811N, produced a significant reduction in nuclear intensity of endogenous KDM2A as compared to KDM2A-WT ($n = 14$–20 cells).

(D) Western blots of cytoplasmic (C) and nuclear (N) fractions from HEK cells transfected with KDM2A-WT, P235L, and Y141C variants probed for exogenous KDM2A (anti-HA), endogenous KDM2A (anti-KDM2A), nuclear membrane marker (laminB1), and tubulin.

(E) Nuclear-cytoplasmic (N/C) ratio quantification of endogenous KDM2A ($n = 3$ blots, ***$p < 0.001$).

(F) N/C ratio quantification of exogenous KDM2A ($n = 3$ blots, ***$p < 0.001$).

(G) Western blots of HEK cells transfected with KDM2A-WT, P235L, and Y141C stained for endogenous KDM2A (anti-KDM2A) and exogenous KDM2A (anti-HA). Tubulin was used as the loading control.

(H and I) Western blot quantification of endogenous KDM2A (H) and exogenous KDM2A (I) in HEK cells (*$p < 0.05$, $n = 3$).

One-way ANOVA was performed to determine the significance in (B), (C), (E), (F), (H), and (I). All quantifications represent the mean ± SEM.

p.Pro235Leu, and p.Tyr141Cys plasmids into HEK cells and examined both exogenous and endogenous KDM2A through western blotting (Figure 2G). In the WT and p.Tyr141Cys conditions, there was no notable change in the expression of endogenous *KDM2A*. However, the p.Pro235Leu variant significantly decreased the levels of both exogenously expressed and endogenous KDM2A (Figures 2G–2I). This suggests that the p.Pro235Leu variants likely disrupt the stability of KDM2A.

To investigate the mechanisms underlying the reduced KDM2A levels, we compared the stability of KDM2A between the WT and p.Pro235Leu mutant protein. We performed WB analysis on HEK cells transfected with either the WT or p.Pro235Leu plasmid. Protein lysates were harvested at 0, 6, 12, 24, and 48 h after CHX treatment (Figures 3A and 3B). The WB analysis revealed that KDM2A is less stable in the p.Pro235Leu variant-expressing cells, with a half-life ($t_{1/2}$) of 4.35 h compared to a $t_{1/2}$ of 7.04 h in WT-expressing cells. This suggests that the differential reduction of KDM2A in p.Pro235Leu variants is due to differences in the stability of KDM2A.

KDM2A variants cause eye degeneration, motor defects, and reduce lifespan in a *Drosophila* model

We next investigated the *in vivo* effects of missense variants in the *Drosophila* model system. To achieve this, we generated transgenic *Drosophila* expressing human *KDM2A*-WT and missense variants (p.Pro235Leu, p.Tyr 141Cys, and p.His811Asn) through PhiC31 integrase-mediated site-specific insertion of a single copy of human

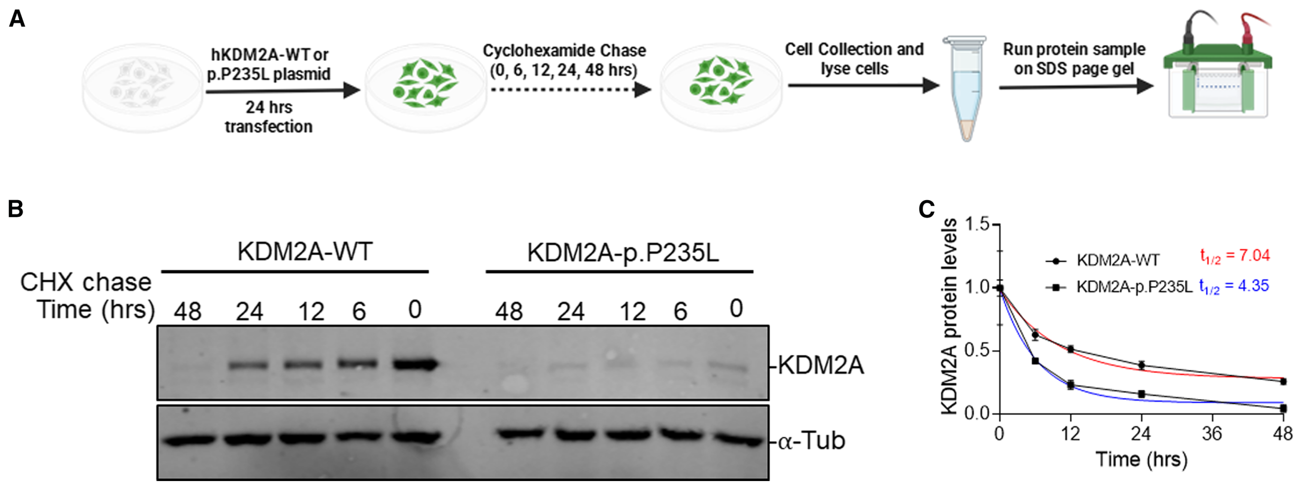

Figure 3. The p.P235L variant alters the stability of the KDM2A
(A) Schematic of the cycloheximide (CHX) experiment conducted in HEK293T cells.
(B) Representative western blot showing KDM2A levels (anti-HA) in HEK293T cells transfected with either WT or P235L KDM2A plasmid at 0, 6, 12, 24, and 48 h following CHX addition. Tubulin was used as a normalization control.
(C) Quantification of the degradation rate and half-life ($t_{1/2}$) of KDM2A after CHX treatment revealed an accelerated depletion of KDM2A in P235L-expressing cells compared to exogenous WT expression (nonlinear regression: one-phase decay, $n = 3$).

KDM2A. The expression of these transgenes in *Drosophila* using the eye tissue-targeting GMR-gl4 driver revealed a variant-dependent rough eye phenotype, with the p.Pro235Leu variant exhibiting more pronounced effects (Figures 4A and 4B). The expression of WT showed a very mild phenotype. Likewise, in line with observations from HEK293T cells, the expression of the p.Pro235Leu variant in *Drosophila* eyes resulted in a notable decrease in KDM2A levels (Figures 4C and 4D).

To assess whether ectopic expression of *KDM2A* variants could cause motor deficits, we evaluated locomotion in 20-day-old *Drosophila*. We expressed these variants pan-neuronally with the Elav-gal4 driver. While expression of *KDM2A*-WT mildly but significantly reduced climbing velocity, a more profound decrease in climbing ability was observed in *Drosophila* expressing the *KDM2A* mutants, particularly in the p.Pro235Leu variant (Figure 4E). To further determine the toxic impact of KDM2A, we assessed the effects of *KDM2A* variants on survival. Pan-neuronal expression of WT or mutant *KDM2A* significantly reduced survival compared to the luciferase control. However, the p.Pro235Leu variant led to a significantly shorter lifespan compared to both *KDM2A*-WT and the other variants (Figure 4F), suggesting that the cytoplasmic p.Pro235Leu variant confers a toxic gain of function in addition to loss of nuclear activity.

Given our data showing that the p.Pro235Leu variant disrupts normal subcellular distribution and potentially impairs the function of endogenous KDM2A, we further investigated the toxicity of *KDM2A* variants in a *Drosophila* model where endogenous *Kdm2* (the single *Drosophila* ortholog of human *KDM2A* and *KDM2B* [MIM: 609078]) is either knocked down or knocked out. First, we knocked down *Kdm2* in *Drosophila* eyes while expressing human WT and mutant (p.Pro235Leu, p.Tyr141Cys, and p.His811Asn) KDM2A. Knockdown (KD) of endogenous *Kdm2* in *Drosophila* eyes did not result in any overt degeneration, and expression of human KDM2A-WT in the Kdm2 KD background produced only a mild phenotype. In contrast, expression of the human *KDM2A* variants (p.Pro235Leu, p.Tyr141Cys, and p.His811Asn)—most prominently p.Pro235Leu—in the *Kdm2* KD background significantly exacerbated the degenerative eye phenotype compared to their respective controls (Figures 5A and 5B). These results confirm that p.Pro235Leu's effects cannot be explained by the loss of *Kdm2* alone and instead reflect a toxic gain-of-function mechanism.

We further utilized the Trojan-MiMIC Gal4 driver system, which employs RMCE of a Mi{MIC} insertion, resulting in the expression of GAL4 under the control of *Kdm2* regulatory sequences.[27,34] Consequently, *Drosophila Kdm2* is knocked out in the Trojan-Gal4Kdm2 lines, allowing for a robust analysis of human *KDM2A* variants in a clean genetic background. We assessed motor function and lifespan in adult *Drosophila* expressing human *KDM2A*-WT and its variants in the specific cell types where endogenous *Drosophila Kdm2* is normally expressed. The lack of phenotype in *Kdm2* knockout (KO) animals suggests that the loss of *Kdm2* in adults is not critical under the conditions tested (Figures 5C and 5D). Similarly, we found that expression of human *KDM2A*-WT did not affect motor function or lifespan compared to Kdm2 KO flies. However, the human *KDM2A* variants (p.Pro235Leu, p.Tyr141Cys, and p.His811Asn) resulted in significantly worse motor function and survival compared to both the KO and the human WT scenarios (Figures 5C–5E). Altogether, these findings suggest that these variants in *KDM2A* are toxic, possibly due to a gain-of-function and/or a LoF mechanism.

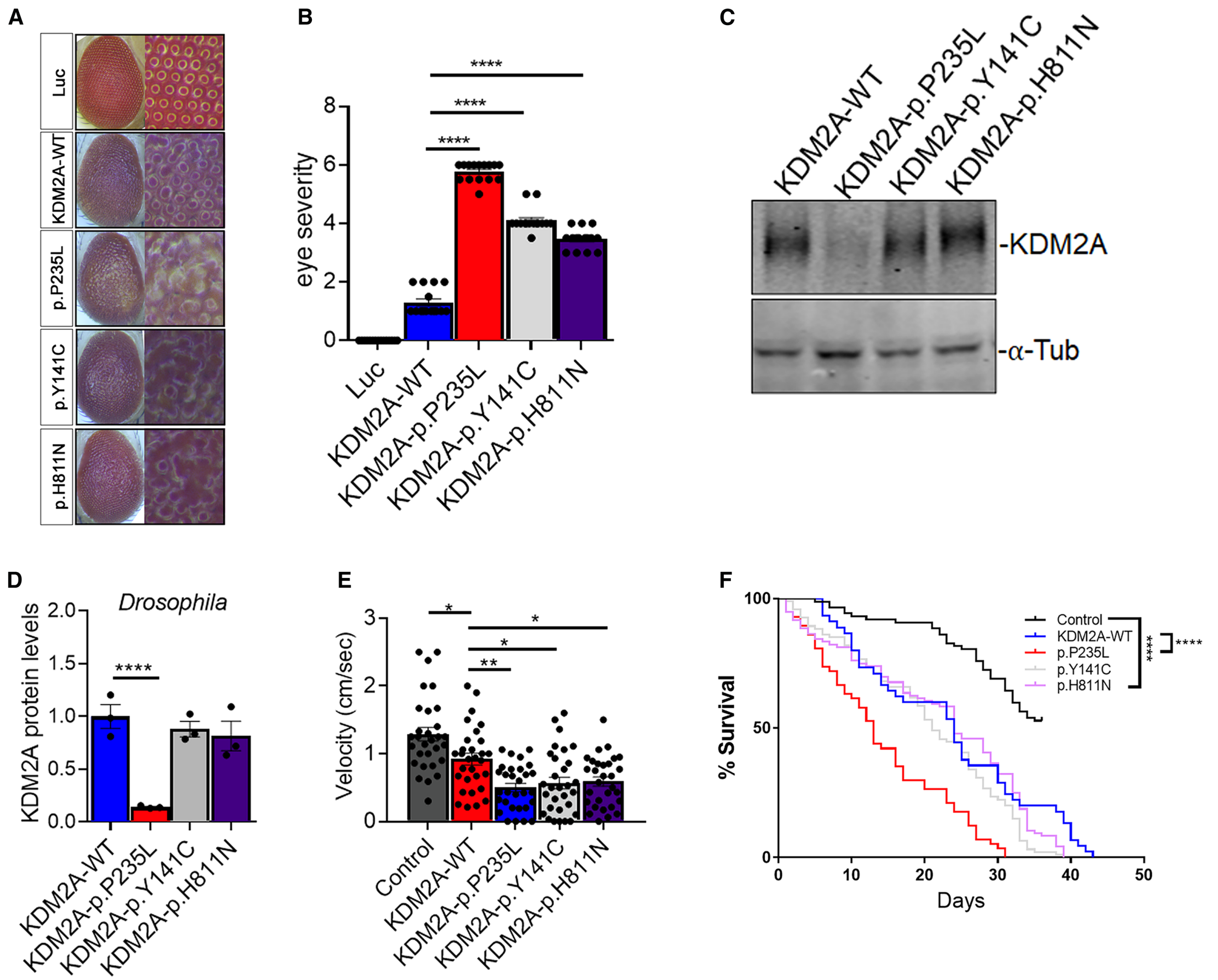

Figure 4. A *Drosophila* model expressing *KDM2A* variants displays differential toxicity *in vivo*

(A) Representative panel of adult Drosophila eyes expressing KDM2A-WT, P235L, Y141C, H811N, or luciferase (luc) control.

(B) Quantification of eye degeneration severity demonstrated that KDM2A variants significantly enhance toxicity as compared to wild-type KDM2A (****$p < 0.0001$, $n = 15$–20).

(C) Western blots of *Drosophila* expressing KDM2A (WT, P235L, Y141C, and H811N) in the eye (GMR-gal4) stained with anti-KDM2A and anti-tubulin.

(D) Western blot quantification of KDM2A in *Drosophila* showed that the P235L, but not the Y141C or H811N, variants had a significantly reduced protein level compared to KDM2A-WT (****$p < 0.0001$, $n = 3$).

(E) Quantification of climbing velocity (cm/s) in *Drosophila* expressing KDM2A-WT, P235L, Y141C, and H811N pan-neuronally (Elav-gal4) compared to luc control or wild-type KDM2A ($n = 3$ trials, 10 animals per trial, **$p < 0.01$ and *$p < 0.05$).

(F) Kaplan-Meier survival curve of *Drosophila* expressing KDM2A-WT, P235L, Y141C, and H811N in neurons when each is individually compared with luc control ($n = 50$–80, ****$p < 0.0001$).

One-way ANOVA was performed in (B)–(D), while log rank with Grehan-Breslow-Wilcoxon tests were performed to determine the significance in (F). All quantifications represent the mean ± SEM.

Methylome analysis

As KDM2A is a component of the epigenetic machinery, it was hypothesized that disruption of KDM2A function by *de novo* variants would cause an abnormal methylation pattern in peripheral blood as a *de facto* functional readout. Analysis of enzymatic-methylation sequencing data of thirteen individuals with pLoF variants and missense variants compared to a control group identified 817 DMRs. After applying stringent filtering criteria (*p*-adj < 0.01 and min. mean methylation difference > 10%), we defined a first episignature of the *KDM2A* cases. This signature includes 598 DMRs (585 hypermethylated and 13 hypomethylated regions) with a median methylation difference of 16.2% and a median length of ~226 nt/20 CpGs (Table S8). Hierarchical clustering of affected individuals and controls revealed a distinct *KDM2A* cluster with individuals harboring frameshift variants (individuals 14, 15, 16, and 18) and missense variants (individuals 2, 4, 7, and 8) grouping together within this *KDM2A* cluster (Figure 6). Data of four individuals (individuals 1, 5, 9,

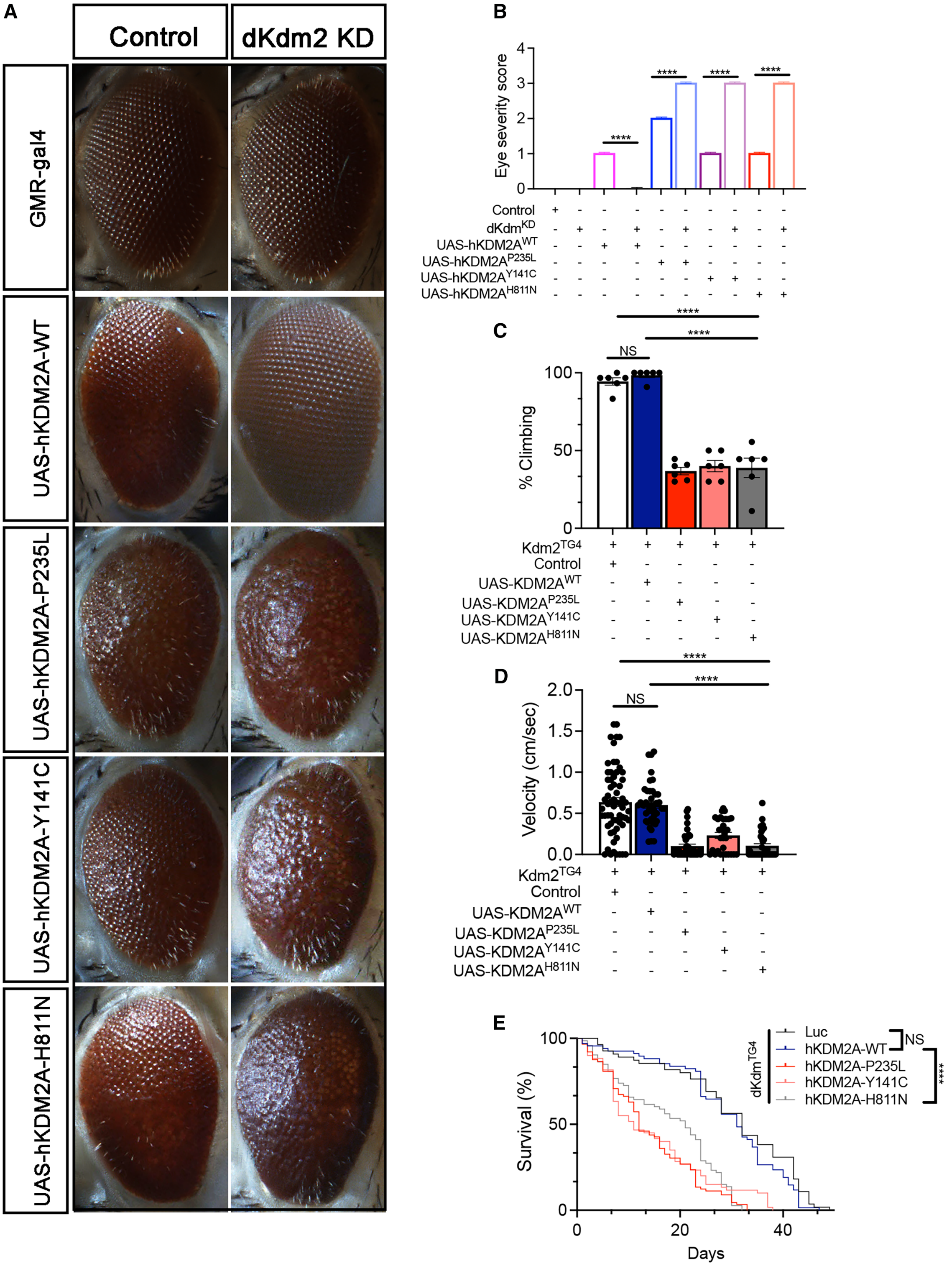

Figure 5. Expression of human *KDM2A* variants in a *Drosophila Kdm2* knockdown or knockout model increases toxicity
(A) Images of *Drosophila* eyes expressing human WT or mutant KDM2A in the context of either endogenous Kdm2 or RNAi-mediated Kdm2 knockdown (KD).
(B) Quantification shows that human KDM2A (hKDM2A) variants significantly increase eye degeneration severity in the Kdm2 KD background compared to controls with endogenous Kdm2 (****$p < 0.0001$, $n = 20$). Notably, neither hKDM2A-WT with endogenous Kdm2 KD nor Kdm2 KD alone caused overt eye degeneration.
(C–E) Quantification analyses revealed a significant reduction in the percentage of *Drosophila* capable of climbing (****$p < 0.0001$, $n = 6$ trials/10 flies per trial) (C), climbing velocity (****$p < 0.0001$, $n = 3$ trials/15–20 flies per trial) (D), and lifespan (Kaplan-Meier survival

(legend continued on next page)

and 11) with missense variants were shown to group outside the bigger *KDM2A* cluster and within the controls. In addition, the methylation analysis of individual S19, where the *de novo* origin of the missense variant could not be tested in the parents, also showed a methylation result comparable to the control group.

Discussion

In this study, we provide a detailed description of 18 individuals with heterozygous variants in *KDM2A*, including 17 with confirmed *de novo* status, all presenting with a syndromic NDD.

The affected individuals show an overlapping phenotype of developmental delay/intellectual disability and several recurrent symptoms of note: growth abnormalities (including IUGR and short stature), microcephaly, and feeding difficulties that are present in 35%–72% of individuals. Developmental delay/intellectual disability and growth abnormalities are recurrent symptoms among the Mendelian disorders of the epigenetic machinery,[3] underscoring that the *KDM2A*-related rare disease delineated here shows a clinical overlap. Many of these disorders also present with a recognizable facial gestalt and include some of the classic dysmorphic syndromes, such as Kabuki syndrome (e.g., *KMT2D*; MIM: 147920 and MIM: 602113) or Rubinstein-Taybi syndrome (e.g., *CREBBP*; MIM: 180849 and MIM: 600140). The affected individuals in the *KDM2A* cohort also showed facial dysmorphism, highlighted by recurrent observations of epicanthus, upslanted palpebral fissures, thin upper and/or lower lips, and low-set ears as potentially defining features (Figure 1B). Deep phenotyping of facial images of affected individuals with the help of facial recognition tools could help to detect this potential typical facial gestalt of the *KDM2A*-related syndromic NDD in routine clinical genetic care.[35,36] An MCD, such as the polymicrogyria observed in individual 4, is not a phenotype seen in other disorders of the epigenetic machinery. The trio exome analysis of this individual did not reveal any additional causative variant(s) for this phenotype in genes associated with an MCD nor additional variants in other candidate genes.[37] It remains, therefore, unclear whether polymicrogyria is part of the phenotypic spectrum of the *KDM2A*-related disorder or if another, undetected genetic cause is involved. Of note, feeding difficulties were only present in individuals with *de novo* missense variants in *KDM2A* and were absent in individuals with pLoF variants, suggesting a potential genotype-phenotype correlation. Similarly, microcephaly was observed exclusively in individuals with missense variants, with the exception of the prenatal subject carrying a pLoF variant. Other recurrent phenotypes, however, did not show any potential genotype-phenotype correlations, as they were observed in individuals who harbor missense variants and individuals with pLoF variants.

As mentioned previously, *KDM2A* is a gene with a significantly reduced number of pLoF and missense variants. Among KDM genes, *KDM2A* is in fact the most highly constrained gene with respect to both missense and pLOF variants (see constraint score landscape of KDMs in Figure S2 as well as Table S6). This high constraint compared to genes within the group of KDMs, where variants are known to cause a rare disease, strengthens our case that rare variants in *KDM2A* are also causative for a Mendelian condition.

It is now also possible to evaluate prior assessments on what phenotypes could be caused by variants in candidate genes such as *KDM2A*. In a recent work by Dhindsa et al., using a machine learning approach based on gene constraint, expression, and many other gene-level annotations, *KDM2A* was predicted to cause an autosomal-dominant phenotype of developmental delay, developmental epileptic encephalopathy, and autism in the 99.5th percentile or higher for each phenotype individually.[38] This prior assessment aligns with our phenotypic findings, including that both autism and epilepsy are important parts of the phenotypic spectrum of the *KDM2A*-related syndromic disorder, although only five affected individuals presented with seizures, and four had a diagnosis of autism.

The identified *de novo* missense variants in *KDM2A* are located in or around the JmjC domain, essential for demethylation activity, and the CxxC domain (a zinc finger [ZF]), essential for DNA binding.[1,39] In *KDM2B*, *de novo* missense variants are also preferably located in or around these two domains specifically (Figure S3).

In this study, we investigated the consequences of missense variants in *KDM2A* on the subcellular distribution of KDM2A and toxicity. Previous studies demonstrated that KDM2A primarily localizes to the nucleus, where it binds to unmethylated CpG DNA via the ZF-CxxC domain.[32] Consistent with these findings, *KDM2A*-WT expressed in HEK293T cells exhibited predominantly nuclear localization. In contrast, our observations revealed distinct localization patterns for the p.Tyr141Cys and p.His811Asn variants, which displayed primarily nuclear with occasional cytoplasmic aggregation, while the p.Pro235Leu variant showed significant nuclear exclusion and cytoplasmic accumulation (Figure 2). Moreover, the endogenously expressed KDM2A undergoes cytoplasmic redistribution in the presence of the exogenously expressed p.Pro235Leu variant. This observation was further supported by nuclear-cytoplasmic fractionation experiments, which

curve, ****$p < 0.0001$, $n = 80–90$ flies) (E) in Kdm2 KO *Drosophila* expressing hKDM2A variants (P235L, Y141C, and H811N) using the Trojan-Gal4 system, compared to hKDM2A-WT with endogenous Kdm2 KD or KO flies alone.
Statistical significance was determined by one-way ANOVA for (B)–(D) and by log rank with Grehan-Breslow-Wilcoxon tests for (E). All data are presented as the mean ± SEM.

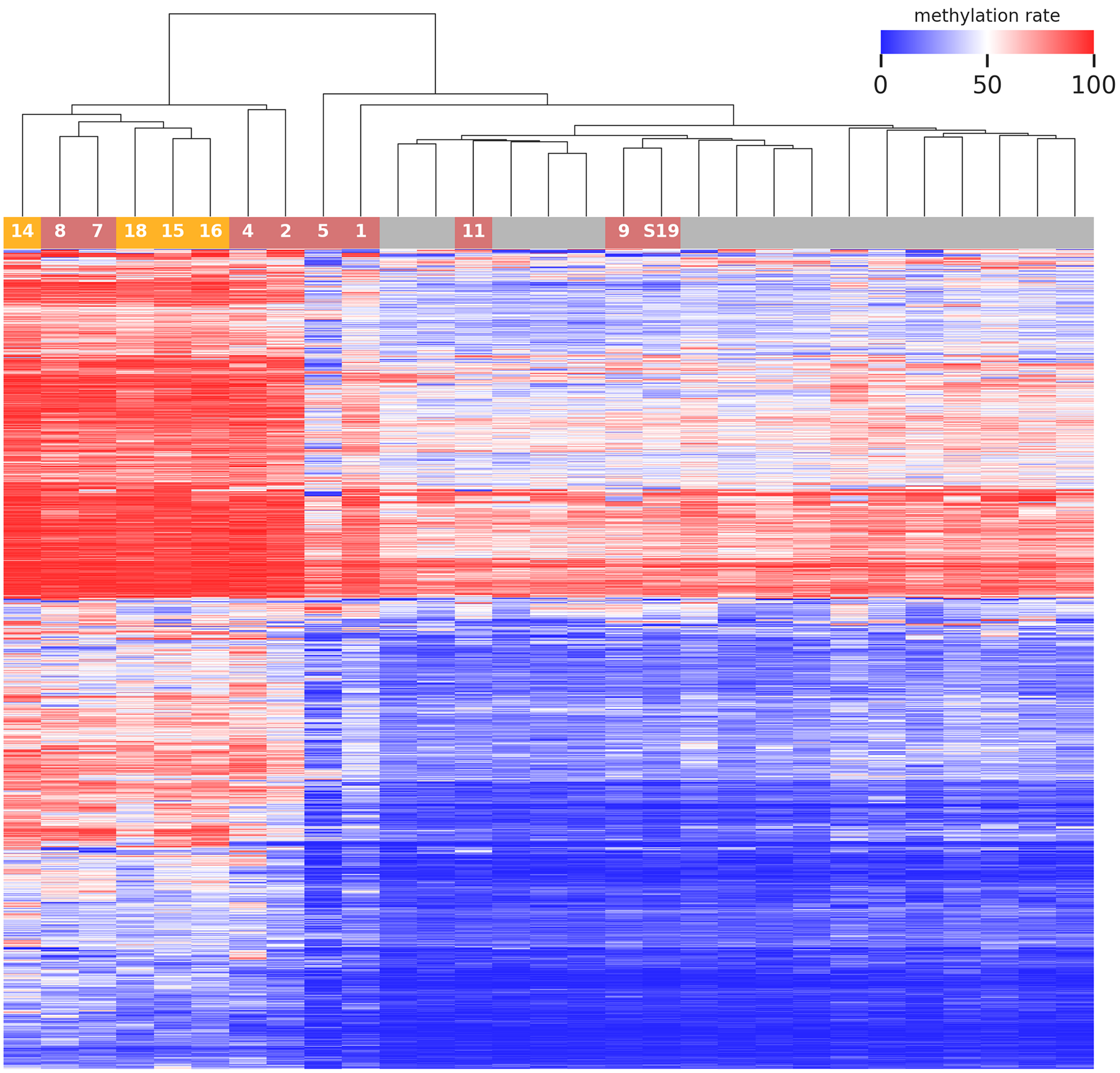

Figure 6. Episignature of pLoF and missense variants in *KDM2A*
Heatmap displays hierarchical clustering of selected CpG sites of the episignature. Columns represent probes (gray: control probes; yellow: *KDM2A* pLoF variants; light red: *KDM2A* missense variants; number represents individual in the cohort). Rows represent CpG sites. Color represents methylation, ranging from dark blue (no methylation) to dark red (full methylation). A distinct separation between control samples and those from individuals with variants in *KMD2A* is observed. For individual S19, there is insufficient evidence for the diagnosis of a *KDM2A*-related syndromic neurodevelopmental disorder (a *de novo* origin of the variant could not be proven, and methylation analysis does not support a diagnosis either; see also the supplemental notes and Table S4).

consistently demonstrated predominant cytoplasmic localization of the p.Pro235Leu variant, suggesting a potential disruption in nuclear import. The loss of nuclear KDM2A may impair its ability to repress the transcription of centromeric satellite repeats and maintaining a heterochromatic state.[33] Additionally, nuclear redistribution of KDM2A to the cytoplasm may also impair its ability to bind and regulate the stability of non-phosphorylated nuclear β-catenin, thereby potentially affecting the Wnt/β-catenin signaling pathway.[40] Alternatively, cytoplasmic accumulation of KDM2A may confer a toxic gain of function. However, further investigations are required to elucidate the precise underlying mechanism. Moreover, our data revealed that the forced expression of the p.Pro235Leu variant is accompanied by a significant reduction in both endogenous and exogenously expressed KDM2A levels, indicating compromised protein stability. This was confirmed through CHX chase experiments, which showed that the p.Pro235Leu variant reduces the $t_{1/2}$ of KDM2A, suggesting that this variant negatively impacts protein stability. Given its location within the JmjC catalytic domain, which is critical for KDM2A's histone demethylase activity, the p.Pro235-Leu substitution may alter enzymatic function, protein stability, or cofactor binding. This is consistent with our experimental observations of altered protein stability and mislocalization.

In addition to markedly reducing protein stability and its location within *KDM2A*'s JmjC catalytic domain, the p.Pro235Leu substitution likely perturbs the local folding of the catalytic core in a manner that secondarily disrupts

chromatin engagement and nuclear retention. Structural and functional studies indicate that the JmjC module is upstream of KDM2A's chromatin-targeting ZF-CxxC domain and that interdomain integrity is important for high-affinity CpG binding and stable nuclear association.[33,41,42] We therefore infer that substitution of the rigid proline at 235 with a more flexible hydrophobic leucine destabilizes the JmjC fold, producing a conformational change that probably (1) weakens cooperative interactions with the ZF-CxxC chromatin anchor, (2) exposes sequences that favors cytoplasmic routing (for example, cryptic nuclear export signals or degron motifs), or (3) increases susceptibility to post-translational modifications that promote ubiquitin-dependent nuclear export and clearance. By contrast, p.Tyr141Cys resides in the JmjN subdomain of the catalytic module, and p.His811Asn maps to the C-terminal F box region; neither substitution directly perturbs the ZF-CxxC chromatin-binding fold or characterized nuclear localization elements, which plausibly explains why these variants largely retain nuclear localization and do not recapitulate the mislocalization observed for p.Pro235Leu. Thus, the striking cytoplasmic mislocalization of p.Pro235Leu most likely reflects a domain-specific structural perturbation of the catalytic core that compromises KDM2A's chromatin tethering and nuclear retention rather than a global unfolding of the protein, highlighting a mechanistic distinction between disruptions of KDM2A's enzymatic architecture versus its chromatin-targeting module.

The observed redistribution of KDM2A from the nucleus to the cytoplasm may exert toxic effects on cells. Similar mechanisms have been reported in amyotrophic lateral sclerosis and frontotemporal dementia (ALS/FTD), where pathogenic variants in proteins such as FUS and TDP-43 lead to nuclear clearance, contributing to cellular toxicity in various animal models, including *Drosophila*.[23,43] To test whether *KDM2A* variants induce toxicity in an *in vivo* system, we generated *Drosophila* models expressing three missense variants (p.Tyr141Cys, p.Pro235Leu, and p.His811Asn). Ectopic expression of *KDM2A* in neuronal cells led to eye degeneration, motor deficits, and reduced survival (Figure 2), with the p.Pro235Leu variant exhibiting the highest toxicity, consistent with our *in vitro* findings. While *KDM2A*-WT caused mild toxicity, this parallels observations from FUS expression studies in *Drosophila*.[23] Our data suggest that both loss of nuclear function and a potential gain-of-function effect in the cytoplasm contribute to the observed cellular toxicity, particularly evident in the p.Pro235Leu variant. Further research is needed to examine the exact mechanisms underlying *KDM2A* variant-mediated toxicity. Our *in vivo Drosophila* data using a *Kdm* KO or KD system suggested a possible gain of function of *KDM2A* variants. Our data showed that the expression of human WT *KDM2A* in the KD or KO background did not affect eye severity, motor function, or lifespan, which were similar to those observed in KD or KO *Kdm* flies. In contrast, expression of *KDM2A* variants resulted in significantly worse phenotypes compared to both KO flies and those expressing human *KDM2A*-WT. These findings suggest that variants in *KDM2A* may confer a new or enhanced toxic effect, characteristic of gain-of-function variants. However, a limitation of our study is the absence of direct functional analysis of other reported *KDM2A* variants, particularly the nonsense variants p.Arg20* (c.58C>T) and p.Tyr193* (c.579C>G). These premature stop codons likely result in truncated proteins lacking critical functional domains, such as the JmjC catalytic domain and the CxxC ZF, potentially leading to complete loss of function through nonsense-mediated mRNA decay or dominant-negative effects. While our mechanistic investigation focused on the p.Pro235Leu missense variant, which we show alters protein stability, localization, and potentially chromatin interactions, evaluating the functional consequences of the truncating variants will be essential to determine whether they act through similar or distinct pathogenic pathways. Future studies incorporating these variants will provide a more comprehensive understanding of *KDM2A*-related disease mechanisms.

We analyzed blood-derived DNA and detected a *KDM2A*-related episignature, further underscoring a shared pathomechanism among affected individuals with missense and pLoF variants. In contrast, methylation analysis of several individuals with missense variants showed results comparable to controls (individuals 1, 5, 9, and 11). All four variants are of *de novo* origin, the clinical phenotype of all individuals fits the described spectrum, and functional analysis of, e.g., the missense variant of individual 11 (p.His811Asn) showed a toxic effect in the *Drosophila* model. Thus, the diagnosis of a *KDM2A*-related syndromic NDD is warranted in all four individuals. This reinforces the point that the lack of an aberrant methylation profile does not rule out a specific diagnosis, as laid out in a recent clinical utility recommendation for episignature testing.[44] However, in the case of individual S19, where (1) the *de novo* origin of the underlying missense variant could not be proven due to the individuals' adoption at age 2 months and (2) a lack of a *KDM2A*-associated methylation pattern, a diagnosis of a *KDM2A*-related NDD is not warranted, and thus, we did not include this individual in the main cohort (see supplemental notes and Table S4). In the future, with further refinement, this episignature could serve as a valuable tool for interpreting variants of unknown significance and aid in the diagnosis of affected individuals.

In summary, we use different lines of clinical, genetic, functional, and epigenetic evidence to firmly establish *de novo* variants in *KDM2A* as the cause of a syndromic NDD.

Data and code availability

All identified variants in *KDM2A* have been uploaded to ClinVar with the following accession numbers: VCV003778797, VCV003778794, VCV003778798, VCV003778805, VCV003377192, VCV003778811, VCV003778812, VCV003778799, VCV

003778800, VCV003778813, VCV003359230, VCV003778814, VCV003778815, VCV003778795, VCV003778801, VCV003778796, VCV003778802, and VCV003778803 (https://www.ncbi.nlm.nih.gov/clinvar/submitters/506086/).

Pipeline for enzymatic-methylation sequencing data processing is available at https://github.com/StephanHolgerD/methylmappdedupmethyldackelsnakemake.

Consortia

The members of the Undiagnosed Diseases Network are Maria T. Acosta, David R. Adams, Ben Afzali, Ali Al-Beshri, Eric Allenspach, Aimee Allworth, Raquel L. Alvarez, Justin Alvey, Ashley Andrews, Euan A. Ashley, Carlos A. Bacino, Guney Bademci, Ashok Balasubramanyam, Dustin Baldridge, Jim Bale, Michael Bamshad, Deborah Barbouth, Pinar Bayrak-Toydemir, Anita Beck, Alan H. Beggs, Edward Behrens, Gill Bejerano, Hugo J. Bellen, Jimmy Bennett, Jonathan A. Bernstein, Gerard T. Berry, Anna Bican, Stephanie Bivona, Elizabeth Blue, John Bohnsack, Devon Bonner, Nicholas Borja, Lorenzo Botto, Lauren C. Briere, Elizabeth A. Burke, Lindsay C. Burrage, Manish J. Butte, Peter Byers, William E. Byrd, Kaitlin Callaway, John Carey, George Carvalho, Thomas Cassini, Sirisak Chanprasert, Hsiao-Tuan Chao, Ivan Chinn, Gary D. Clark, Terra R. Coakley, Laurel A. Cobban, Joy D. Cogan, Matthew Coggins, F. Sessions Cole, Brian Corner, Rosario I. Corona, William J. Craigen, Andrew B. Crouse, Vishnu Cuddapah, Precilla D'Souza, Hongzheng Dai, Kahlen Darr, Surendra Dasari, Joie Davis, Margaret Delgado, Esteban C. Dell'Angelica, Katrina Dipple, Daniel Doherty, Naghmeh Dorrani, Jessica Douglas, Emilie D. Douine, Dawn Earl, Lisa T. Emrick, Christine M. Eng, Cecilia Esteves, Kimberly Ezell, Elizabeth L. Fieg, Paul G. Fisher, Brent L. Fogel, Jiayu Fu, William A. Gahl, Rebecca Ganetzky, Emily Glanton, Ian Glass, Page C. Goddard, Joanna M. Gonzalez, Andrea Gropman, Meghan C. Halley, Rizwan Hamid, Neal Hanchard, Kelly Hassey, Nichole Hayes, Frances High, Anne Hing, Fuki M. Hisama, Ingrid A. Holm, Jason Hom, Martha Horike-Pyne, Alden Huang, Yan Huang, Anna Hurst, Wendy Introne, Gail P. Jarvik, Suman Jayadev, Orpa Jean-Marie, Vaidehi Jobanputra, Oguz Kanca, Yigit Karasozen, Shamika Ketkar, Dana Kiley, Gonench Kilich, Eric Klee, Shilpa N. Kobren, Isaac S. Kohane, Jennefer N. Kohler, Bruce Korf, Susan Korrick, Deborah Krakow, Elijah Kravets, Seema R. Lalani, Christina Lam, Brendan C. Lanpher, Ian R. Lanza, Kumarie Latchman, Kimberly LeBlanc, Brendan H. Lee, Kathleen A. Leppig, Richard A. Lewis, Pengfei Liu, Nicola Longo, Joseph Loscalzo, Richard L. Maas, Ellen F. Macnamara, Calum A. MacRae, Valerie V. Maduro, AudreyStephannie Maghiro, Rachel Mahoney, May Christine V. Malicdan, Rong Mao, Ronit Marom, Gabor Marth, Beth A. Martin, Martin G. Martin, Julian A. Martínez-Agosto, Shruti Marwaha, Allyn McConkie-Rosell, Ashley McMinn, Matthew Might, Mohamad Mikati, Danny Miller, Ghayda Mirzaa, Breanna Mitchell, Paolo Moretti, Marie Morimoto, John J. Mulvihill, Lindsay Mulvihill, Mariko Nakano-Okuno, Stanley F. Nelson, Serena Neumann, Dargie Nitsuh, Donna Novacic, Devin Oglesbee, James P. Orengo, Laura Pace, Stephen Pak, J. Carl Pallais, Neil H. Parker, LéShon Peart, Leoyklang Petcharet, John A. Phillips III, Filippo Pinto e Vairo, Jennifer E. Posey, Lorraine Potocki, Barbara N. Pusey Swerdzewski, Aaron Quinlan, Daniel J. Rader, Ramakrishnan Rajagopalan, Deepak A. Rao, Anna Raper, Wendy Raskind, Adriana Rebelo, Chloe M. Reuter, Lynette Rives, Lance H. Rodan, Martin Rodriguez, Jill A. Rosenfeld, Elizabeth Rosenthal, Francis Rossignol, Maura Ruzhnikov, Marla Sabaii, Jacinda B. Sampson, Timothy Schedl, Lisa Schimmenti, Kelly Schoch, Daryl A. Scott, Elaine Seto, Vandana Shashi, Emily Shelkowitz, Sam Sheppeard, Jimann Shin, Edwin K. Silverman, Giorgio Sirugo, Kathy Sisco, Tammi Skelton, Cara Skraban, Carson A. Smith, Kevin S. Smith, Lilianna Solnica-Krezel, Ben Solomon, Rebecca C. Spillmann, Andrew Stergachis, Joan M. Stoler, Kathleen Sullivan, Shamil R. Sunyaev, Shirley Sutton, David A. Sweetser, Virginia Sybert, Holly K. Tabor, Queenie Tan, Arjun Tarakad, Herman Taylor, Mustafa Tekin, Willa Thorson, Cynthia J. Tifft, Camilo Toro, Alyssa A. Tran, Rachel A. Ungar, Adeline Vanderver, Matt Velinder, Dave Viskochil, Tiphanie P. Vogel, Colleen E. Wahl, Melissa Walker, Nicole M. Walley, Jennifer Wambach, Michael F. Wangler, Patricia A. Ward, Daniel Wegner, Monika Weisz Hubshman, Mark Wener, Tara Wenger, Monte Westerfield, Matthew T. Wheeler, Jordan Whitlock, Lynne A. Wolfe, Heidi Wood, Kim Worley, Shinya Yamamoto, Zhe Zhang, and Stephan Zuchner.

Acknowledgments

We thank all families who participated in this study and generously contributed their time and data. S.S. is funded through the Albert Rowe II Endowed Chair in Genetics. The research reported in this manuscript was in part supported by the NIH Common Fund, through the Office of Strategic Coordination/Office of the NIH Director and the National Institute of Neurological Disorders and Stroke of the NIH under award numbers U01HG010218 and U01HG007708. The content is solely the responsibility of the authors and does not necessarily represent the official views of the NIH.

Declaration of interests

D.A.C., L.M.D., and S.V.M. are employees of and may own stock in GeneDx, LLC.

Web resources

GenBank, https://www.ncbi.nlm.nih.gov/genbank/
GeneMatcher, https://genematcher.org/
gnomAD, https://gnomad.broadinstitute.org/
OMIM, https://www.omim.org/

Supplemental information

Supplemental information can be found online at https://doi.org/10.1016/j.ajhg.2025.12.004.

Received: April 1, 2025
Accepted: December 5, 2025
Published: December 29, 2025

References

1. Kawakami, E., Tokunaga, A., Ozawa, M., Sakamoto, R., and Yoshida, N. (2015). The histone demethylase Fbxl11/Kdm2a plays an essential role in embryonic development by repressing cell-cycle regulators. Mech. Dev. *135*, 31–42. https://doi.org/10.1016/j.mod.2014.10.001.
2. Levy, M.A., McConkey, H., Kerkhof, J., Barat-Houari, M., Bargiacchi, S., Biamino, E., Bralo, M.P., Cappuccio, G., Ciolfi, A., Clarke, A., et al. (2022). Novel diagnostic DNA methylation episignatures expand and refine the epigenetic landscapes of Mendelian disorders. HGG Adv. *3*, 100075. https://doi.org/10.1016/j.xhgg.2021.100075.
3. Harris, J.R., Gao, C.W., Britton, J.F., Applegate, C.D., Bjornsson, H.T., and Fahrner, J.A. (2024). Five years of experience in the Epigenetics and Chromatin Clinic: what have we learned and where do we go from here? Hum. Genet. *143*, 607–624. https://doi.org/10.1007/s00439-023-02537-1.
4. Faundes, V., Newman, W.G., Bernardini, L., Canham, N., Clayton-Smith, J., Dallapiccola, B., Davies, S.J., Demos, M.K., Goldman, A., Gill, H., et al. (2018). Histone Lysine Methylases and Demethylases in the Landscape of Human Developmental Disorders. Am. J. Hum. Genet. *102*, 175–187. https://doi.org/10.1016/j.ajhg.2017.11.013.
5. van Jaarsveld, R.H., Reilly, J., Cornips, M.-C., Hadders, M.A., Agolini, E., Ahimaz, P., Anyane-Yeboa, K., Bellanger, S.A., van Binsbergen, E., van den Boogaard, M.-J., et al. (2023). Delineation of a KDM2B-related neurodevelopmental disorder and its associated DNA methylation signature. Genet. Med. *25*, 49–62. https://doi.org/10.1016/j.gim.2022.09.006.
6. Rots, D., Jakub, T.E., Keung, C., Jackson, A., Banka, S., Pfundt, R., De Vries, B.B.A., Van Jaarsveld, R.H., Hopman, S.M.J., Van Binsbergen, E., et al. (2023). The clinical and molecular spectrum of the KDM6B-related neurodevelopmental disorder. Am. J. Hum. Genet. *110*, 963–978. https://doi.org/10.1016/j.ajhg.2023.04.008.
7. Kosmicki, J.A., Samocha, K.E., Howrigan, D.P., Sanders, S.J., Slowikowski, K., Lek, M., Karczewski, K.J., Cutler, D.J., Devlin, B., Roeder, K., et al. (2017). Refining the role of de novo protein-truncating variants in neurodevelopmental disorders by using population reference samples. Nat. Genet. *49*, 504–510. https://doi.org/10.1038/ng.3789.
8. Zhou, X., Feliciano, P., Shu, C., Wang, T., Astrovskaya, I., Hall, J.B., Obiajulu, J.U., Wright, J.R., Murali, S.C., Xu, S.X., et al. (2022). Integrating de novo and inherited variants in 42,607 autism cases identifies mutations in new moderate-risk genes. Nat. Genet. *54*, 1305–1319. https://doi.org/10.1038/s41588-022-01148-2.
9. Fu, J.M., Satterstrom, F.K., Peng, M., Brand, H., Collins, R.L., Dong, S., Wamsley, B., Klei, L., Wang, L., Hao, S.P., et al. (2022). Rare coding variation provides insight into the genetic architecture and phenotypic context of autism. Nat. Genet. *54*, 1320–1331. https://doi.org/10.1038/s41588-022-01104-0.
10. Damianidou, E., Mouratidou, L., and Kyrousi, C. (2022). Research models of neurodevelopmental disorders: The right model in the right place. Front. Neurosci. *16*, 1031075. https://doi.org/10.3389/fnins.2022.1031075.
11. Kour, S., Rajan, D.S., Fortuna, T.R., Anderson, E.N., Ward, C., Lee, Y., Lee, S., Shin, Y.B., Chae, J.-H., Choi, M., et al. (2021). Loss of function mutations in GEMIN5 cause a neurodevelopmental disorder. Nat. Commun. *12*, 2558. https://doi.org/10.1038/s41467-021-22627-w.
12. García-Cazorla, À., Verdura, E., Juliá-Palacios, N., Anderson, E.N., Goicoechea, L., Planas-Serra, L., Tsogtbaatar, E., Dsouza, N.R., Schlüter, A., Urreizti, R., et al. (2020). Impairment of the mitochondrial one-carbon metabolism enzyme SHMT2 causes a novel brain and heart developmental syndrome. Acta Neuropathol. *140*, 971–975. https://doi.org/10.1007/s00401-020-02223-w.
13. Sobreira, N., Schiettecatte, F., Valle, D., and Hamosh, A. (2015). GeneMatcher: a matching tool for connecting investigators with an interest in the same gene. Hum. Mutat. *36*, 928–930. https://doi.org/10.1002/humu.22844.
14. Chen, S., Francioli, L.C., Goodrich, J.K., Collins, R.L., Kanai, M., Wang, Q., Alföldi, J., Watts, N.A., Vittal, C., Gauthier, L.D., et al. (2024). A genomic mutational constraint map using variation in 76,156 human genomes. Nature *625*, 92–100. https://doi.org/10.1038/s41586-023-06045-0.
15. Morales, J., Pujar, S., Loveland, J.E., Astashyn, A., Bennett, R., Berry, A., Cox, E., Davidson, C., Ermolaeva, O., Farrell, C.M., et al. (2022). A joint NCBI and EMBL-EBI transcript set for clinical genomics and research. Nature *604*, 310–315. https://doi.org/10.1038/s41586-022-04558-8.
16. Richards, S., Aziz, N., Bale, S., Bick, D., Das, S., Gastier-Foster, J., Grody, W.W., Hegde, M., Lyon, E., Spector, E., et al. (2015). Standards and guidelines for the interpretation of sequence variants: a joint consensus recommendation of the American College of Medical Genetics and Genomics and the Association for Molecular Pathology. Genet. Med. *17*, 405–424. https://doi.org/10.1038/gim.2015.30.
17. Rentzsch, P., Schubach, M., Shendure, J., and Kircher, M. (2021). CADD-Splice—improving genome-wide variant effect prediction using deep learning-derived splice scores. Genome Med. *13*, 31. https://doi.org/10.1186/s13073-021-00835-9.
18. Ioannidis, N.M., Rothstein, J.H., Pejaver, V., Middha, S., McDonnell, S.K., Baheti, S., Musolf, A., Li, Q., Holzinger, E., Karyadi, D., et al. (2016). REVEL: An Ensemble Method for Predicting the Pathogenicity of Rare Missense Variants. Am. J. Hum. Genet. *99*, 877–885. https://doi.org/10.1016/j.ajhg.2016.08.016.
19. Pejaver, V., Urresti, J., Lugo-Martinez, J., Pagel, K.A., Lin, G.N., Nam, H.-J., Mort, M., Cooper, D.N., Sebat, J., Iakoucheva, L.M., et al. (2020). Inferring the molecular and phenotypic impact of amino acid variants with MutPred2. Nat. Commun. *11*, 5918. https://doi.org/10.1038/s41467-020-19669-x.
20. Carter, H., Douville, C., Stenson, P.D., Cooper, D.N., and Karchin, R. (2013). Identifying Mendelian disease genes with the Variant Effect Scoring Tool. BMC Genom. *14*, S3. https://doi.org/10.1186/1471-2164-14-S3-S3.
21. Feng, B.-J. (2017). PERCH: A Unified Framework for Disease Gene Prioritization. Hum. Mutat. *38*, 243–251. https://doi.org/10.1002/humu.23158.
22. Pejaver, V., Byrne, A.B., Feng, B.-J., Pagel, K.A., Mooney, S.D., Karchin, R., O'Donnell-Luria, A., Harrison, S.M., Tavtigian, S.V., Greenblatt, M.S., et al. (2022). Calibration of computational tools for missense variant pathogenicity classification and ClinGen recommendations for PP3/BP4 criteria. Am. J. Hum. Genet. *109*, 2163–2177. https://doi.org/10.1016/j.ajhg.2022.10.013.

23. Casci, I., Krishnamurthy, K., Kour, S., Tripathy, V., Ramesh, N., Anderson, E.N., Marrone, L., Grant, R.A., Oliver, S., Gochenaur, L., et al. (2019). Muscleblind acts as a modifier of FUS toxicity by modulating stress granule dynamics and SMN localization. Nat. Commun. *10*, 5583. https://doi.org/10.1038/s41467-019-13383-z.
24. Anderson, E.N., Morera, A.A., Kour, S., Cherry, J.D., Ramesh, N., Gleixner, A., Schwartz, J.C., Ebmeier, C., Old, W., Donnelly, C.J., et al. (2021). Traumatic injury compromises nucleocytoplasmic transport and leads to TDP-43 pathology. eLife *10*, e67587. https://doi.org/10.7554/eLife.67587.
25. Anderson, E.N., Gochenaur, L., Singh, A., Grant, R., Patel, K., Watkins, S., Wu, J.Y., and Pandey, U.B. (2018). Traumatic injury induces stress granule formation and enhances motor dysfunctions in ALS/FTD models. Hum. Mol. Genet. *27*, 1366–1381. https://doi.org/10.1093/hmg/ddy047.
26. Pandey, U.B., Nie, Z., Batlevi, Y., McCray, B.A., Ritson, G.P., Nedelsky, N.B., Schwartz, S.L., DiProspero, N.A., Knight, M.A., Schuldiner, O., et al. (2007). HDAC6 rescues neurodegeneration and provides an essential link between autophagy and the UPS. Nature *447*, 859–863. https://doi.org/10.1038/nature05853.
27. Marcogliese, P.C., Deal, S.L., Andrews, J., Harnish, J.M., Bhavana, V.H., Graves, H.K., Jangam, S., Luo, X., Liu, N., Bei, D., et al. (2022). Drosophila functional screening of de novo variants in autism uncovers damaging variants and facilitates discovery of rare neurodevelopmental diseases. Cell Rep. *38*, 110517. https://doi.org/10.1016/j.celrep.2022.110517.
28. Diao, F., Ironfield, H., Luan, H., Diao, F., Shropshire, W.C., Ewer, J., Marr, E., Potter, C.J., Landgraf, M., and White, B.H. (2015). Plug-and-play genetic access to drosophila cell types using exchangeable exon cassettes. Cell Rep. *10*, 1410–1421. https://doi.org/10.1016/j.celrep.2015.01.059.
29. Martin, M. (2011). Cutadapt removes adapter sequences from high-throughput sequencing reads. EMBnet. j. *17*, 10–12. https://doi.org/10.14806/ej.17.1.200.
30. Jühling, F., Kretzmer, H., Bernhart, S.H., Otto, C., Stadler, P.F., and Hoffmann, S. (2016). metilene: fast and sensitive calling of differentially methylated regions from bisulfite sequencing data. Genome Res. *26*, 256–262. https://doi.org/10.1101/gr.196394.115.
31. Waskom, M. (2021). seaborn: statistical data visualization. J. Open Source Softw. *6*, 3021. https://doi.org/10.21105/joss.03021.
32. Blackledge, N.P., Zhou, J.C., Tolstorukov, M.Y., Farcas, A.M., Park, P.J., and Klose, R.J. (2010). CpG Islands Recruit a Histone H3 Lysine 36 Demethylase. Mol. Cell *38*, 179–190. https://doi.org/10.1016/j.molcel.2010.04.009.
33. Frescas, D., Guardavaccaro, D., Kuchay, S.M., Kato, H., Poleshko, A., Basrur, V., Elenitoba-Johnson, K.S., Katz, R.A., and Pagano, M. (2008). KDM2A represses transcription of centromeric satellite repeats and maintains the heterochromatic state. Cell Cycle *7*, 3539–3547. https://doi.org/10.4161/cc.7.22.7062.
34. Lee, P.-T., Zirin, J., Kanca, O., Lin, W.-W., Schulze, K.L., Li-Kroeger, D., Tao, R., Devereaux, C., Hu, Y., Chung, V., et al. (2018). A gene-specific T2A-GAL4 library for Drosophila. eLife *7*, e35574. https://doi.org/10.7554/eLife.35574.
35. Hsieh, T.-C., Bar-Haim, A., Moosa, S., Ehmke, N., Gripp, K.W., Pantel, J.T., Danyel, M., Mensah, M.A., Horn, D., Rosnev, S., et al. (2022). GestaltMatcher facilitates rare disease matching using facial phenotype descriptors. Nat. Genet. *54*, 349–357. https://doi.org/10.1038/s41588-021-01010-x.
36. Dingemans, A.J.M., Hinne, M., Truijen, K.M.G., Goltstein, L., Van Reeuwijk, J., De Leeuw, N., Schuurs-Hoeijmakers, J., Pfundt, R., Diets, I.J., Den Hoed, J., et al. (2023). PhenoScore quantifies phenotypic variation for rare genetic diseases by combining facial analysis with other clinical features using a machine-learning framework. Nat. Genet. *55*, 1598–1607. https://doi.org/10.1038/s41588-023-01469-w.
37. Oegema, R., Barakat, T.S., Wilke, M., Stouffs, K., Amrom, D., Aronica, E., Bahi-Buisson, N., Conti, V., Fry, A.E., Geis, T., et al. (2020). International consensus recommendations on the diagnostic work-up for malformations of cortical development. Nat. Rev. Neurol. *16*, 618–635. https://doi.org/10.1038/s41582-020-0395-6.
38. Dhindsa, R.S., Weido, B., Dhindsa, J.S., Shetty, A.J., Sands, C., Petrovski, S., Vitsios, D., and Zoghbi, A.W. (2022). Genome-wide prediction of dominant and recessive neurodevelopmental disorder risk genes. Preprint at bioRxiv. https://doi.org/10.1101/2022.11.21.517436.
39. Tsukada, Y.i., Fang, J., Erdjument-Bromage, H., Warren, M.E., Borchers, C.H., Tempst, P., and Zhang, Y. (2006). Histone demethylation by a family of JmjC domain-containing proteins. Nature *439*, 811–816. https://doi.org/10.1038/nature04433.
40. Lu, L., Gao, Y., Zhang, Z., Cao, Q., Zhang, X., Zou, J., and Cao, Y. (2015). Kdm2a/b Lysine Demethylases Regulate Canonical Wnt Signaling by Modulating the Stability of Nuclear β-Catenin. Dev. Cell *33*, 660–674. https://doi.org/10.1016/j.devcel.2015.04.006.
41. Tanaka, Y., Yano, H., Ogasawara, S., Yoshioka, S.-I., Imamura, H., Okamoto, K., and Tsuneoka, M. (2015). Mild Glucose Starvation Induces KDM2A-Mediated H3K36me2 Demethylation through AMPK To Reduce rRNA Transcription and Cell Proliferation. Mol. Cell Biol. *35*, 4170–4184. https://doi.org/10.1128/MCB.00579-15.
42. Okamoto, K., Tanaka, Y., Ogasawara, S., Obuse, C., Nakayama, J.-I., Yano, H., and Tsuneoka, M. (2019). KDM2A-dependent reduction of rRNA transcription on glucose starvation requires HP1 in cells, including triple-negative breast cancer cells. Oncotarget *10*, 4743–4760. https://doi.org/10.18632/oncotarget.27092.
43. Yang, C., Qiao, T., Yu, J., Wang, H., Guo, Y., Salameh, J., Metterville, J., Parsi, S., Yusuf, I., Brown, R.H., et al. (2022). Low-level overexpression of wild type TDP-43 causes late-onset, progressive neurodegeneration and paralysis in mice. PLoS One *17*, e0255710. https://doi.org/10.1371/journal.pone.0255710.
44. Kerkhof, J., Rastin, C., Levy, M.A., Relator, R., McConkey, H., Demain, L., Dominguez-Garrido, E., Kaat, L.D., Houge, S.D., DuPont, B.R., et al. (2024). Diagnostic utility and reporting recommendations for clinical DNA methylation episignature testing in genetically undiagnosed rare diseases. Genet. Med. *26*, 101075. https://doi.org/10.1016/j.gim.2024.101075.

The American Journal of Human Genetics, Volume *113*

Supplemental information

De novo variants in *KDM2A* cause a syndromic neurodevelopmental disorder

Eric N. Anderson, Stephan Drukewitz, Sukhleen Kour, Anuradha V. Chimata, Deepa S. Rajan, Senta Schönnagel, Karen L. Stals, Deirdre Donnelly, Siobhan O'Sullivan, John F. Mantovani, Tiong Y. Tan, Zornitza Stark, Pia Zacher, Nicolas Chatron, Pauline Monin, Severine Drunat, Yoann Vial, Xenia Latypova, Jonathan Levy, Alain Verloes, Jennefer N. Carter, Devon E. Bonner, Suma P. Shankar, Jonathan A. Bernstein, Julie S. Cohen, Anne Comi, Deanna Alexis Carere, Lisa M. Dyer, Sureni V. Mullegama, Pedro A. Sanchez-Lara, Katheryn Grand, Hyung-Goo Kim, Afif Ben-Mahmoud, Sidney M. Gospe Jr., Rebecca S. Belles, Gary Bellus, Klaske D. Lichtenbelt, Renske Oegema, Anita Rauch, Ivan Ivanovski, Frederic Tran Mau-Them, Aurore Garde, Rachel Rabin, John Pappas, Annette E. Bley, Janna Bredow, Timo Wagner, Eva Decker, Carsten Bergmann, Louis Domenach, Henri Margot, Undiagnosed Diseases Network, Johannes R. Lemke, Rami Abou Jamra, Julia Hentschel, Heather Mefford, Amit Singh, Udai Bhan Pandey, and Konrad Platzer

Table of contents

Supplemental Note: Case reports of individuals with causative variants in *KDM2A*

Individual 1, c.422A>G, p.(Tyr141Cys), *de novo*

Individual 2, c.704C>T, p.(Pro235Leu), *de novo*

Individual 3, c.850C>T, p.(His284Tyr), *de novo*

Individual 4, c.956G>A, p.(Arg319Gln), *de novo*

Individual 5, c.1571T>G, p.(Phe524Cys), *de novo*

Individual 6, c.1703G>A, (p.Arg568Gln), *de novo*

Individual 7, c.1772T>C, p.(Met591Thr), *de novo*

Individual 8, c.1796G>C, p.(Arg599Pro), *de novo*

Individual 9, c.2327A>G, p.(Lys776Arg), *de novo*

Individual 10, c.2328G>T, p.(Lys776Asn), *de novo*

Individual 11, c.2431C>A, p.(His811Asn), *de novo*

Individual 12, c.58C>T, p.(Arg20*), *de novo*

Individual 13, c.579C>G, p.(Tyr193*), *de novo*

Individual 14, c.1676dup, p.(Ile560Aspfs*71), *de novo*

Individual 15, c.1677delG, p.(Ile560Leufs*32), *de novo*

Individual 16, c.2404dup, p.(Thr802Asnfs*49), heterozygous

Individual 17, c.2667delC, p.(Asp889Glufs*47), *de novo*

Individual 18, c.2809_2812dup, p.(Cys938*), *de novo*

Supplemental Note: Case report of an individual with a variant in *KDM2A* but insufficient evidence for causality

Individual S19, c.2323G>A, p.(Glu775Lys), heterozygous

Figure S1. Pedigrees of all families

Figure S2. Constraint score landscape of KDM genes.

Figure S3. Overview of domain structure and reported variants in the literature in KDM genes.

Table S1. Variant information and classification according to ACMG criteria.

Table S2. *In silico* prediction of missense variants in *KDM2A*.

Table S3. Detailed clinical data of individuals with causative variants in *KDM2A*

Table S4. Detailed clinical data of an individual with variant in *KDM2A* but insufficient evidence for causality

Table S5. Annotation and *in silico* scores of all missense variants in *KDM2A*

Table S6. List of KDM genes

Table S7. Reported variants in the literature in KDM genes

Table S8. Methylation data of the *KDM2A*-related Episignature

References

Supplemental Note: Case reports of individuals with causative variants in *KDM2A*

Individual 1, c.422A>G, p.(Tyr141Cys), *de novo*

This child is the third child of non-consanguineous parents with her older brother and sister being completely fit and well. There is no family history of note. She presented at 8 months of age with poor feeding and weight gain, with her weight falling through the centiles. She also had developmental delay. At 3 years and 8 months, she was speaking in short sentences with fairly good comprehension. She has feeding difficulties and eats mainly pureed foods. She is walking independently but prefers to furniture walk. She has poor balance and coordination. Due to low tone, she has splints on both feet. She is showing some signs of autistic behavior, such as lining up toys and displaying repetitive play. She wears glasses (+1.50DS / -0.25DC @95 in the right eye and a +2.00DS / -0.25DC @80 in the left). She has central hypothyroidism, diagnosed at around 1 year of age. She has dysmorphic features including midface hypoplasia, epicanthic folds, widely spaced and deeply set eyes, low-set and posteriorly rotated ears, saggy cheeks and a thin vermilion of the upper lip.

Individual 2, c.704C>T, p.(Pro235Leu), *de novo*

This 8-year-old boy was the first child of a non-consanguineous couple from the USA. He was born at term weighing 3.4 kg (43rd centile) to a prima gravida 28-year-old mother following a pregnancy complicated by recurrent vaginal bleeding requiring treatment with progesterone and bedrest. He had an unremarkable newborn course except for limited lip and tongue mobility which required a change from breast to bottle-feeding. Parents had concerns for his social indifference, lack of eye contact and absence of reciprocal smiling by 6 weeks of age. He began exhibiting repetitive mannerisms in the first year of life including hand-flapping with excitement, covering his ears, staring at lights, and preferring to hold red objects which he carried with him compulsively. His emotional regulation was poor including periods of whining and crying for no apparent reason for at least half of every day alternating with periods of appearing to be self-absorbed and contented. He was diagnosed with global developmental delay and autism spectrum disorder at 17 months of age. He has not developed speech or gestural communication and communicates his needs by sporadic use of an augmentative communication device and whining or crying. He has had normal brain MRI and biochemical testing including carbohydrate deficient transferrin electrophoresis, plasma amino acids, urine organic acids and mucopolysaccharides, and lactic and pyruvic acid levels.

He was diagnosed with gluten sensitivity on borderline positive autoantibody screening at age 6 years and immune deficiency with reduced levels of IgA and pediatric autoimmune neuropsychiatric disorder associated with streptococcal infection (PANDAS) with elevated ASO titers at age 7 years when he developed symptoms of reduced oral intake, tics, “rage attacks” and self-injurious behaviors without provocation. He has been treated with Intravenous Immunoglobulin (IVIG) with only minimal improvement in tantrums and compulsive behaviors. He has not had seizures or developmental regression.

His examination is positive for height at the 14th centile, 57th centile for weight and 70th centile for OFC, a small chin, prominent vermilion of the upper lip, and slightly enlarged ears.

Individual 3, c.850C>T, p.(His284Tyr), *de novo*

This boy is the only child to his healthy unrelated parents of Anglo-Celtic heritage of average height. He was born by vaginal delivery at term after an uncomplicated pregnancy. He needed brief stimulation and CPAP at birth, but this was only required for less than 24 hours. He was admitted to Special Care Nursery for treatment with IV antibiotics for suspected sepsis because of maternal Group B strep positivity. He had feeding difficulties and slow weight gain. Although his feeding difficulties resolved, he remains a fussy eater and growth restricted.

He started walking at 14 months but concerns were raised about his speech development when his first words were at 2 years. His fine motor skills were also delayed, but social skills were normal. He has always been very active and was assessed with ADHD.

He was first referred to genetics at age 6 years of age. He was noted to be growth restricted with all parameters below the 3rd centile and was of lean build without evidence of lipodystrophy. He had triangular facial features but was otherwise eumorphic. There was a small pigmented macule on his back but no congenital malformations noted on examination.

Individual 4, c.956G>A, p.(Arg319Gln), *de novo*

The 25-year-old female is the eldest daughter of non-consanguineous parents from Afghanistan. Additionally to having three healthy siblings, her mother suffered two miscarriages of unknown cause. The pregnancy is said to have been “normal”. When being a newborn, she was treated for newborn jaundice by phototherapy. The developmental delay was noticed shortly after birth, but the family was unable to provide detailed information regarding developmental milestones. She is said to have learned to walk at two years of age and has never been able to speak. She does understand Persian and communicates non-verbally very skillfully. Her family believes that the problems of speaking are caused by her tongue and possibly describe the clinical presentation of dysarthria.

The epilepsy syndrome with focal onset seizures (aura and focal to bilateral tonic clonic) are said to have started around the age of ten, years before the family left for Germany. It is believed that she feels an epileptic aura, since she always tries to seek contact. After she stares absently and salivates profusely before generalization into a tonic-clonic seizure. On interictal EEG, biparietal focal epileptiform discharges were observed. The brain MRI showed signs of disturbed neuronal migration and organization of cortex in form of changes out of the lissencephaly spectrum (cobblestone malformation/polymicrogyria with anterior maximum, diffuse gliosis, heterotopia, simple ventricular structure). The speech impairment may potentially be caused by a perisylvian syndrome, when taking these brain MRI findings into account. The pharmacoresistant epilepsy was previously treated with valproate and carbamazepine. At present she was treated with levetiracetam and oxcarbamazepine. Upon clinical examination she exhibits upslanted palpebral fissures, a narrow mouth with a thin upper and lower vermilion of the lip, facial asymmetry (after a nasal fracture), a small stature (149 cm, - 3 SD) and conical tapered fingers.

Individual 5, c.1571T>G, p.(Phe524Cys), *de novo*

She is the first child of a non-consanguineous family and has a younger sister. Her mother underwent surgical removal of a cavernoma, and her cousin had a child with an undiagnosed motor handicap. Her niece has been diagnosed with an autism spectrum disorder. She was born at 38 weeks of gestation following a pregnancy marked by intrauterine growth restriction (IUGR). At birth, her weight was 2480 g (8th centile), length 44 cm (1st centile), and OFC 31 cm (2nd centile). During the neonatal period, she experienced feeding difficulties and jaundice. In her first year, she had several seizure episodes associated with fever and required treatment with valproate. She was subsequently tapered off but a recurrence of seizures at age nine required treatment of valproate again. Treatment was then stopped at age 12 years and she remained seizure free since then. Regarding her motor development, she achieved sitting between 12 and 18 months and began walking at 21 months. She started using her first words at 12 months. Initially, she attended a regular school with assistance until the second year of preschool, after which she transitioned to a specialized class. Her OFC growth trajectory ranged between -3 and -4 SD.

At her latest clinical examination at the age of 13, she attended a specialized class for children with difficulties. She struggled to acquire reading skills but could recognize letters. Her healthcare plan included weekly speech therapy sessions and ongoing psychological follow-up. Her weight was 32.5 kg (-3.0 SD), height 153 cm (-0.8 SD), and OFC at 50 cm (-3.5 SD). Clinically, she presented with a triangular face, and a pointed chin. Divergent strabismus was observed. She had arachnodactyly. In the neuropsychological evaluation, her intellectual functioning was heterogeneous, with mild impairment in cognitive functioning and attention deficit disorder. Her verbal comprehension index was 73, visuospatial index 78, fluid reasoning 64, working memory 59, and processing speed 45. Both her EEG and brain MRI showed no abnormal findings. Overall, she exhibited pre- and postnatal microcephaly, developmental delay, and dysmorphic features.

This individual passed away at the age of 14 years due to acute respiratory distress syndrome in the context of a complicated flu.

Individual 6, c.1703G>A, (p.Arg568Gln), *de novo*

This boy is the 2nd child of an unrelated couple. The family history is unremarkable. Pregnancy was characterized by intrauterine growth retardation beginning at 34 weeks of gestation associated with pathological Doppler measurements. He was born at 37 weeks + 2 days of gestation by cesarean section. His birth weight was 1900 g (6th centile), with a height of 45.5 cm (40th centile) and an OFC of 32 cm (25th centile). During the neonatal period, he required enteral feeding for 2 weeks and was hospitalized for 1 month. Since then, he has had persistent feeding difficulties, with severe gastroesophageal reflux. Morphologically, he has a rounded forehead and bilateral epicanthus. Regarding his motor development, he achieved sitting at 1 year and started walking at 18 months. He said his first words at 20 months. He has undergone surgery for cryptorchidism. He has severe learning disabilities, with a significant language impairment associated with an attention deficit disorder and behavioral problems. He attends a regular school with assistance. He has weekly speech therapy and orthoptic sessions and receives psychological support. Growth hormone treatment has been initiated to address the stagnation in his growth. Under treatment, his weight and stature trajectories are ranging between -3 and -5.5 SD. A brain MRI showed no abnormal findings.

Individual 7, c.1772T>C, p.(Met591Thr), *de novo*

The individual was born at 41 weeks via vaginal delivery to a 30-year-old G2P1-2 mother and a 33-year-old father. The pregnancy was notable for maternal GERD and transient polyhydramnios. There were no prenatal exposures. She was born small for gestational age. Birth weight was 2920 g (17th centile, -0.96 SD), birth length was 47 cm (16th centile, -0.98 SD), and birth head circumference was 35 cm (57th centile, 0.18 SD). Apgar scores were 8 and 8 at 1 minute and 5 minutes respectively. Suctioning and brief oxygen was required after delivery. The neonatal period was complicated by jaundice treated with phototherapy. Frenectomy was performed due to tongue tie at 2 days old. She was discharged home on the third day of life.

In infancy she experienced frequent thrush and infections including viral gastroenteritis and bronchiolitis requiring multiple visits to the emergency department. She also has a history of failure to thrive in infancy in the setting of swallowing difficulty, frequent emesis and constipation. She required nasogastric tube feeds from 10 to 12 months old. Her emesis resolved by 2 years old. At 3 months old she was diagnosed with right occipital plagiocephaly in the setting of torticollis. She received physical therapy and wore a helmet between 7-12 months old.

At 11 months old, brain MRI and MRS showed mild nonspecific flattening of the optic discs bilaterally but was otherwise normal. She was seen by ophthalmology, and there were no concerns for papilledema, with normal examination. She has since been diagnosed with alternating exotropia and minimal refractive error, without concerns for amblyopia. Repeat brain MRI at 3 years old showed findings suggestive of Chiari 1 malformation. At 2 years old she was diagnosed with mild bilateral hip dysplasia and underwent bilateral Pemberton osteotomies at 5 years old. Echocardiogram, skeletal survey, abdominal ultrasound, and bone age studies have otherwise been normal.

At 2 years old she developed a facial rash for 9 months, which was treated with topical steroids. At 4 years old she was incidentally found to have Wolff-Parkinson-White syndrome and underwent cardiac ablation at 6 years old.

There were concerns for developmental delay from infancy. She rolled at 5 months old, sat at 9 months old, and crawled at 12 months old and walked unassisted at 20 months old. She spoke her first word at 2 years old. At 3 years old she was diagnosed with autism and ADHD. There have been multiple behavioral symptoms including aggressive behaviors, repetitive behaviors, and sensory sensitivities. She is treated with guanfacine 1 mg. She has been followed by neurology and is noted to have diffuse hypotonia on exam. At 8 years old an EEG was abnormal due to frequent occipital sharp waves over the right occipital region with rare bilateral synchronous occipital discharges. She has never had a seizure nor developmental regression.

She was initially evaluated by genetics at 9 months old. At that time, she was noted to have relative macrocephaly, frontal bossing, short and upturned nose, broad nasal bridge, epicanthus, low set left ear, high anterior hairline, and temporal narrowing. Clinical genetics evaluation has included fragile X testing, chromosomal microarray and trio exome and genome sequencing. Genome sequencing identified a maternally inherited heterozygous variant c.133G>A (p.Gly45Ser) in *HEPACAM* and a maternally inherited heterozygous variant c.4950A>C (p.Gln1650His) in *MED13L*, both of which were classified as uncertain significance. Biochemical testing has been uninformative including plasma amino acids, creatine kinase, urine organic acids, acylcarnitine profile, very long chain fatty acids, and total and free carnitine. Thyroid and growth hormone studies have been normal.

She is of North European and Ashkenazi Jewish ancestry. Consanguinity is denied. She has one older and one younger sister who are reportedly in good health. She has a maternal uncle with syndactyly, pre-axial polydactyly, and learning disabilities. Her maternal grandfather has dementia. Family history is otherwise negative for near relatives with developmental disorders.

She was evaluated through the Undiagnosed Diseases Network (UDN), at 6.5 years old. At that time, she was speaking in phrases and could count to ten. She was in a special education classroom and received adaptive physical education, occupational therapy, physical therapy, and speech therapy at her school. She also receives applied behavioral analysis. Her growth parameters at age 6.5 years old were OFC of 51.0 cm (-0.3 SD), weight of 15.0 kg (-2.5 SD), height of 102 cm (-3.0 SD). Trio genome sequencing was performed through the UDN sequencing core, Baylor Genetics, and was initially resulted negative. Analysis of the variant call sets at the UDN clinical site led to the identification of the apparently *de novo* heterozygous variant c.1772T>C (p.Met591Thr) in *KDM2A* in the proband. Sanger sequencing at Baylor Genetics was performed to confirm the *KDM2A* variant in the proband and segregation in the parents.

Individual 8, c.1796G>C, p.(Arg599Pro), *de novo*

This individual was referred to the genetic center at the age of 10 months old, because of a severe and syndromic IUGR. She is the first and only child of two unrelated and healthy parents. The parents' heights are 164 cm and 179 cm, respectively for the mother and the father. Pregnancy was spontaneous, with no reported teratogenic exposure. NIPT was performed because of an intermediate risk at the first trimester combined screening for trisomy 21. However, because of a family history of trisomy 21 in one of the mother's cousin, and because of a severe IUGR (below 1st centile) diagnosed at 24 weeks of amenorrhea, constitutional and molecular karyotypes were performed on fetal DNA extracted from amniotic fluid: both analyses were normal. Doppler-US were normal. CMV serology was negative in the mother blood. There was no anti-phospholipid syndrome, nor any other autoimmune disease. She was born prematurely by induced vaginal delivery at 36 weeks and 3 days because of the significant IUGR. Adaptation to extra-uterine life was excellent (Apgar 10/10/10/10). Birth weight was 1590 g (0.1^{st} centile), birth length 41.5 cm (1st centile) and a birth OFC of 29.5 cm (2nd centile), confirming the harmonious IUGR. Placenta analyses showed a few calcifications, which may partly, but not entirely, explain the IUGR. CMV was controlled negative in a neonatal urine sample. Transfontanellar US-scan was normal, as well as neonatal hearing tests. However, the baby girl stayed 1 month in the neonatology unit because of this prematurity, and because of oral disorders initially linked to cow's milk proteins allergy complicated with rectorrhagia. Besides, she presented with a few morphological features, including a protruding metopic suture, up-slanted palpebral fissures, bilateral epicanthus, strabismus of the right eye, and a large mouth with a thin vermilion of the lower lip. Bilateral shoulders dimples were also noted. These features were not suggestive of a Silver-Russell syndrome. Moreover, she did not present with hypoglycemia. Temporary teeth eruption was delayed. We performed cardiac US-scan which was normal. Renal US-scan showed a small kidney stone.

Neuropsychological assessment was performed at 37 months of age, confirming a global development delay: developmental ages were estimated about 18 months old for fine motor skills, 24 months old for global motor skills, autonomy and socialization, and 30 months old for language. Neurological examination was normal as well as brain MRI which was performed at 2 years and 10 months old. She still has significant feeding problems. Weight grows around -3.5 SD, height grows around -2.5 DS and OFC grow around -2.5 DS. Growth hormone therapy has recently been introduced, at 4 years of age, even though GH deficiency has not been identified.

Individual 9, c.2327A>G, p.(Lys776Arg), *de novo*

The subject is a 13 year old male born from 29-year-old healthy father and 38-year-old healthy mother, who is high school educated. The parents are non-consanguineous. There is a family history of language delay. His two older brothers are not affected.

He was born at 32 weeks gestation via vaginal delivery after an uneventful pregnancy. There was no delivery or early neonatal complications. He sat and walked independently on time with no loss of acquired motor skills. His babbling was on time; however, he was delayed in speaking baba/mama, single words, or two-word sentence. He has learning disabilities but no intellectual disability, seizures, hyperactivity, inattention, or any behavioral problem. He was diagnosed to have autism at the age 3 years by ADI-R. Previously, he showed the loss of acquired social skills at 24 months but has shown considerable progress at the last assessment at age 13. He demonstrates average abilities in expressing and understanding other people's perspectives, and his communication skills are developing at a steady pace. He shares a good bond with his brothers, although he finds it challenging to connect with unfamiliar children. His ability to build and maintain relationships is also progressing at an average pace, and he shows a similar level of development in sharing interests. He attends a special school designed to cater to the needs of autistic children.

His orientation of time, place and person was normal, and recent memory was unaffected. He had no allergy, and his diet is normal. Special attention is being given to manage his weight. He is not taking any medication. His general appearance was normal as were his head and skin. No CT, brain MRI, or EEG was done.

At the age 3, the Fragile X molecular genetic testing showed one copy of chromosome X with 36 CGG repeats in the 5’-UTR in FMR-1 gene and it was interpreted as normal. Chromosome analysis, tuberous sclerosis, Rett syndrome, Angelman syndrome, Prader-Willi syndrome tests, or metabolic screening were not done.

Individual 10, c.2328G>T, p.(Lys776Asn), *de novo*

This individual is currently a 15-year-old male who was initially referred to Medical Genetics when he was 12-years-old due to a history of auditory processing disorder, motor delays, and epilepsy. His pregnancy was unremarkable. He was born at 34.5 weeks gestation and spent 12 days in the NICU due to prematurity. During his NICU stay, he required a feeding tube, however, he did not require one upon discharge. He did have delays early on, however, exact milestones are unknown. At 12-years-old, he was in a mixed special education and mainstream school program. He was in mainstream classes for history and science. He received speech and occupational therapy. In addition to Medical Genetics, he was followed by Neurology due to epilepsy, ADHD, and auditory processing disorder. He had a history of grand mal seizures for which a vEEG was done and revealed nocturnal episodes of eye opening 3-7x/night with vEEG correlate, suggesting possible nocturnal epilepsy. Brain MRI showed possible hippocampal asymmetry. His medical history is otherwise remarkable for an abnormal sleep schedule with excessive sleep, frequent naps, and snoring for which prior sleep study revealed borderline OSA. He was nondysmorphic. Exome sequencing was sent and revealed the following results:

- *KDM2A* c.2328G>T, p.Lys776Asn *de novo* heterozygous variant of uncertain significance
- *IRF2BP2* c.64C>A, p.Pro22Thr, *de novo* heterozygous variant of uncertain significance
- *SCN2A* c.5672 c.5672T>A, p.Val1891Asp paternally inherited variant of uncertain significance

More recently, he had a vEEG that captured two clinical seizures with right gaze deviation. He had generalized onset on EEG. There were frequent generalized epileptiform discharges.

Individual 11, c.2431C>A, p.(His811Asn), *de novo*

This boy presented with epileptic spasms at 5 months of age, unresponsive to multiple anti seizure medications (ASMs). Spasms came under control when pyridoxine was added one month later and all ASMs were discontinued by 1 year of age. Hypotonia was also noted, and he was slightly delayed in sitting and crawling until appropriate pyridoxine dose was reached. Language development was somewhat accelerated. He had physical therapy for about 2 years as a young child; walking was slightly delayed. He is described as uncoordinated and has difficulty participating in sports due to coordination issues and fatigability. He was eventually tapered off of pyridoxine when he was 6 years of age and remains seizure-free and off medications at age 14 years.

He participated in regular classes in school, though did have an education plan for social and sensory issues in elementary school. He was diagnosed for autism spectrum at 6 years old; he is quite social but has difficulty with social cues in many situations. In addition, he did perseverate until about 13 years. He had therapy for about 2 years as a young child for sensory integration and executive functioning. It is expected that he will be able to live independently as an adult.

Individual 12, c.58C>T, p.(Arg20*), *de novo*

Pregnancy was complicated by low fetal movements. This individual was born at 38 weeks 5 days gestation to a prima gravida mother. He weighed 2438 g with a length of 48 cm long at birth. He had hypoglycemia after birth, which resolved with formula. He always had height and weight in the lower centiles. His weight and height plateaued around 9-10 months of age. Bone age was delayed (bone age of 1 year with SD of 2.7 months with chronological age of 1 year 5 months). Thyroid function studies and IGF-1 and IGFBP-3 have been within normal limits. He was first noted to have hypotonia at 15 months. He had pes planus and increased flexibility of the ankles bilaterally. He had history of constipation, chronic cold, and frequent drooling. At 4 years old, he sustained a right non-displaced supracondylar fracture after a fall.

He was delayed with his developmental milestones. He walked independently around 18/19 months and first words came around 18 months. Speech has progressed well. He has been in special education classes and received developmental therapies. At 6 years old, he was having difficulties with writing and had fine motor delays. He always had behavioral issues and delayed processing speed.

The family history was significant for a younger brother with developmental delays, primarily in speech. Parents were non-consanguineous of Chinese and Dominican descent. Most recent clinical evaluation was at 5 years 11 months old and was significant for short stature, epicanthus, upslanted palpebral fissures, and protruding ears. Weight was 14.7 kg (-2.93 SD), height was 102.5 cm (-2.49 SD), and head circumference was 49.5 cm (-1.47 SD).

Duo exome testing identified the individual to be heterozygous for the variant c.58C>T, p.(Arg20*) in *KDM2A* that proved to of *de novo* origin after targeted testing of the other parent. Mt DNA testing identified the homoplasmic variant of uncertain significance m.7854T>C, p.Val90Ala in the *MT-CO2*. His mother also harbored the m.7854T>C variant in *MT-CO2* at an apparently homoplasmic level. He also had normal male karyotype, microarray, and methylation studies of chromosome 11 for Russell Silver syndrome. SMA copy number analysis showed 2 copies of SMN1.

Individual 13, c.579C>G, p.(Tyr193*), *de novo*

This individual is the product of conception of a cognitively normal non-consanguineous couple with unremarkable family history. The 36 years old father had inflammatory bowel disease and bipolar signs, the 39 years old mother was healthy. Paternal height was 185 cm, maternal height was 152 cm. The two older siblings were reportedly healthy with normal development. First trimester screening at 14 weeks of gestation (wog) was normal including an unremarkable nuchal translucency of 1.8 mm. Follow-up fetal ultrasound at 18 wog revealed intrauterine growth retardation (borderline low weight, abdominal circumference and head measurements just below the 5th centile) and prompted a search for TORCH infections and subsequent amniocentesis. Trisomy-Screening by quantitative-fluorescence-PCR, chromosomal microarray-analysis at a resolution of 100 kb using the Cytoscan HD array (Affymetrix®), methylation sensitive MLPA for the imprinted regions in 11p15 (Silver-Russell syndrome), 7p12.1, 7q32.2 and 14q32.2 revealed normal results for amniotic fluid DNA. Mutational screening by trio exome sequencing on DNA from native amniotic fluid using the Illumina® xGen® Exome Research Panel v2.0 (IDT) and the xGen Human mtDNA Research Panel v1.0 for target capturing and subsequent short-read paired-end sequencing on a NovaSeq 6000 device (Illumina®) revealed no obvious pathogenic variant in an established developmental disease gene. However, the *de novo* nonsense variant in *KDM2A* was considered a strong candidate variant given that the gene codes for a histone lysine demethylase and the known role of similar genes in developmental disorders. Sanger sequencing of DNA from the cultured material and both parents confirmed the *de novo* variant in the fetus. The gene was entered into the GeneMatcher database and due to the prompt and helpful feedback from colleagues who had observed other *de novo* truncating variants in individuals with disabling neurodevelopmental disease, the couple could be counselled accordingly. At 25+2 weeks of gestation, a fetal MRI of the head and whole body was performed, which confirmed microcephaly and did not reveal any other obvious anomaly. After careful consideration and interdisciplinary evaluation and counselling of the parents, the mother's wish to terminate the pregnancy was carried out at 27 weeks of gestation.

Individual 14, c.1676dup, p.(Ile560Aspfs*71), *de novo*

This individual is a 13-year-old boy, first seen in a genetic consultation at the age of 4 years in the context of an acquisition delay. This is the second child of an unrelated, healthy couple. In the family history, there is a deafness in many people in the maternal family. The pregnancy was unremarkable with a vaginal delivery at 41 gestational weeks and 6 days with a good adaptation to extra uterine life. The birth measurements were within the norm. Regarding his development, he was sitting up at 9 months and walking at 18 months. He presented a delay in language, with the beginning of word association at 4 years. He also had difficulties with gross and fine motor skills. Re-educational assessments concluded that he had dyspraxia and ADHD with a cerebellar syndrome. He presented a delay in the acquisition of reading and writing. He benefits from a schooling in an adapted class and has reeducation by speech therapy, psychomotricity and occupational therapy. He has been treated with Methylphenidate for ADHD and Hydroxyzine for anxiety. Complementary ENT examinations were normal, brain MRI was normal and the ophthalmological examination showed mild hyperopia. On clinical examination, at the age of 13 years, he weighed 41.3 kg, was 160 cm tall, and had a head circumference of 53.8 cm (measurements within the norm for age). Cardiac and abdominal examinations were normal. On the skin, there were two café au lait spots on the supraorbital and abdominal areas. A left supernumerary nipple was noted. Neurologically, reflexes were present and sharp. A cutaneous-plantar reflex was in flexion. Walking was correct, as was tightrope walking. Orthopedically, there was an asymmetric pectus excavatum. Genetically, the search for fragile X syndrome was negative, FISH for 22q11 deletion syndrome was also negative and array CGH was normal. The trio exome performed revealed a *de novo* heterozygous frameshift variant in *KDM2A*.

Individual 15, c.1677delG, p.(Ile560Leufs*32), *de novo*

This individual is a 27-year male. He was born at the gestational age of 40 weeks and 1 day, with a low birthweight of 2510 g (2nd centile), length at birth 47 cm (<1st centile), OFC of 33 cm (3rd centile) after an uncomplicated pregnancy. He was operated on multiple cardiac anomalies: pulmonary valve stenosis, small VSD, persistent ductus arteriosus (PDA) and a secundum atrial septal defect, a 4-valved aortic valve with aortic insufficiency and double aortic arch. Moreover he had a duplicated left ureter, and reflux of the right kidney necessitating dilatation and bilateral ureteral reimplantation. Furthermore had an umbilical hernia and developed idiopathic chronic lymphedema of the lower abdomen and scrotal area. As a child he had recurrent otitis media.

His motor development was mildly delayed, with unassisted walking around the age of 2 years. His speech-development was delayed. First words at 18 months. He was in special education and at the ages of 6, 13 and 16 years a disharmonic profile was tested, with a verbal IQ of respectively 95, 80 and 72 and a performal IQ of 60, 61 and 56. A CT-scan of the brain at the age of 16 years was normal. At his current age, his verbal abilities are good and communication is normal.

He has mild dysmorphic features on physical examination, with a micrognathia, frontal upsweep of the hair and mild upslanted palpebral fissures.

His parents are non-consanguineous. He has a healthy older sister. His father has a spontaneously closed ventricle septal defect and premature atrial contractions. A paternal cousin anamnestically is on medication for a heart conduction disorder since the age of 18 years. Both a maternal uncle and maternal aunt have diabetes type 1, which were diagnosed in adulthood. A maternal cousin has diabetes type 2.

At the age of 19 years this individual developed diabetes type 1. Imaging showed a partial pancreatic agenesis and complete gallbladder agenesis. A mild, non-progressive pancytopenia was found repeatedly. Additional genetic testing with a gene-panel diabetes mellitus showed a heterozygous paternally inherited likely pathogenic nonsense variant in *GATA6* (NM_005257.4:c.779C>A, p.(Ser260*)). Although his father has no diabetes, the variant most likely explains, at least partially, this individual's partial pancreatic and complete gallbladder agenesis and hence the development of diabetes. It cannot be excluded that a maternally inherited, not identified, genetic factor is also present, considering the diabetes on the maternal side of the family. Cardiac anomalies can also be seen in individuals with *GATA6* pathogenic variants.

Individual 16, c.2404dup, p.(Thr802Asnfs*49), heterozygous

This male individual was result of a 41 week pregnancy without complications. The family history is unremarkable. Immediately before birth, the mother had a urinary tract and gastrointestinal infection. The birth weight was low (2nd centile) and later a reduced length growth was noted. Apart from this, perinatal and early infantile development were without abnormalities. He learned to sit without support at the age of 6 months, walking without support and other gross-motor skills were acquired age-appropriate as well. Since the age of 3 years, the boy is wearing glasses due to hyperopia and astigmatism. Hearing was good after tympanostomy tubes were inserted due to recurrent tympanic effusions.

Repeated endocrinological examinations due to the short stature revealed growth hormone levels in the lower normal and slightly reduced range, leading to somatotropin substitution since the age of 4 years. Initial language acquisition was reported to be normal. However, abnormalities in speech development emerged in kindergarten, alongside a delay in graphomotor skills was noted. Hence the boy receives occupational and speech therapy. A brain MRI at the age of 4 years revealed unspecific subcortical medullary lesions and a perivenular splenium-associated lesion on the right side. An EEG examination at the age of 5 years showed right frontal, temporo-occipital and left frontal prominent multifocal irregular sharp waves and spikes during sleep, without generalization. No clinical epileptic seizures have occurred to date.

Currently, he visits an integrative kindergarten. It is planned to send him to a regular school soon.

The radiological abnormalities, the speech development disorder and short stature prompted genetic testing with exome sequencing, which identified variants in *ABCD1* and *KDM2A* that were both determined to be of uncertain significance at the time of the initial analysis. The variant in *KDM2A* was later upgraded to likely pathogenic due to the fitting overlap to the rest of the cohort and the knowledge that truncating variants in *KDM2A* cause a neurodevelopmental disorder. Clinical, biochemically and brain MRI findings do not support the diagnosis of Adrenoleukodystrophy, typically associated with pathogenic *ABCD1* variants.

Individual 17, c.2667delC, p.(Asp889Glufs*47), *de novo*

This individual is a 14 year old male first seen due to seizures, intellectual impairments and behavioral concerns. He was born to a 22 year old G1P1 mother. His father was 26 at delivery. Consanguinity was denied. Prenatal exposures included maternal Ondansetron and Paracetamol use and exposure to cats. Pregnancy complications included maternal shingles. Prenatal testing (unclear of what type) showed an increased risk for Down syndrome, and bilateral club foot identified on prenatal ultrasound. He had casting and surgery until about age 4 years for his club feet. He was delivered at 39 weeks via spontaneous vaginal delivery. Birth parameters were length 45.7 cm (-3.0 SD) and weight 2608 g (-2.1 SD). His neonatal course was unremarkable.

His seizures began at the age of 4. This was also when his severe learning impairments were noted. He has been diagnosed with intellectual disability and is in a life skills classroom. He is reported to easily forget information, including spelling words and how to tie his shows. There is a history of regression in school (writing his numbers and letters backwards) and at home (he put on his clothes backwards, reverted back to playing with his little sister's dolls and can't tie his shoes.). He appears to have fairly normal social interactions with his peers and school and has friends. He also has short stature and has been following along the 2nd to 5th centile on the growth chart. However, his most recent height puts him in the 8th centile (152 cm). He wears glasses for myopia but does not wear them all the time. He also has been diagnosed with an astigmatism.

During the last physical exam inverted nipples, delayed coordination for age, instability on heel to toe walk, and mildly impaired balance were noted. His seizures are well controlled. His last seizure occurred at age 12.5 years. Overall his health has been good but his mother reports that he sleeps excessively and has been falling asleep in school. He sleeps through the night and does not snore or exhibit irregular breathing.

Individual 18, c.2809_2812dup, p.(Cys938*), *de novo*

The individual is an 11-year-old boy who was referred for the first time to a clinical geneticist at 16 months due to psychomotor delay and failure to thrive. He is the firstborn of an unrelated couple, conceived via *in vitr*o fertilization. During pregnancy, intrauterine growth retardation was noted around 33 weeks of gestation. Delivery took place at 35+3 weeks of gestation by vaginal delivery due to hypotrophy. He weighed 1.69 kg (-2.7 SD) with a length of 41 cm (-3.0 SD) and an OFC of 31.5 cm (-1.3 SD). He was hospitalized in neonatal care for 3 days due to neonatal jaundice.

Concerning developmental milestones, sitting up was acquired at 14 months age, he walked at 23 months and still only spoke a few words around 4 years. At 9 years he began to form complete sentences, with quite adequate comprehension skills. He demonstrated the ability to read and write simple sentences; however, he experiences difficulties concentrating during class and has approximately a two-year delay in his schooling. He requires a full-time school life assistant.

The physical examination revealed discrete facial characteristics, including low set ears, a thin upper vermilion of the lip with a long philtrum, retrognathia, and a long and narrow nose, along with strabismus. Examination of the extremities showed no remarkable findings. At 11 years old, his height is 139 cm (-0.5 SD), weight is 31.5 kg (-1.0 SD), and OFC is 55 cm (+1.26 SD). He has experienced growth retardation since birth, attributed to growth hormone (GH) deficiency diagnosed at 4 years old, for which he has been successfully supplemented. Premature puberty at 9 years old necessitated treatment with a gonadotropin-releasing hormone (GnRH) analogue to delay puberty.

Surgical correction was performed for cryptorchidism in 2020. In 2021, he required intensive care due to his second episode of pneumonia. Additionally, he experienced recurrent otitis, necessitating three trans-tympanic ventilation tube fittings. Subsequently, long-term antibiotic treatment (trimethoprim/sulfamethoxazole) was prescribed after evidence of CD4+ lymphocytopenia and low IgM levels were observed. Despite a well-conducted vaccination scheme, tetanus, pneumococcal, and diphtheria serology did not demonstrate protective levels.

The cardiac and abdominal ultrasound results were within normal limits; however, a left extra-sinus renal pelvis was detected. Subsequent brain MRI scans revealed normal findings, with the hypophysis noted to be small but within normal parameters. Additionally, bone age assessment showed normal results.

WISC test showed intelligence in the lower range of normal values, Vineland test showed results under first centile. Chromosome 7 methylation analysis ruled out Silver-Russel syndrome. CGH-Array was normal. Whole Exome Sequencing found a *POU1F1* VUS inherited from his mother, also found in his brother (with normal height at 6 years of age), this may have played a role in this GH deficiency.

Supplemental Note: Case report of an individual with a variant in *KDM2A* but insufficient evidence for causality

Individual S19, c.2323G>A, p.(Glu775Lys), heterozygous

This is a female currently 9 years-old with diagnoses of mild intellectual disability, autism spectrum disorder, ADHD, and history of global developmental delay (language, cognitive delays). She was adopted at age 2 months so limited information about her prenatal, birth, and family history is available. She was born at term following a pregnancy without known complications. She did well in the newborn period. She has history of delays in language and cognitive skills, now with diagnoses of mild ID (full scale IQ = 55) and autism spectrum disorder, as well as ADHD. Her growth parameters have been within the normal range, however her head circumference has always been on the larger side. No seizures, medical problems, or organ malformations and no dysmorphic features other than bilateral epicanthus, broad nasal bridge and broad nose were noted.

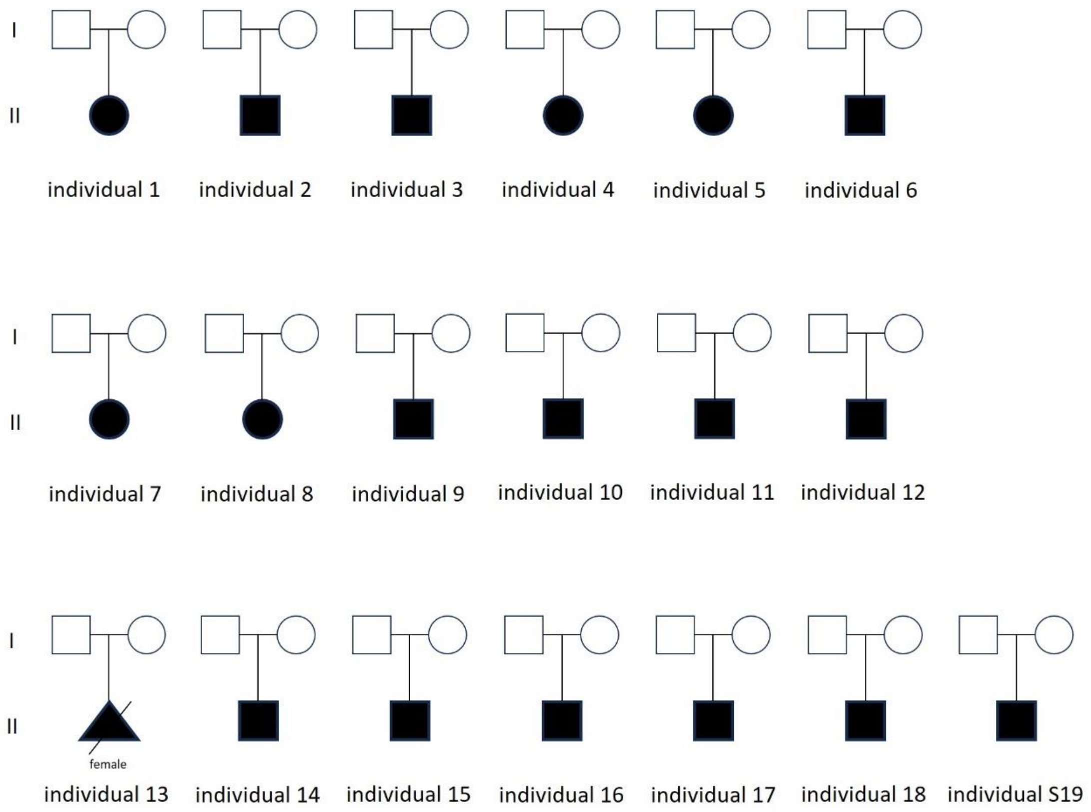

Figure S1. Pedigrees of all families

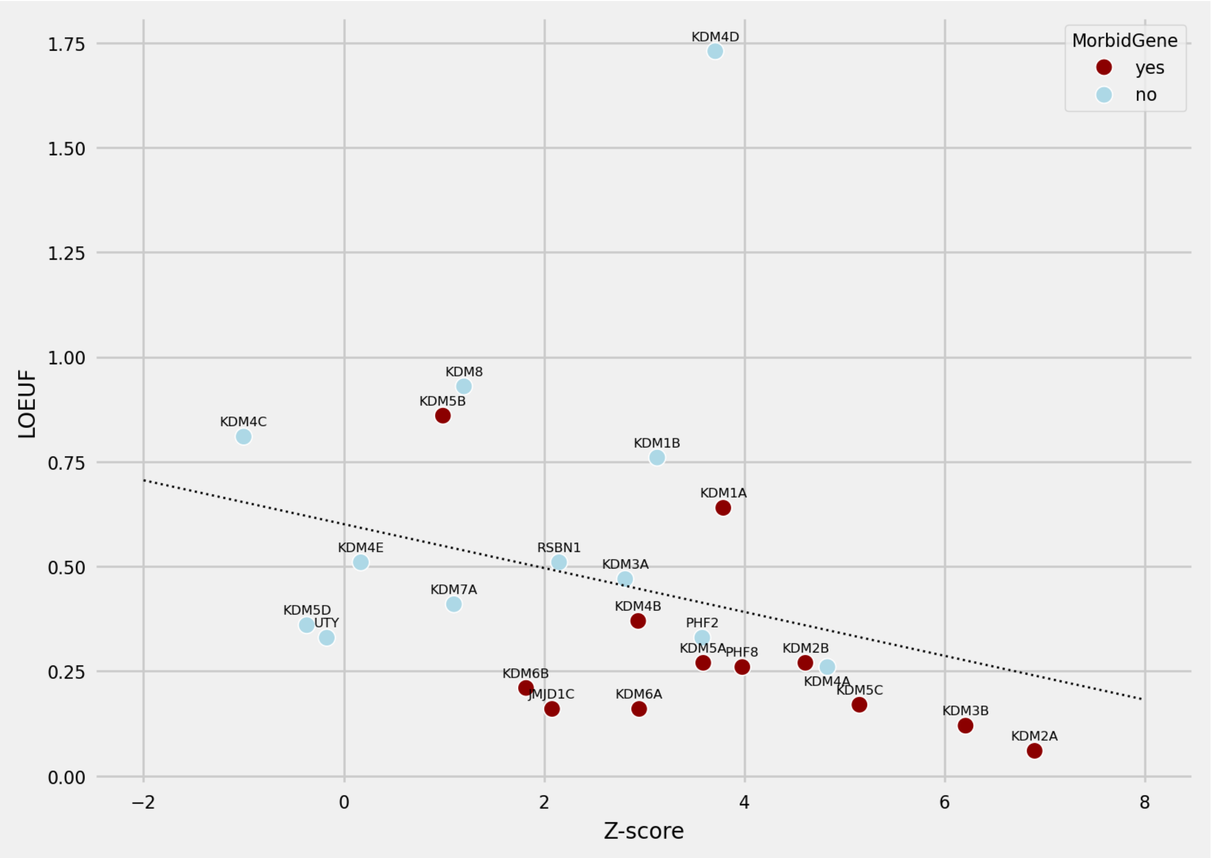

Figure S2. Constraint score landscape of KDM genes. Relationship between the LOEUF (predicted loss-of-function variants) and z-score (missense variants) among all KDM genes. *KDM2A* is the most constrained gene among this group with respect to both z-score and LOEUF, especially among MorbidGenes.[10] Constraint scores where preferably derived from gnomAD v4, but, if not available, also from gnomAD v2.1.1 (Table S6).[7] The dotted line indicates linear regression.

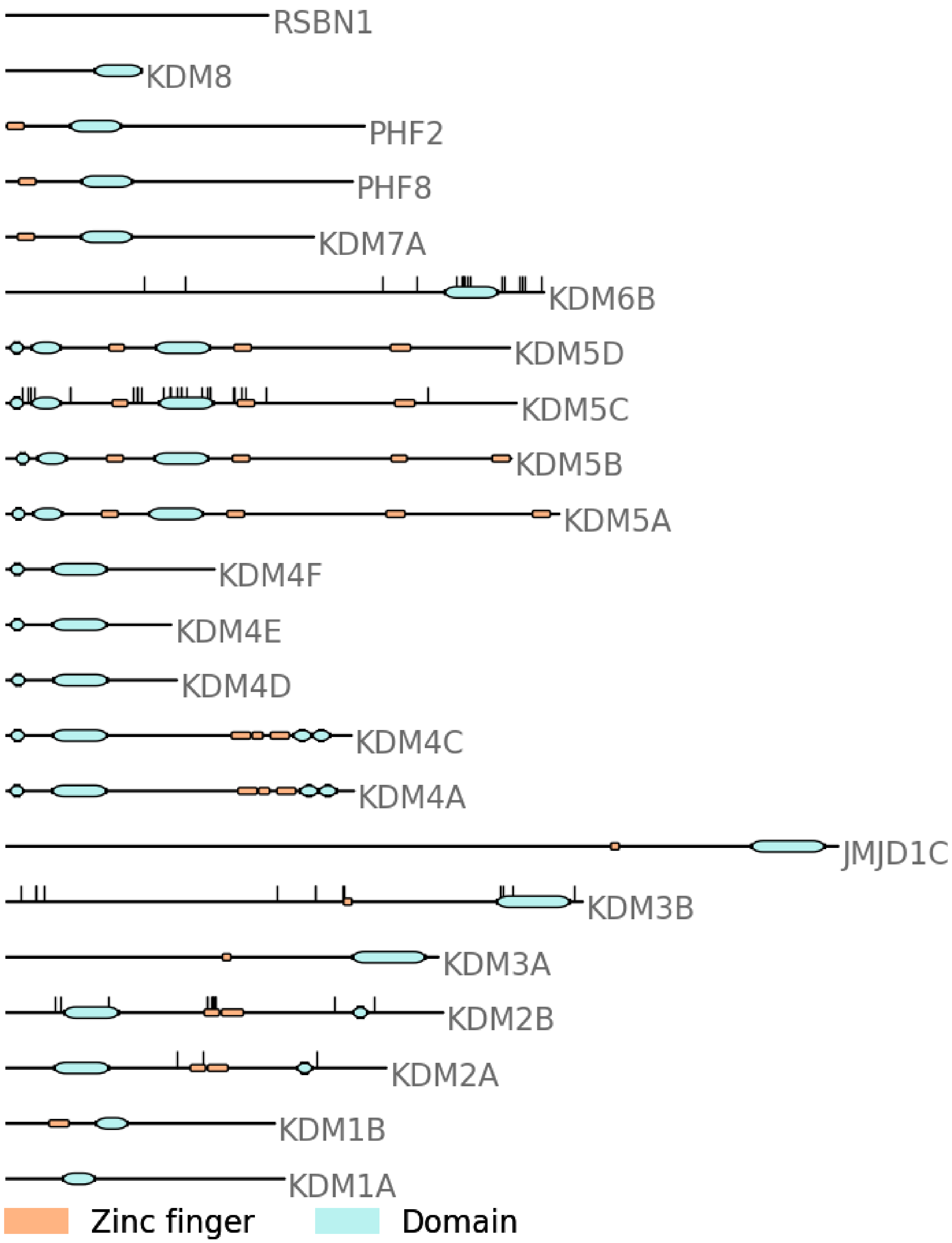

Figure S3. Overview of domain structure and reported variants in the literature in KDM genes. See Table S6 for a list of variants in KDM genes.

individual	Chr11: g.(hg38)	c.	p.	allelic state	origin	predicted effect	ACMG criteria[1]	classification
1	67207624	c.422A>G	p.(Tyr141Cys)	heterozygous	*de novo*	missense	PS2_MOD, PS3_MOD, PM2, PP2, PP3	likely pathogenic
2	67217747	c.704C>T	p.(Pro235Leu)	heterozygous	*de novo*	missense	PS2_MOD, PS3_MOD , PM2, PP2, PP3	likely pathogenic
3	67219296	c.850C>T	p.(His284Tyr)	heterozygous	*de novo*	missense	PS2_MOD, PM2, PP2, PP3	likely pathogenic
4	67219402	c.956G>A	p.(Arg319Gln)	heterozygous	*de novo*	missense	PS2_MOD, PM, PP2, PP3	likely pathogenic
5	67245196	c.1571T>G	p.(Phe524Cys)	heterozygous	*de novo*	missense	PS2_MOD, PM2, PP2	uncertain significance*
6	67245328	c.1703G>A	p.(Arg568Gln)	heterozygous	*de novo*	missense	PS2_MOD, PM2, PP2, PP3	likely pathogenic
7	67245397	c.1772T>C	p.(Met591Thr)	heterozygous	*de novo*	missense	PS2_MOD, PM2, PP2, PP3	likely pathogenic
8	67245421	c.1796G>C	p.(Arg599Pro)	heterozygous	*de novo*	missense	PS2_MOD, PM2, PP2, PP3	likely pathogenic
9	67250357	c.2327A>G	p.(Lys776Arg)	heterozygous	*de novo*	missense	PS2_MOD, PM2, PP2	uncertain significance*
10	67250358	c.2328G>T	p.(Lys776Asn)	heterozygous	*de novo*	missense	PS2_MOD, PM2, PP2	uncertain significance*
11	67250461	c.2431C>A	p.(His811Asn)	heterozygous	*de novo*	missense	PS2_MOD, PS3_MOD, PM2, PP2	likely pathogenic
12	67180094	c.58C>T	p.(Arg20*)	heterozygous	*de novo*	nonsense mediated mRNA decay	PVS1, PS2_MOD, PM2P	pathogenic
13	66982903	c.579C>G	p.(Tyr193*)	heterozygous	*de novo*	nonsense mediated mRNA decay	PVS1, PS2_MOD, PM2	pathogenic
14	67245301dup	c.1676dup	p.(Ile560Aspfs*71)	heterozygous	*de novo*	nonsense mediated mRNA decay	PVS1, PS2_MOD, PM2	pathogenic
15	67245302del	c.1677del	p.(Ile560Leufs*32)	heterozygous	*de novo*	nonsense mediated mRNA decay	PVS1, PS2_MOD, PM2	pathogenic
16	67250434dup	c.2404dup	p.(Thr802Asnfs*49)	heterozygous	unknown	nonsense mediated mRNA decay	PVS1, PM2	likely pathogenic
17	67250697del	c.2667del	p.(Asp889Glufs*47)	heterozygous	*de novo*	nonsense mediated mRNA decay	PVS1, PS2_MOD, PM2	pathogenic
18	67252734_ 67252737dup	c.2809_ 2812dup	p.(Cys938*)	heterozygous	*de novo*	nonsense mediated mRNA decay	PVS1, PS2_MOD, PM2	pathogenic

Table S1. Variant information and classification according to ACMG criteria.[1] MANE Select transcript NM_012308.3 was used. *Due to the *de novo* status of the variant in addition to the fitting clinical overlap to the rest of the cohort, this variant is deemed causative despite being classified as uncertain.

Indi-visual	Chr11: g.(hg38)	c.	p.	CADD-v1.6[2]	REVEL[3]	MutPred2[4]	VEST4[5]	BayesDel[6]	AA conservation (conserved up to)	gnomAD v4[7]
1	67207624	c.422A>G	p.(Tyr141Cys)	32.0	0.836	0.522	0.858	0.389	high (c. elegans)	0
2	67217747	c.704C>T	p.(Pro235Leu)	34.0	0.880	0.594	0.821	0.426	high (zebrafish)	0
3	67219296	c.850C>T	p.(His284Tyr)	27.6	0.954	0.860	0.894	0.567	high (c. elegans)	0
4	67219402	c.956G>A	p.(Arg319Gln)	34.0	0.465	0.426	0.518	0.195	high (c. elegans)	0
5	67245196	c.1571T>G	p.(Phe524Cys)	21.0	0.040	0.334	0.286	-0.099	moderate (platypus)	0
6	67245328	c.1703G>A	p.(Arg568Gln)	32.0	0.473	0.655	0.660	0.271	high (c. elegans)	0
7	67245397	c.1772T>C	p.(Met591Thr)	27.3	0.653	0.615	0.820	0.347	high (zebrafish)	0
8	67245421	c.1796G>C	p.(Arg599Pro)	33.0	0.531	0.611	0.800	0.314	high (frog)	0
9	67250357	c.2327A>G	p.(Lys776Arg)	23.8	0.096	0.298	0.272	-0.081	high (frog)	0
10	67250358	c.2328G>T	p.(Lys776Asn)	23.5	0.109	0.313	0.381	-0.164	high (frog)	0
11	67250461	c.2431C>A	p.(His811Asn)	22.1	0.104	0.479	0.298	-0.234	moderate (chicken)	0

Table S2. *In silico* prediction of missense variants in *KDM2A*.

MANE Select transcript NM_012308.3 was used. *In silico* scores were retrieved using dbNSFP[8]. Cutoffs for *in silico* scores were derived from Pejaver *et al.*[9] Red color signifies damaging prediction (at least PP3_SUP score in Pejaver *et al.* was reached); yellow color signifies neither PP3_SUP nor BP4_SUP score was reached; green color signifies benign prediction (at least BP4_SUP score in Pejaver *et al.* was reached). For further annotations on the missense variants, see Table S5 in the separate excel file.

Table S3. Detailed clinical data of individuals with causative variants in *KDM2A*

See separate Excel file.

Table S4. Detailed clinical data of an individual with variant in *KDM2A* but insufficient evidence for causality

See separate Excel file.

Table S5. Annotation and *in silico* scores of all missense variants in *KDM2A*

See separate excel file. All annotations were retrieved using dbNSFP v4.5.[8]

Table S6. List of KDM genes

See separate Excel file.

Table S7. Reported variants in the literature in KDM genes

See separate Excel file.

Table S8. Methylation data of the *KDM2A*-related Episignature

See separate Excel file. Differential methylated regions between cases and control group. DMRs were filtered for an adjusted p-value of <0.01 and minimal methylation difference of >10%.

References

1. Richards, S., Aziz, N., Bale, S., Bick, D., Das, S., Gastier-Foster, J., Grody, W.W., Hegde, M., Lyon, E., Spector, E., et al. (2015). Standards and guidelines for the interpretation of sequence variants: a joint consensus recommendation of the American College of Medical Genetics and Genomics and the Association for Molecular Pathology. Genetics in Medicine *17*, 405–424. https://doi.org/10.1038/gim.2015.30.

2. Rentzsch, P., Schubach, M., Shendure, J., and Kircher, M. (2021). CADD-Splice—improving genome-wide variant effect prediction using deep learning-derived splice scores. Genome Med *13*, 31. https://doi.org/10.1186/s13073-021-00835-9.

3. Ioannidis, N.M., Rothstein, J.H., Pejaver, V., Middha, S., McDonnell, S.K., Baheti, S., Musolf, A., Li, Q., Holzinger, E., Karyadi, D., et al. (2016). REVEL: An Ensemble Method for Predicting the Pathogenicity of Rare Missense Variants. The American Journal of Human Genetics *99*, 877–885. https://doi.org/10.1016/j.ajhg.2016.08.016.

4. Pejaver, V., Urresti, J., Lugo-Martinez, J., Pagel, K.A., Lin, G.N., Nam, H.-J., Mort, M., Cooper, D.N., Sebat, J., Iakoucheva, L.M., et al. (2020). Inferring the molecular and phenotypic impact of amino acid variants with MutPred2. Nat Commun *11*, 5918. https://doi.org/10.1038/s41467-020-19669-x.

5. Carter, H., Douville, C., Stenson, P.D., Cooper, D.N., and Karchin, R. (2013). Identifying Mendelian disease genes with the Variant Effect Scoring Tool. BMC Genomics *14*, S3. https://doi.org/10.1186/1471-2164-14-S3-S3.

6. Feng, B.-J. (2017). PERCH: A Unified Framework for Disease Gene Prioritization. Human Mutation *38*, 243–251. https://doi.org/10.1002/humu.23158.

7. Chen, S., Francioli, L.C., Goodrich, J.K., Collins, R.L., Kanai, M., Wang, Q., Alföldi, J., Watts, N.A., Vittal, C., Gauthier, L.D., et al. (2024). A genomic mutational constraint map using variation in 76,156 human genomes. Nature *625*, 92–100. https://doi.org/10.1038/s41586-023-06045-0.

8. Liu, X., Li, C., Mou, C., Dong, Y., and Tu, Y. (2020). dbNSFP v4: a comprehensive database of transcript-specific functional predictions and annotations for human nonsynonymous and splice-site SNVs. Genome Medicine *12*, 103. https://doi.org/10.1186/s13073-020-00803-9.

9. Pejaver, V., Byrne, A.B., Feng, B.-J., Pagel, K.A., Mooney, S.D., Karchin, R., O'Donnell-Luria, A., Harrison, S.M., Tavtigian, S.V., Greenblatt, M.S., et al. (2022). Calibration of computational tools for missense variant pathogenicity classification and ClinGen recommendations for PP3/BP4 criteria. The American Journal of Human Genetics *109*, 2163–2177. https://doi.org/10.1016/j.ajhg.2022.10.013.

10. Jauss, R.-T., Popp, B., Bachmann, J., Abou Jamra, R., and Platzer, K. (2024). The MorbidGenes panel: a monthly updated list of diagnostically relevant rare disease genes derived from diverse sources. Hum. Genet. https://doi.org/10.1007/s00439-024-02711-z.
